# Analysis of the *Aedes albopictus* C6/36 genome provides insight into cell line utility for viral propagation

**DOI:** 10.1093/gigascience/gix135

**Published:** 2018-01-10

**Authors:** Jason R Miller, Sergey Koren, Kari A Dilley, Vinita Puri, David M Brown, Derek M Harkins, Françoise Thibaud-Nissen, Benjamin Rosen, Xiao-Guang Chen, Zhijian Tu, Igor V Sharakhov, Maria V Sharakhova, Robert Sebra, Timothy B Stockwell, Nicholas H Bergman, Granger G Sutton, Adam M Phillippy, Peter M Piermarini, Reed S Shabman

**Affiliations:** 1 J. Craig Venter Institute, 9714 Medical Center Drive, Rockville, MD 20850, USA; 2 College of Natural Sciences and Mathematics, Shepherd University, Shepherdstown, WV 25443, USA; 3 Genome Informatics Section, Computational and Statistical Genomics Branch, National Human Genome Research Institute, Bethesda, MD 20892, USA; 4 NCBI/NLM/NIH, 45 Center Drive, Bethesda, MD 20894, USA; 5 USDA 10300 Baltimore Ave., Bldg 306 Barc-East, Beltsville, MD 20705-2350, USA; 6 Department of Pathogen Biology, School of Public Health and Tropical Medicine, Southern Medical University, Guangzhou 510515, China; 7 Department of Biochemistry and the Fralin Life Science Institute, Virginia Tech, Blacksburg, VA, USA; 8 Department of Entomology and the Fralin Life Science Institute, Virginia Tech, Blacksburg, VA, USA; 9 Laboratory of Ecology, Genetics and Environmental Protection, Tomsk State University, Tomsk, Russia; 10 Icahn School of Medicine at Mount Sinai, New York, NY 10029, USA; 11 NBACC, Fort Detrick, MD 21702, USA; 12 Department of Entomology, The Ohio State University, Ohio Agricultural Research and Development Center, Wooster, OH 44691, USA; 13 ATCC, 217 Perry Parkway, Gaithersburg, MD 20877, USA

**Keywords:** *Aedes albopictus*, C6/36, cell line, genome assembly

## Abstract

**Background:**

The 50-year-old *Aedes albopictus* C6/36 cell line is a resource for the detection, amplification, and analysis of mosquito-borne viruses including Zika, dengue, and chikungunya. The cell line is derived from an unknown number of larvae from an unspecified strain of *Aedes albopictus* mosquitoes. Toward improved utility of the cell line for research in virus transmission, we present an annotated assembly of the C6/36 genome.

**Results:**

The C6/36 genome assembly has the largest contig N50 (3.3 Mbp) of any mosquito assembly, presents the sequences of both haplotypes for most of the diploid genome, reveals independent null mutations in both alleles of the Dicer locus, and indicates a male-specific genome. Gene annotation was computed with publicly available mosquito transcript sequences. Gene expression data from cell line RNA sequence identified enrichment of growth-related pathways and conspicuous deficiency in aquaporins and inward rectifier K^+^ channels. As a test of utility, RNA sequence data from Zika-infected cells were mapped to the C6/36 genome and transcriptome assemblies. Host subtraction reduced the data set by 89%, enabling faster characterization of nonhost reads.

**Conclusions:**

The C6/36 genome sequence and annotation should enable additional uses of the cell line to study arbovirus vector interactions and interventions aimed at restricting the spread of human disease.

## Background

Insect cell lines such as Aag2 and C6/36 are critical platforms for insect biology and virology. The *Aedes albopictus* clone C6/36 (ATCC CRL-1660) cell line is commonly used for detection, propagation, and analysis of arboviruses, including antibody-based detection of viruses in saliva (reviewed in [1]). C6/36 cells have a short population doubling time and are permissive to infection by mosquito-transmitted viruses across members of the *Togaviridae, Flaviviridae*, and *Bunyaviridae* families. In particular, C6/36 cells are used to study viruses that pose significant threats to human health, including Zika, dengue, chikungunya. Virus propagation in C6/36 cells guides the rational development of vaccines and therapeutics. PubMed [2] lists 671 publications with C6/36 in the title or abstract.

The progenitor of the C6/36 cell line was established in 1967 from freshly hatched *Aedes albopictus* larvae of unspecified ancestry [3]. The C6/36 subclone was selected for its uniformly high virus yield and was shown to retain a diploid karyotype with 2n = 6 chromosomes in a majority of cells [4]. The similar or equivalent ATC-15 cells [1] were shown to be diploid [5] and to have more chromosomal abnormalities after 110 passages than after 17 [6]. The C6/36 cell line, available through the American Type Culture Collection (ATCC; Manassas, VA, USA), is described as maintaining a diploid chromosome number and being non-anchorage-dependent and nontumorigenic [7]. Despite the widespread use of this cell line to both propagate arboviruses and to use them as a tool to study virus-mosquito interactions, little has been published about features that differentiate the cell line genome from that of *Aedes albopictus* mosquitoes.

Two strains of *A. albopictus* have published genomes, both of which were sequenced on Illumina platforms and assembled with the SOAPdenovo assembler [8]. Sequencing of the Italian Fellini, i.e., Rimini, strain yielded small contigs with N50 < 1 Kbp [9]. The assembly of a Foshan female from China [10], as provided in VectorBase [11], version AaloF1, has a 1.92-Gbp scaffold span, 1.78-Gbp contig span, and 18.4-Kbp contig N50. A third strain was analyzed for its genomic repeats using a pipeline called dnaPipeTE that runs on Illumina reads [12]. The *A. aegypti* Liverpool genome was assembled to draft status from Sanger reads [13] and later de-duplicated (removing putative redundant contigs) and extended to chromosome-length scaffolds with Hi-C technology [14]. The 2014 update in VectorBase has an 82-Kbp contig N50. Using these assemblies, the within-genus divergence between *A. albopictus* and *A. aegypti* was estimated at 71.4 mya [10]. High population heterozygosity has been recognized in mosquitoes for more than 35 years [15], indicating the C6/36 cells could harbor a heterozygous genome.

Recent advances in DNA sequencing technology have enabled the generation of megabase-scale contigs. The Pacific BioSciences (PacBio, Menlo Park, CA, USA) and Oxford Nanopore (Oxford, UK) single-molecule sequencing platforms can generate reads in excess of 10 Kbp. Due to its randomness, the high base call error in PacBio reads can be overcome by using sequencing depths in the ×50 range [16]. New assembly algorithms targeting deep-coverage PacBio data have separated the haplotypes from heterozygous regions of diploid genomes [17].

Here we describe findings for the *Aedes albopictus* C6/36 cell line including its karyotype, the assembly of its genome from PacBio sequence, an analysis of the haplotype separation in contigs, the gene annotation based on public mosquito RNA sequence, and analysis of gene expression based on cell line RNA. We also demonstrate use of the genome and transcriptome for the purpose of subtracting host sequence during an RNA sequencing assay for viruses. The sequence data were previously deposited in public databases to facilitate research on Zika and other viruses commonly transmitted by *Aedes albopictus* and studied in C6/36 cell lines.

## Data Description

The assembly of the genome sequence is available at NCBI [18] under accession GCA_0 018 76365.2. The contig accessions start at MNAF02000000 (equivalent RefSeq accessions are listed in Table S18). The annotation describing the transcripts and genes is available at NCBI under *Aedes albopictus* Annotation Release 101. The sequencing reads used to generate, validate, and analyze the assembly are available at NCBI SRA (see Tables S1 and S2 for accessions). The assembly and annotation are also available at VectorBase [11] under the name canu_80X_arrow2.2.

## Analysis

**Figure 1:**
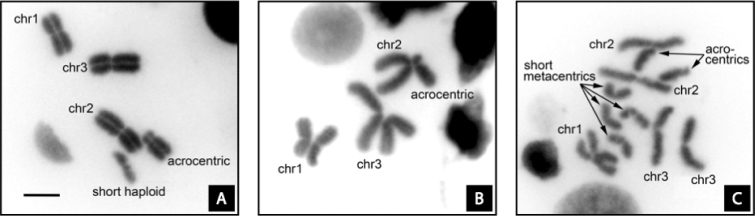
Three karyotypes of the C6/36 *Ae. albopictus* cell line. Chromosomes are labeled chr1 for the shortest, chr2 for the longest, and chr3 for the intermediate-size chromosomes within each image. A, Cell has 3 normal paired chromosomes. An additional acrocentric chromosome pair has a short arm, indicating deletion or translocation elsewhere. An additional short haploid chromosome is unpaired. B, Cell has chromosomes with pairs slightly separated. Chr1 appears normal. The other chromosomes are abnormal, possibly due to translocation of most of 1 arm of chr2 to 1 arm of chr3. C, Cell with chromosome pairs separated. Chr1, chr2, and chr3 appear normal. The chr1 homologous pairs overlap. There are several additional short metacentric and acrocentric chromosomes, shown by arrows. The 5-μm scale bar applies to all 3 images.

C6/36 cells, obtained from ATCC (CRL-1660) and cultured at The J. Craig Venter Institute (JCVI), were subjected to visual analysis of stained metaphase chromosomes to ascertain the karyotype. All cells examined displayed the 3 metacentric chromosomes expected of mosquito cells. A majority of cells also displayed additional chromosomes, as shown in Fig. 1, and the specific composition varied per cell. This analysis suggested variable and cell-specific partial duplications of chromosomes. Notably, a whole-genome duplication was not indicated.

### Sequencing and assembly generate large contigs

Genomic long-read sequencing for assembly-generated 161 Gbp in 17.9 M total reads providing 147 Gbp in 12.4 M reads 5 Kbp or longer and 107 Gbp in 7.10 M reads 10 Kbp or longer (see Table S1 for accessions). Genomic short-read sequencing for analysis generated 45.8 Gbp in 152 M pairs of 2 × 150-bp reads. Transcript sequencing yielded 16.6 Gbp in 27.7 M pairs of 2 × 300-bp partially overlapping reads (see Table S2 for accessions).

Ten candidate assemblies were generated by combinations of 4 software packages: either the Falcon or the Canu assembler [17,19], followed by 0–2 iterations of either the Quiver or Arrow consensus polisher [20]. As shown in Table S3, the 10 resulting assemblies had similar size profiles. The sum of bases was 2.25 Gbp in all assemblies. The average long-read coverage was ×72. Each assembly mapped 93% of the ×20 paired short reads, which had not been used during assembly (Table S4). Local alignments aligned the assemblies’ entire spans in large segments. For example, the contigs of Falcon and Canu after 2 rounds of Arrow each had 2.24 Gbp in alignments of at least 99% sequence identity, and these aligned spans had 630 Kbp N50.

The assembly chosen for downstream analysis was the one from Canu plus 2 rounds of Arrow because its 93.2% short-read map rate was highest by a small margin. This assembly had a total span of 2.247 Gbp in 2434 contigs and a contig N50 of 3.304 Mbp (Table S5). This assembly is available in GenBank with accession GCA_0 018 76365.2. To our knowledge, the C6/36 contig N50 exceeds that of any other assembly of any mosquito or mosquito cell line genome, though some other assemblies offer scaffolds and chromosome mappings in addition to contigs (Table S6).

Accuracy of the C6/36 consensus was inferred from the mapping of short reads, which were not used to generate the assembly. The mapped reads covered 98.98% of assembled bases and confirmed 99.30% of aligned bases; 74.66% of mapped reads aligned end-to-end with 0 mismatches and indels. These results are consistent with prior analyses, e.g., 99.98% identity to the *Drosophila melanogaster* reference achieved by a Canu+Quiver assembly of ×90 P5C3 PacBio [19].

### Dissimilarity with other mosquito assemblies

The C6/36 assembly was compared with *Aedes albopictus* Foshan [10]. The C6/36 contig span is 28% larger. Global alignments spanned 816 Mbp (stringent) or 1.44 Gbp (permissive) of both assemblies (Table S7). Local alignments covered 692 Mbp of Foshan contigs and 1028 Mbp of C6/36 contigs (Table S8). Thus, both alignment methods left large portions of both assemblies unaligned. Sequence identity within alignments was low. The local alignments with at least 95% sequence identity covered only 373 Mbp of Foshan and 596 Mbp of C6/36. Local alignments covered more of C6/36 than Foshan, indicating that some sequences are present at higher multiplicity in the C6/36 assembly. To explore the Foshan vs C6/36 genome difference free of the C6/36 assembly, the C6/36 short read pairs were mapped to Foshan contigs. As shown in Table S9, 93% of pairs had mapped to C6/36 contigs, but only 49% mapped to Foshan contigs. Of pairs not mapped to C6/36, less than 1% mapped to Foshan. These results combine to indicate dissimilarity of the Foshan and C6/36 genomes. It is possible that the cell line is derived from an *A. albopictus* strain that was itself diverged from Foshan. Inter-strain genome size differences had been noted in this species prior to the sequencing era [21].

Inter-species nucleotide alignment was unproductive. Permissive global alignments to *Aedes aegypti* [13,14] covered only 15% (290 Mbp) of Foshan and 14% (304 Mbp) of C6/36.

C6/36 repeats were detected, characterized, and mapped back to the assembly with the process used for Foshan [10]. As shown in Table S10, results were similar to those reported for Foshan. Repeats cover 74% of C6/36 (and 76% of Foshan), and the 3 most abundant repeat types accounted for 59% of assembled bases (and 60% of Foshan bases). The most abundant repeat types were unknown, LINE retrotransposon, and DNA transposon in C6/36 (and LINE, LTR retrotransposon, and other in Foshan).

### Redundancy indicates haplotype separation

**Figure 2:**
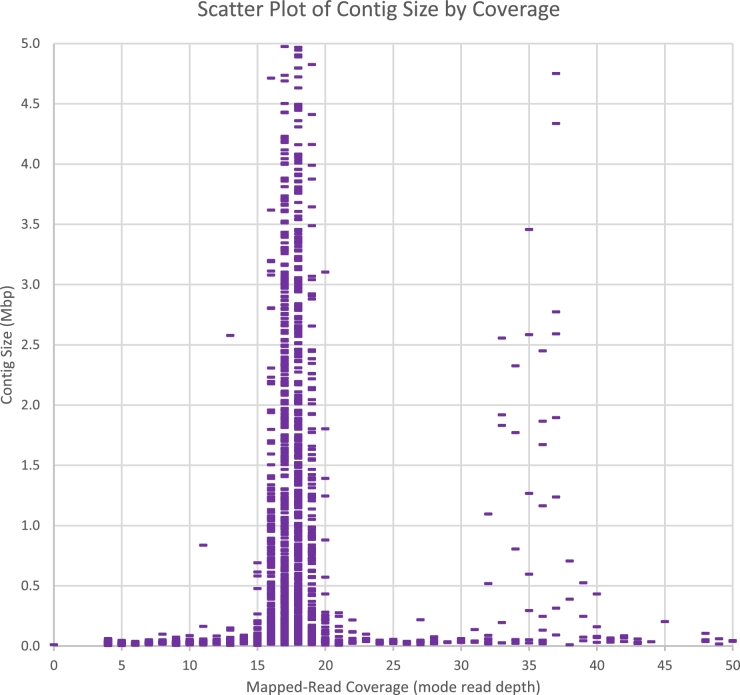
Contig size vs coverage. Each bar in the scatter plot represents 1 contig to which short reads were mapped. The 1832 contigs with mode coverage in the ×18 ± ×6 range collectively span 95.82% of the assembled bases, but there are a few large contigs with ×36 ± ×6 coverage. The apparent bimodal distribution suggests that the ∼×18 contigs could contain separate representations of heterozygous loci, while the ∼×36 contigs could contain the consensus of both alleles from less heterozygous loci.

The Canu assembler can separate haplotype regions that have more than 2% divergence [19]. Therefore, we evaluated the C6/36 assembly for haplotype separation. The first evaluation used the genomic short reads, which were not used by the assembler. K-mer analysis of the short reads, independent of the assembly, estimated 5.7% heterozygosity within the C6/36 genome and a genome size of less than half the assembled contig span (see Fig. S1A). Mapping the reads to contigs yielded an overall coverage mode of ×18 (Fig. S1B). The coverage mode for most individual contigs was also about ×18 (see Fig. 2; Table S11). The bimodal coverage is similar to that seen in the *Quercus lobata* (oak) genome assembly (Fig. 2B in Sork et al. [22]), for which the assembler putatively separated the haplotypes at divergent loci. In C6/36, the 1832 contigs whose mode coverage was in the ×18 ± ×6 range collectively span 95.82% of the assembled bases. There is a smaller group of large contigs with mode coverage in the ×36 ± ×6 range. These results support the hypothesis that most of the C6/36 diploid genome is represented twice in the assembly, possibly due to haplotype separation at heterozygous loci, such that collapsed sequences from less heterozygous loci attracted twice as much short-read coverage.

Second, the C6/36 assembly was tested for the presence of sequence-similar contigs. After alignment of the nucleotide sequence to itself was inconclusive, short reads were mapped allowing up to 4 maps per pair, and the 15% of read pairs that mapped exactly twice were used to identify paired contigs (PCs), defined here as 2 contigs sharing at least 10 000 read pairs that mapped twice. This identified the 529 PCs described in Table S12. There were 708 contigs in PCs (474 contigs in exactly 1 PC and maximum 8 PCs for 1 contig). There were 689 PC contigs with short-read coverage modes in the ×18 ± ×6 range (maximum coverage was ×51). The PCs incorporated 1.97 Gbp or 88% of the 2.25 Gbp assembly. Sequence similarity within PCs was low: average 93.5% identity in aligned bases, with 28% of the sequence aligned (274 Mbp in alignments covering 548 Mbp on contigs). Where PC alignments left 1 contig extending past the other, it was possible to “walk” from PC to PC, as illustrated in Fig. S2. One walk, involving 9 PCs and 9 contigs, all with ×18 ± ×6 coverage, spanned about 18 Mbp total (Fig. S2D). These walks point to duplicated sequences, longer than individual contigs, that are present twice with low similarity, with each copy represented within a different group of adjoining contigs. As PCs incorporate most of the assembly, the duplication most likely represents spans from homologous chromosomes that were assembled to into separate contigs.

As a third evaluation, the C6/36 contigs were subjected to BUSCO analysis [23] using genes thought to have single-copy orthologs across arthropods. Of 2624 genes found in contigs, 64% appeared as 2 instances. As shown in Table 1, 99.6% of 2-instance genes involved ×18 ± ×6 contigs. There was a significant association of genes having 2 instances in the assembly and having all instances on ×18 ± ×6 contigs (Fisher exact test, *P* < 0.01) (Table S14). Thus, the gene duplication mirrors the redundancy observed in PCs.

**Table 1:** BUSCO gene analysis.

No. of Instances	Genes found	Genes on contigs with ×18	Genes on contigs with ×36	Genes only on contigs with ×18	Genes only on contigs with ×36	Instances on contigs with ×18	Instances on contigs with ×36	Instances on other contigs
1	825	704	113	704	113	704	113	8
2	1668	1662	57	1600	2	3262	59	15
3	98	98	9	80	0	275	10	9
4	24	24	1	23	0	94	2	0
5–8	9	9	0	9	0	57	0	0
Total	2624	2497	180	2416	115	4392	184	32

BUSCO genes are presumed single-copy in eukaryotic genomes, but most occur twice in the C6/36 assembly. BUSCO arthropod genes were searched against C6/36 contigs. Two instances were found for 1668 genes. The genes were further evaluated for whether any of their instances occurred in contigs with short-read coverage in the ×18 ± ×6 or ×36 ± ×6 range. These coverage values suggest haplotype separation and collapse, respectively, within the contig sequences. Of genes with exactly 2 instances in the assembly, 1662 (99.6%) had at least 1 instance on a ×18-range contig, while only 57 had at least 1 instance on a ×36-range contig. This supports the characterization of ×18-range contigs as containing sequences specific to a haplotype. Table S13 gives the coordinates and short-read coverage of every instance.

The situation appears nuanced for the 825 1-copy genes. Almost 14% of 1-copy genes mapped to contigs with ×36 ± ×6 coverage, suggesting that these genes are on diploid contigs represented by a consensus of 2 alleles. Another 1% (7 genes) hit contigs with very high (over ×40) coverage, suggesting that these genes may be replicated in the genome but underrepresented in the assembly. Most single-copy genes, 85%, mapped to contigs with ×18 ± ×6 coverage, similar to the portion that mapped to PC contigs. Genes mapping to only 1 contig of a PC, suggesting a “missing gene,” were inspected further. The mapped loci did not show elevated short-read coverage, as would be expected if the gene were higher copy in the genome than the assembly. The alignments did not involve contig ends, as would be expected if the cognate gene belonged in an assembly gap, or unusual levels of discontinuity. Some “missing genes” actually did have fragmentary alignments, suggesting gene loss. As illustrated in Table S15, PC#1, the PC with the most shared short reads, spans 17 BUSCO genes. Its 2-copy genes are ordered consistently across 6 Mbp, but these are interspersed by 4 genes found on only 1 or the other of the 2 contigs, plus 1 gene with additional copies elsewhere in the assembly. Thus, this PC presents syntenic sequences spanning structural variants and indicates that some single-copy BUSCO genes on ×18 ± ×6 contigs are attributable to allele-specific gene loss.

By the 3 methods of read mapping, contig alignment, and gene finding, we consistently found duplication within the assembly. The results are consistent with a model of a heterozygous genome for which spans from both haplotypes are represented in the assembly. This model predicts the genome size is 52% of the assembly size, or 1.172 Gbp. Alternate models have less support. A whole-genome duplication (WGD) model predicts contig pairs. Not seen previously in mosquitoes, the WGD would have to be specific to the cell line or its ancestral strain. However, the WGD is not apparent in the C6/36 karyotypes and would not by itself predict the high intra-PC heterozygosity. Another model postulates genomic differences between the 2 lots of cells that were grown and sequenced separately but combined in the assembly. This model predicts that the short reads would map preferentially to 1 contig of each PC since the short reads were derived from 1 lot exclusively. However, this was not the case. This model also predicts that raw long reads, mapped to contigs for consensus polish, would segregate by lot, but this was not the case (not shown). We conclude that the assembly presents both alleles at most loci. The alleles may or may not be phased; i.e., contigs may not consistently derive from the same haplotype along their full lengths.

### Annotation reveals male-specific Nix, 2 null forms of Dicer, endogenous virus

The NCBI RefSeq annotation of contigs yielded 143 606 exons in 38 706 genes, of which 28 625 are protein coding; 6833 genes had variants, and there were 42 899 mRNA transcripts. Results are public [24]. The RefSeq protein-coding gene set is 63% larger than the 17 539 protein-coding models described with the *A. albopictus* Foshan assembly [10] and likely includes allelic forms of many genes.

To assess conservation of genes and gene order between *A. albopictus* Foshan and the cell line, LiftOver [25] analysis was applied to these genomes in both directions. Of 27 093 nonoverlapping genes tested in C6/36, only 2190 (8%) were lifted while 17 121 (63%) were split. Of 17 146 nonoverlapping genes tested in Foshan, only 3364 (20%) were lifted while 8009 (47%) were split (Table S16). This analysis did not reveal high levels of gene-to-gene correspondence between the Foshan and C6/36 assemblies. This analysis may have been limited by the high dissimilarity observed between Foshan and C6/36 contigs, which would occlude context-dependent gene matching, and by consecutive alignments that hopped between homologous contigs, which would confound the recognition of conserved gene order.

The *Nix* gene in *A. aegypti* was previously shown to be located at the male-determining locus and to be necessary and sufficient to determine maleness [26]. The *A. albopictus* gene KP765684 (protein AKI28880), predicted from a partial coding sequence (CDS) generated by transcript assembly, was established as a homolog of *Nix* based on its sequence similarity, male-specificity, and transcription profile [26]. In the C6/36 assembly, KP765684 showed 100% nucleotide identity to a fragment in contig MNAF02001502 (NW_01 785 7498), a 970 929-bp contig that had ×17 short-read coverage indicative of allelic separation. An additional exon was identified in the C6/36 contig, and it is separated by a 107-bp intron from the previously known exon that encodes KP765684. The newly predicted gene sequence (LOC109397226) encodes a 282-aa NIX protein (XP_01 952 5102.1) that showed 70% similarity to the 288-aa *A. aegypti* NIX over the entire protein span. The position of the predicted intron is conserved with *A. aegypti*. Therefore, the discovery of the second exon in the C6/36 assembly extends the partial *A. albopictus Nix* gene KP765684 and further supports the homology of the *Nix* gene in the 2 species. The predicted protein is named “polyadenylate-binding protein 4-like protein” in the RefSeq annotation. There was no evidence of expression of this gene in our RNA sequence data from C6/36 cells at rest.

To assess the male-specificity of contig MNAF02001502, DNA sequence reads from male and female mosquitoes were mapped to the C6/36 assembly. The method of chromosomal quotient analysis [26–29] was applied to 1-Kbp spans of repeat-masked sequence. Using CQ = [(female alignments)/(male alignments)] [28] and a threshold of CQ <0.01 to indicate male specificity, fewer than 0.7% of 1-Kbp spans across the entire genome met the threshold while 14% of the 1-Kbp spans from contig MNAF02001502 did. It should be noted that the majority of the 1-Kbp spans were fully masked by repeats and did not report a CQ value. This result is again consistent with *Nix* and its contig MNAF02001502 being within the M-locus. It thus appears that the C6/36 cells are derived from 1 or more male mosquitoes and that they retain a full-length ortholog of NIX. These results suggest that the cell line could be used to study the molecular and biochemical pathways of sex determination, including the mechanism of NIX function.

**Figure 3:**
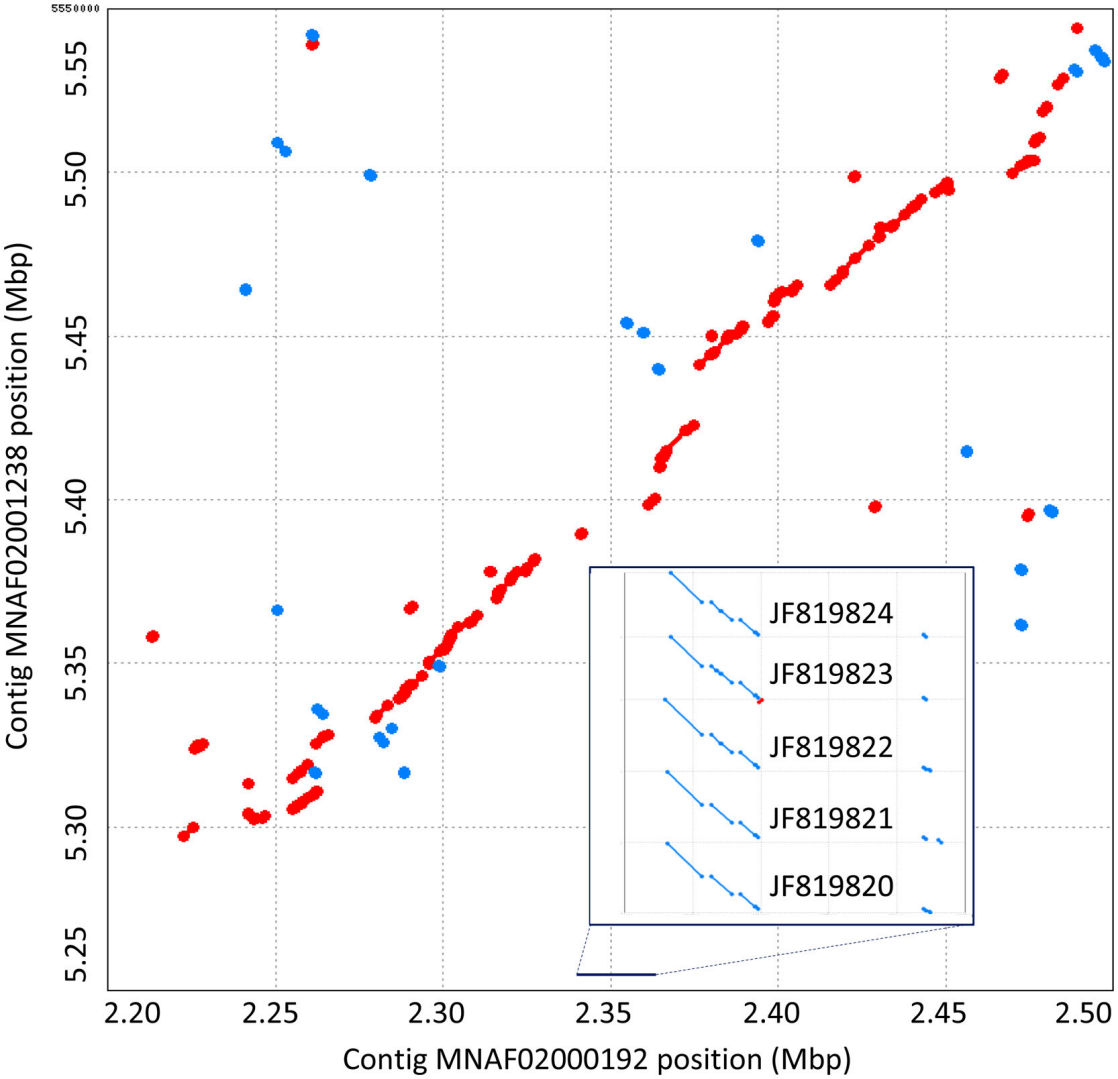
The C6/36 Dicer locus harbors a pseudogene allelic to a gene deletion. The dot plot illustrates 30-Kbp regions of contigs 192 and 1238. Red and blue dots indicate forward and reverse strand local alignments, respectively. These contigs were identified as paired contigs based on sharing of mapped short reads, indicating putative capture of alternate haplotypes. Five previously published *A. albopictus* Dicer 2 transcripts (inset) have full-length alignments to contig 192. These alignments span a single-base deletion, apparent in the contig sequence, corresponding to a previously reported null mutation in C6/36 cells. The same transcripts have only short alignments at their 5’ termini to contig 1238, indicating a previously unrecognized gene loss in the cognate allele. Additional images of the region are offered in Fig. S3.

C6/36 has been observed to have a functional *dcr-1* pathway [30] and a functional apoptosis pathway [31], but a dysfunctional antiviral RNA-interference response [32]. Previous genotyping of Dicer-like amplicons indicated a homozygous 1-bp deletion, causing a frameshift and a premature stop codon in the C6/36 *dcr-2* gene [33]. As the Dicer-mediated RNAi pathway has been implicated in host defense against virus in *Aedes aegypti* mosquitoes [34], the *dcr-2* null mutation suggests a mechanism for virus permissiveness in C6/36. Previously reported *dcr-2* transcript sequences align to both contigs of a PC, but all the transcripts have full-length alignments to 1 contig (MNAF02000192, i.e., NW_01 785 6188.1) and a small, fragmentary alignment to the other (MNAF02001238.1, i.e., scaffold NW_01 785 7234.1) (Fig. S3A and B). At the same position as the full-length alignments, C6/36 contig MNAF02000192 was annotated as endonuclease Dicer, LOC 109 403 945, with a 1-bp deletion relative to other strains (Fig. S3C and D). Both contigs, and both regions, have ∼×19 short-read coverage. Both regions have ample support by aligned long reads (Fig. S3E and F). Aligned to each other, the contigs show agreement on either side of the *dcr-2* locus but not within it (Fig. 3; Fig. S3G and H). These results confirm the previously reported frameshift mutation in C6/36 *dcr-2* but indicate a deletion of most of the gene in the cognate allele. The heterozygosity at this locus may have escaped notice due to a lack of matching primer sequences within the cognate allele.

Endogenous flavivirus sequences have been previously reported in C6/36 DNA [35]. Our ×20 genomic short reads were mapped to the 3290-bp “Aedes albopictus containing putative integrated non-retroviral sequence” from GenBank (accession AY223844.1). The resulting coverage depth ranged from ×20 to ×8171 (Fig. S4), indicating that portions of the sequence are present in the genome at high copy. The full-length viral sequence was mapped to the C6/36 assembly and found within the 5.5-Mbp contig MNAF02001791 (NW_01 785 7787), which has ×18 short-read coverage. Partial matches were found at 36 476 assembly locations in 1541 contigs. A small number (7663) of RNAseq reads from C6/36 cells at rest aligned to the virus sequence, indicating low-level transcription. Used as a control, sequence searches of the C6/36 genome assembly did not find the densovirus C6/36 DNV, which was discovered in chronically infected C6/36 cells and appears distinct from the host genome [36].

### Transcriptomics indicates low levels of aquaporins and Kir channels

The C6/36 RefSeq transcripts were predicted using public *A. albopictus* RNAseq, excluding our cell line RNAseq. The RefSeq transcripts were tested for presence or absence of expression in C6/36 cells using a single RNAseq run of cells at rest. Reads were mapped to transcript sequences, retaining at most 1 mapping per read, and RPKM was computed per transcript isoform without consolidation by gene. There were 14 483 transcripts with detectable expression (RPKM ≥ 1); the mean RPKM value of these transcripts was 32 (range = 1–5822). There were 1310 highly expressed transcripts based on a threshold of RPKM ≥64, i.e., having 2-fold or higher RPKM than the mean.

The highly expressed transcripts (HETs) were manually examined for the presence of genes belonging to 2 broad functional groups previously examined in the analysis of the *Aedes albopictus* Foshan genome [10]: detoxification proteins and odorant-binding proteins/receptors. Related to detoxification, 26 HETs were identified. Of these, 10 were cytochrome P450 oxidases (CYP450s), 2 were glutathione S-transferases (GSTs), 12 were ABC transporters, and 2 were carboxyl/cholinesterases (CCEs). Related to odorant binding proteins/receptors, 2 HETs were identified. These were orthologs of OBP9 in *An. gambiae* (AGAP000278) and OBP21 in *Ae. aegypti* (AAEL005770) (see Supplemental File “Detox_OBP.”

HETs were subjected to a DAVID analysis (v6.7) [37,38] to identify putative functional pathways enriched among the HETs. Among the HETs, DAVID identified 22 functional clusters that were significantly enriched (enrichment score > 1.3). By a process of manual categorization applied to mosquitoes previously [39], the enriched functional clusters were grouped into 7 broad themes: transcription and translation (8 clusters), protein sorting and trafficking (1 cluster), proteolysis (3 clusters), ATP metabolism (3 clusters), cytoskeletal functions (2 clusters), cell signaling (2 clusters), and generic (3 clusters) (see Table 2 for summary and the Supplementary file “DAVID” for details).

**Table 2:** Enrichment scores for functional clusters among the highest-expressed transcripts in C6/36 cells at rest.

Category	Functional cluster	Score
Transcription and translation		
	Ribosome	28.65
	Translation factor activity	9.98
	Protein folding	6.35
	rRNA binding	2.26
	Elongation factor	2.18
	Regulation of translation	1.63
	Heat shock protein 70	1.58
	RNA recognition motif	1.47
Protein sorting and trafficking		
	Protein transport	2.39
Proteolysis		
	Proteasome	1.62
	Ubiquitin	1.47
	Ubiquitin-mediated proteolysis	1.31
ATP metabolism		
	Oxidative phosphorylation	2.69
	Glycolysis	2.15
	Cytochrome-c oxidase activity	1.70
Cytoskeleton		
	Regulation of cytoskeleton organization	2.09
	Actin binding	1.39
Cell signaling		
	GTP binding	3.94
	*Rho*	1.73
Generic		
	Cellular homeostasis	2.52
	Nucleotide binding	2.15
	Proteasome component region	1.46

Functional clusters were generated and scored by DAVID and categorized manually. The “generic” functional clusters contain transcripts with no specific or consistent functional theme.

This analysis suggests that the C6/36 cell line is enriched with the molecular pathways for the (1) proper expression of mRNAs and proteins, (2) post-translational processing and trafficking of synthesized proteins, (3) protein turnover, and (4) synthesis of ATP. These would be expected of most cells. Similar results have been found in the transcriptome of the Malpighian tubules of non-blood-fed *A. albopictus* [39]. Intriguingly, the molecular pathways for (5) cytoskeletal function (e.g., cell division) and (6) cell signaling (e.g., responding to environmental cues) were enriched in the C6/36 cell line, but not in the Malpighian tubules of *A. albopictus*. This observation leads us to hypothesize that these enriched pathways may be cell line specializations for growth in laboratory cultures. Further studies are required to test this hypothesis.

Aquaporins (AQPs) are a family of transmembrane proteins that mediate the transport of H_2_O, small solutes (e.g., urea, glycerol), and gasses (e.g., CO_2_) across plasma membranes. Inward rectifier K^+^ (Kir) channels are a subfamily of K^+^ channels that mediate movements of K^+^ across plasma membranes. Recent work in mosquitoes has demonstrated that AQPs play key roles in water balance, heat tolerance, and vector competence [40–45], while Kir channels play key roles in renal transepithelial K^+^ and fluid secretion and fecundity [46–50]. Kir channels are also emerging targets for mosquitocide development [46–48,51]. Typically, the genomes of mosquitoes possess 6 genes encoding aquaporins: Drip (AQP1), Prip (AQP2), Bib (AQP3), Eglp1 (AQP4), Eglp2 (AQP5), and Aqp12L (AQP6); and at least 4 discrete genes encoding Kir channels: Kir1, Kir2A, Kir2B, and Kir3. There is some gene duplication in the C6/36 assembly, for which the 25 annotated AQP protein isoforms can be assigned to 15 haploid alleles from 9 (possibly 8) diploid loci, based on contig coordinates and PC relationships. Likewise, the 15 annotated Kir protein isoforms indicate some gene duplication of the 4 genes expected. Despite the robust number of AQP and Kir genes in C6/36 cells, AQP mRNA expression was not detected via RNA sequencing (i.e., RPKM < 1), and only 2 isoforms of Kir1 were nominally expressed (i.e., RPKM = 1), suggesting that the cell line may be limited in AQP-mediated water, solute, and gas transport, and Kir-mediated K^+^ transport (see the Supplementary file “AQP_Kir”). Our findings, derived from analysis of a single RNAseq run as described above, warrant further study.

### Demonstration of subtraction database for viral assays

Host subtraction is the bioinformatics process of filtering reads derived from host DNA and RNA, thereby enriching nonhost reads [52]. Host subtraction assists the discovery and characterization of viral sequences present at low titer in voluminous short-read data sets [52]. To evaluate utility for host subtraction in viral assays, the C6/36 transcript and genome sequences were used to filter RNA sequence reads from 26 samples of Zika-infected and 6 samples of mock-infected C6/36 cells. Quantitative polymerase chain reaction confirmed that Zika RNA was abundant in the Zika-infected samples vs the mock-infected samples. After this control measure, the remainder of the experiment emulated a search for any virus in cells exposed to an uncharacterized sample. A single multiplex sequencing library was prepared by the low-cost SISPA method [53]. Unpaired Illumina RNA sequencing reads were filtered by mapping to C6/36 (Table S17). Transcript mapping removed 13.2%, and genome mapping further removed 76.1% of total reads. The remaining 10.7% of reads were given a taxon assignment by blastn best hit to the NCBI nonredundant nucleotide database. Of total reads, 1.10% received a taxon assignment, including 0.011% assigned to Zika and 0 to any other viral taxon, indicating that Zika was detected accurately by this assay, which was not Zika-specific. Critically, subtraction reduced cpu time to 15% of what would have been required to blast all reads. Subtraction using instead the *Ae. albopictus* Foshan sequences was slightly less effective, leaving 13.2% of reads instead of 10.7% to be characterized by blast.

## Discussion

The C6/36 genome assembly offers large contigs attributable to deep coverage by long-read sequencing. Longer than the contigs of any previously assembled mosquito genome, the large contigs offer not only complete gene sequences but also gene context including repetitive DNA. The contigs are not joined by scaffolds, and they are not mapped to chromosomes, though physical mapping technologies including Hi-C, Dovetail, and BioNano technologies (reviewed in [54]), would be able to make use of our contigs. The contigs are publicly available, with gene annotation provided by the NCBI Eukaryotic Genome Annotation Pipeline. C6/36 joins CHO [83,84,85] and HeLa [55] as another cell line to have its genome *de novo* assembled. The accuracy of the assembly is supported by several observations. Short-read data that had not been used during assembly aligned to the assembly at a high rate with high identity. Alternate assemblies of the long reads, including those generated using different software, were very similar to those shown by local alignment and short-read mapping.

Most of the assembly contains duplicated sequence, though our karyotype analysis did not indicate a whole-genome duplication. We demonstrated that the duplication captures haplotype variants of the diploid genome. The duplication complicates analysis by gene count, but it makes the assembly a valuable reference for read mapping and for detection of allelism. Some recent genome projects intentionally separated homologous sequences during assembly [17,56], but the separation within the C6/36 assembly was a byproduct of heterozygosity within the genome. With additional resources, it might be possible to identify haplotype-phased blocks within contigs or to organize contigs into haplotype-phased scaffolds (e.g., [57]).

If the C6/36 assembly were complete and fully haplotype-separated, the total span would be twice the genome size. Compared with the *Aedes albopictus* Foshan mosquito assembly [10], the C6/36 contig span is only 28% larger. Some degree of haplotype separation may be present in the Foshan assembly, and the 2 assemblies may represent different strains or genomes of different size. Alignments with at least 95% identity covered small portions of both assemblies.

In the C6/36 assembly, we discovered a 2-exon sequence for *Nix*, the male-specific gene in *Aedes albopictus*. We confirmed the maleness of the cell line through differential mapping of reads from male and female mosquitoes as well as by the identification of the M factor *Nix*. This finding could be helpful for testing sex-specific agents developed for mosquito sterility programs. We also discovered that the cell line's *dcr-2* locus, source of the Dicer homolog in mosquitoes, contains a second null mutation allelic to the previously described truncated form.

Using RNA sequencing of cells at rest and the RefSeq annotation of the C6/36 assembly, we noted the conspicuous absence of mRNAs encoding AQPs and Kir channels. Our analysis of AQPs and Kir channels suggested that the cell line is limited in AQP-mediated water, solute, and gas transport, as well as Kir-mediated K^+^ transport. Further physiological studies will be required to confirm that the cell line indeed possesses weak functional activity of these channels. However, it is intriguing to speculate that the nominal AQP and Kir mRNA expression is an adaptation of the cell line to stable cell culture conditions wherein the extracellular environment, i.e., culture media, is not subject to fluctuations in osmolality, K^+^, or temperature, as would be experienced on a regular basis in the mosquito. Given that Kir channels in *Drosophila melanogaster* have been implicated in the RNA interference antiviral immune pathway [58], it is also possible that the nominal Kir mRNA expression contributes to the susceptibility of C6/36 cells to arboviral infection. It is unlikely that the original mosquito cells that generated the cell line would be deficient in AQP and Kir mRNA expression given the near ubiquitous expression of at least 1 AQP and Kir mRNA in various mosquito tissues that have been previously examined [39–45,50,59–62]. The lack of endogenous AQP and Kir mRNA expression may be serendipitous as it suggests that C6/36 cells have the potential to offer a mosquito-based cell line for functionally characterizing mosquito AQPs and Kir channels if the cells can be transfected with and induced to express exogenous cDNAs. The genome sequence should enable more extensive transcriptomics, including of cells at various stages of viral infection.

## Potential Implications

Our results should enable further use of the C6/36 cell line for virus detection, virus surveillance, vaccine and antiviral drug development, and promoting a basic understanding of the virus-host interplay for medically important mosquito-transmitted viruses. The genome sequence can be used as a bioinformatics filter to remove host sequence and thereby enrich nonhost reads among DNA or RNA sequence data from exposed cells. Using the assembly as a filter could avoid uncertainty that would be caused by the endogenous viral sequences whose presence we confirmed in the genome and the transcriptome. Filtering with a largely complete genome sequence will ease detection of novel and low-titer viruses. The genome sequence may enable discovery of microRNAs expressed by the cells in response to specific conditions. The genome sequence and annotation will enable reference-guided expression studies that characterize and quantify viral progression and host response. Applications for the cell line could expand if transcriptome studies revealed active pathways that could be targeted by insecticides or inactive pathways that could be studied by ectopic expression of insect genes.

## Methods

### Sequencing

Cells were obtained from 2 independent shipments of *Aedes albopictus* clone C6/36, ATCC CRL-1660 (ATCC Cat# CRL-1660, RRID:CVCL_Z230) from ATCC (Manassas, VA, USA). Cells were maintained in Minimal Essential Media supplemented with 10% fetal bovine serum and nonessential amino acids. Cells were maintained at 28°C and 5% CO_2_, and confluent monolayers were harvested by cell scraping. Following thaw, cells were passaged a single time and subjected to genomic DNA isolation (Qiagen, Germantown, MD, USA). DNA for PacBio sequencing was subjected to library construction following manufacturer instructions [63]. DNA from lot No. 59 479 117 was sequenced at the National Biodefense Analysis and Countermeasures Center (NBACC; Fort Detrick, MD, USA) using 128 SMRT cells and multiple libraries. DNA from lot No. 62 871 143 was sequenced at Icahn School of Medicine at Mt. Sinai (NY) using 80 SMRT cells. (Two formerly independent sequencing projects combined resources to generate 1 high-coverage assembly.) All sequencing used PacBio RS II instruments with P6C4 chemistry. Raw reads were extracted as subread FASTQ files from instrument h5 files using SMRTlink software. Lot No. 62 871 143 was also used for Illumina sequencing. Genomic DNA was Blue Pippin (Sage Science, Beverly, MA, USA) sheared to generate 270-bp fragments. These were end-repaired, A-tailed, and ligated to Illumina adaptors following standard protocols (NEB, Ipswich, MA, USA). Libraries were subjected to Illumina NextSeq 2 × 150-bp paired-end (PE) sequencing. Total RNA from C6/36 cells at rest was isolated with RNAeasy (Qiagen). Total RNA from cells at rest was enriched for messenger RNA with oligo dT dynabeads (Invitrogen, Carlsbad, CA, USA). Total RNA from mock-infected and Zika-infected samples was subjected to SISPA multiplex library construction [53] and sequenced by Illumina NextSeq 1 × 150-bp sequencing.

### Genome assembly

All Falcon [17] assemblies were generated on the DNAnexus (San Francisco, CA, USA) platform using the DNAnexus Falcon 0.0.1 application, which combined Falcon 0.4.2 with REPMask and TANMask from DAMASKER [64]. Raw reads of 10 731 bases or longer were subject to error correction. Corrected reads of 10 000 bases or more were subject to overlap and contig computation. The Falcon p_contig and a_contig sets were combined for analysis. All Canu (Canu, RRID:SCR_015880) [19] assemblies were generated with Canu [65] with the command “canu errorRate = 0.013 -p asm -d C636_canu genomeSize = 2g”; Falcon and Canu contigs were subject to consensus polishing using all the raw PacBio reads and either 1 or 2 iterations of Quiver or Arrow from PacBio SMRTlink. Falcon polishes used SMRTlink 3.1 on the DNAnexus platform. Canu polishes used SMRTlink 3.1.1 on an SGE grid [66]. The N50 statistic shows the length of the shortest contig such that contigs of equal or greater length span at least half of some total, usually the assembly size. The NG50 uses the putative genome size G, and for our NG50 calculations, G was set to the total span of the contigs from Canu and 2 rounds of Arrow (2 247 306 400 bp). Two contigs, each composed of a homopolymer repeat, were removed from the final assembly. With the exception of 30 contigs representing 1% of assembled bases, all contigs contained reads from both samples.

### Repeats, maps, alignments

Repeats were detected with the Repeat Modeler package (RepeatModeler, RRID:SCR_015027) [67–71], version 1.0.8. Short-read K-mer analysis was generated with GenomeScope [72], version 1.0, with k = 21. Short reads were mapped to assemblies with bowtie2, version 2.2.5 [73]. Mappings were restricted to concordantly mapped pairs under default parameters corresponding to end-to-end alignment and sensitive settings for 1 best mapping per pair with ties broken randomly, with the following exceptions. C6/36 genomic short-reads were mapped to the Foshan assembly in “very sensitive” mode. For detection of paired contigs, short reads were mapped to C6/36 contigs with the “best 4” parameter. The mapping of RNA reads to virus sequence was filtered for MapQ ≥5. Repeats were mapped to C6/36 contigs in local sensitive mode, retaining all alignments. Outputs were analyzed in bam format with samtools (SAMTOOLS, RRID:SCR_002105) and bedtools (BEDTools, RRID:SCR_006646) [74,75]. Mode contig coverage is computed as the center value of a 3-wide window starting with 3-fold coverage (3X) having the most bases in that coverage window; e.g., the minimum coverage window, 3X–5X, is reported as 4X. Contig local alignments were generated with nucmer 3.1, part of the MUMmer package [76] compiled for 64-bit processors and filtered with delta-filter –1, minimum 1000 bp. Local alignment dot plots were visualized with mummerplot. Global alignments were generated with ATAC [77], which computes maximal chains from 1-to-1 K-mers. Aligned spans were accumulated over stringent and permissive chains, denoted “M r” and “M c,” respectively, in the outputs.

### Gene annotation and analysis

Single-copy genes were downloaded from BUSCO v1.22 arthropoda-odp9 (BUSCO, RRID:SCR_015008) [23]. *Nix* was mapped with BLAST (NCBI BLAST, RRID:SCR_004870) [78]. The contig sequence was annotated by the NCBI Eukaryotic Genome Annotation Pipeline 7.2 [79]. Evidence included alignments of 5.6 G public *Aedes albopictus* RNA sequences (excluding C6/36 RNA sequence generated by this project) and 137 K public insect protein sequences. The annotation pipeline was adjusted to accommodate long introns containing transposable element (TE)- associated open reading frames (ORFs) as in *Aedes aegypti* [13], after such introns were detected in an initial run on C6/36. Mapping of gene annotations between assemblies was performed with LiftOver [25], which uses chained BLAT (BLAT, RRID:SCR_011919) [80] alignments to transfer coordinates. Chromosomal quotient analysis to identify male-specific contigs [26,28] used C6/36 contigs repeat masked [67] and split into 1 Kbp spans; reads were mapped by bowtie (Bowtie, RRID:SCR_005476) [81] with parameters –a –v 0. Pathway enrichment analysis was performed using DAVID v6.7 (DAVID, RRID:SCR_001881) [37,38] and best blastp hits in *An. gambiae* or *Ae. aegypti*.

### Transcriptome analysis

Mosquito proteins were taken from VectorBase release VB-2016–12. Best blast hits were found with NCBI blastall 2.2.26 [78] using default parameters and tabular outputs. Genes related to diapause, etc., were taken from supplemental documents SD9, SD13, and SD15 [10] and translated from *Aedes aegypti* Liverpool (AAEL) accession to the best blastp hit in C6/36.

### Growing cells for karyotype

Cells were grown in DMEM (Gibco, 11 995 065) supplemented with nonessential amino acids (Gibco, 11 140 050) and L-glutamine (Gibco, 25 030 081) at 28°C supplemented with 5% CO_2_. Once cells were 80% confluent, approximately 10^5^ cells were passaged into a 6-well plate by scraping. Once cells had settled and reattached to the plate, fresh C6/36 media was supplemented with Colcemid (ThermoFisher, 15 212 012) so that the final concentration was 1 μg/ml. After a 2-hour incubation at 28°C with 5% CO_2_, media was aspirated and cells rinsed with phosphate buffered saline (PBS) (Gibco, 14 040 133). Cells were scraped and resuspended in PBS.

### Fixing cells for karyotype

Cells suspended in PBS were centrifuged at 1000 rpm for 4 minutes and the supernatant was aspirated, leaving about 200 μl in the tube. The bottom of the tube was tapped to dislodge any clumps and 5 ml of ice cold 0.56% KCl solution was added, inverted once. The cells were incubated at room temperature for 6 minutes and centrifuged at 1000 rpm for 4 minutes. The supernatant was aspirated, leaving 50 μl in the tube. The pellet was resuspended by gently tapping the bottom of tube. The cells were fixed by adding 5 ml of methanol: glacial acetic acid (3:1) fixative solution. The fixative was added one or two drops at a time for the first 4 ml and tapping the bottom of the tube was performed to mix cells continuously. The fixed cells were centrifuged at 1000 rpm for 4 minutes, the supernatant was aspirated, and the cell pellet was resuspended in 200 ml of fixative.

### Visualizing chromosomes for karyotype

The fixed cells’ suspension (approx. 10–50 μl) was put on an alcohol-cleaned slide and air-dried for 1 hour. Then 10 μl of 30-nM DAPI (Invitrogen, D1306) solution in PBS was added to each slide. The slide was coverslipped and incubated in the dark at room temperature for more than 20 minutes. The coverslip was removed, and the slide was rinsed thoroughly with PBS. The slides were visualized on a Axioskop 2 (Zeiss, Oberkochen, Germany) plus fluorescence microscope using a DAPI filter, under oil immersion at ×1000 magnification. Images were taken with an AxioCam MRc5 (Zeiss) camera using AxioVision software. The color images were converted to grayscale, inverted, cropped, and adjusted for brightness and contrast in Photoshop (Adobe Systems, San Jose, CA, USA).

## Supporting Data

The assembly of the genome sequence is available at NCBI under accession GCA_0 018 76365.2. The contig accessions start at MNAF02000000; equivalent RefSeq accessions are listed in Table S18. The sequencing reads used to generate, validate, and analyze the assembly are available at NCBI SRA (see Tables S1 and S2 for accessions). The annotation describing the transcripts and genes is available at NCBI under *Aedes albopictus* Annotation Release 101, and the assembly and annotation are also available at VectorBase under the name canu_80X_arrow2.2. Additional supporting data are also available from the *GigaScience* repository, *Giga*DB [82].

### Abbreviations

AQP: aquaporin gene; ATCC: American Type Culture Collection, provider of C6/36 cells; BUSCO: Benchmarking Universal Single-Copy Orthologs, analysis of genes thought to be unique in Eukaryotic genomes; C6/36: a cell line derived from *Aedes albopictus*; Canu: A genome assembler derived from Celera Assembler; CQ: the Chromosomal Quotient used to measure male-specificity; HET: a highly expressed transcript; K-mer: here, a string of consecutive nucleotides with a specific length, K; PacBio: Pacific Biosystems, its sequencing platform, or the read type it generates; N50: the length of the shortest member in the smallest set of contigs required to span 50% of the assembly size; PC: as defined here, paired contigs that contain similar sequences; RPKM: RNA read count normalized by reads per kilobase of transcript per million reads.

### Competing interests

The author(s) declare that they have no competing interests.

### Funding

JCVI staff was supported by Department of Homeland Security (DHS) contract HSHQDC-15-C-B0059. X.G.C. was supported by the National Nature Science Foundation of China (81 420 108 024) and the Natural Science Foundation of Guangdong Province (2014A030312016). Z.T. was supported by National Institute of Allergy and Infectious Diseases (NIAIDP) grant AI123338. P.M.P. was supported by state and federal funds appropriated to the Ohio Agricultural Research and Development Center (OARDC) of the Ohio State University. S.K. and A.M.P. were supported by the Intramural Research Program of the National Human Genome Research Institute, National Institutes of Health. F.T.N. was supported by the Intramural Research Program of the National Institutes of Health, National Library of Medicine. N.H.B. and T.S. were supported under contract No. HSHQDC-07-C-00020 awarded by the DHS Science and Technology Directorate (S&T) for the management and operation of the NBACC, a Federally Funded Research and Development Center. The views and conclusions contained in this document are those of the authors and should not be interpreted as representing the official policies, either expressed or implied, of the DHS or S&T. In no event shall the DHS, NBACC, S&T, or Battelle National Biodefense Institute have any responsibility or liability for any use, misuse, inability to use, or reliance upon the information contained herein. DHS does not endorse any products or commercial services mentioned in this publication.

### Author contributions

Manuscript: J.R.M., P.M.P., R.S.S., Z.T., I.V.S., M.V.S. Sample prep: K.D. Karyotype: I.V.S., M.V.S., D.M.B., V.P. Sequencing: R.S. Assembly and sequence analysis: S.K., A.M.P., J.R.M., G.S. Repeats: D.H. LiftOver: B.R. Annotation: F.T.N. Nix: Z.T., X.G.C. Dicer: J.R.M. Flavivirus: R.S.S. Expression: P.M.P. Subtraction: J.R.M. Project conception: R.S., T.S., N.H.B., G.G.S., A.M.P., R.S.S.

## Supplementary Material

GIGA-D-17-00156_Original_Submission.pdf

GIGA-D-17-00156_Revision_1.pdf

Response_to_Reviewer_Comments_Original_Submission.doc

Reviewer_1_Report_(Original_Submission) -- Quan Nguyen 20 Aug 2017 Reviewed

Reviewer_1_Report_(Revision_1) -- Quan Nguyen 22 Dec 2017 Reviewed

Reviewer_2_Report_(Original_Submission) -- Monika Gulia-Nuss 12 Sep 2017 Reviewed

Supplemental material
