# Supplementary material for: Analysis of the Aedes albopictus C6/36 genome provides insight into cell line utility for viral propagation
Source: Gigascience. 2018 Jan 10;7(3):gix135. doi: 10.1093/gigascience/gix135 (PMC5869287; doi:10.1093/gigascience/gix135)
Supplement: GIGA-D-17-00156_Revision_1.pdf [file gix135_giga-d-17-00156_revision_1.pdf]

GigaScience

Analysis of the Aedes albopictus C6/36 genome provides insight into cell line utility for viral propagation

--Manuscript Draft--

**Manuscript Number:**	GIGA-D-17-00156R1
**Full Title:**	Analysis of the Aedes albopictus C6/36 genome provides insight into cell line utility for viral propagation
**Article Type:**	Research
**Funding Information:**	Intramural Research Program of the NIH National Library of Medicine — Dr Françoise Thibaud-Nissen<br>U.S. Department of Homeland Security (US) (HSHQDC-15-C-B0059) — Dr Reed S Shabman<br>National Nature Science Foundation of China (81420108024) — Dr Xiao-Guang Chen<br>Natural ScienceFoundation of Guangdong Province (2014A030312016) — Dr Xiao-Guang Chen<br>National Institute of Allergy and Infectious Diseases (AI123338) — Dr Zhijian Tu<br>Ohio Agricultural Research and Development Center, Ohio State University — Dr Peter M Piermarini<br>Intramural Research Program of the National Human Genome Research Institute — Dr Sergey Koren<br>Science and Technology Directorate (HSHQDC-07-C-00020) — Dr Nicholas H Bergman
**Abstract:**	Background: The 50-year old Aedes albopictus C6/36 cell line is a resource for the detection, amplification, and analysis of mosquito-borne viruses including Zika, dengue, and chikungunya. The cell line is derived from an unknown number of larvae from an unspecified strain of Aedes albopictus mosquitoes. Toward improved utility of the cell line for research in virus transmission, we present an annotated assembly of the C6/36 genome.<br>Results: The C6/36 genome assembly has the largest contig N50 (3.3 Mbp) of any mosquito assembly, presents the sequences of both haplotypes for most of the diploid genome, reveals independent null mutations in both alleles of the Dicer locus, and indicates a male-specific genome. Gene annotation was computed with publicly available mosquito transcript sequences. Gene expression data from cell line RNA sequence identified enrichment of growth-related pathways and conspicuous deficiency in aquaporins and inward rectifier K+ channels. As a test of utility, RNA sequence data from Zika-infected cells was mapped to the C6/36 genome and transcriptome assemblies. Host subtraction reduced the data set by 89%, enabling faster characterization of non-host reads.<br>Conclusions: The C6/36 genome sequence and annotation should enable additional uses of the cell line to study arbovirus vector interactions and interventions aimed at restricting the spread of human disease.
**Corresponding Author:**	Jason R. Miller, MS<br>J Craig Venter Institute<br>Rockville, MD UNITED STATES
**Corresponding Author Secondary Information:**	
**Corresponding Author's Institution:**	J Craig Venter Institute
**Corresponding Author's Secondary Institution:**	

Powered by Editorial Manager® and ProduXion Manager® from Aries Systems Corporation

**First Author:**	Jason R. Miller, MS
**First Author Secondary Information:**	
**Order of Authors:**	Jason R. Miller, MS
	Sergey Koren, PhD
	Kari A Dilley, PhD
	Vinita Puri
	David M Brown, PhD
	Derek M Harkins
	Françoise Thibaud-Nissen, PhD
	Benjamin Rosen, PhD
	Xiao-Guang Chen, MD, PhD
	Zhijian Tu, PhD
	Igor V Sharakhov, PhD
	Maria V Sharakhova, PhD
	Robert Sebra, PhD
	Timothy B Stockwell
	Nicholas H Bergman, PhD
	Granger G Sutton, PhD
	Adam M Phillippy, PhD
	Peter M Piermarini, PhD
	Reed S Shabman, PhD
**Order of Authors Secondary Information:**	
**Response to Reviewers:**	Authors' response to the Reviewers: We are grateful to the reviewers for their thoughtful feedback. We have implemented all of the Reviewer's suggestions. The revised manuscript is improved in very important ways. Our guide to changes is included here. Thank you.<br><br>Reviewer 1, Summary. In the manuscript, Miller and colleagues report their results on the genome assembly of the Aedes albopictus C6/36 cell line, an important laboratory cell line resource for studying mosquito-borne viruses. The author applied long-read PacBio DNA sequencing and Illumina NextSeq RNA sequencing to assemble and annotate the genome and transcriptome of the cell line. The analysis is appropriate and uses a range of computational programs for de novo assembly, repeat detection, genome annotation, RNA mapping, and cross-comparisons to a previous A. albopictus Foshan assembly. The results are valuable resources for future molecular studies of virus propagation and virus-mosquito vector interactions of several significant human-disease causing viruses, including Zika, Dengue, and Chikungunya viruses. I have several comments and suggestions below:<br><br>Reviewer 1, Point 1. The title "...insight into cell line adaptation..." needs to be revised, as the results presented are not sufficient to prove the adaptation of the current cell line compared to the original parental cell line for virus propagation. Direct comparisons to the parental cell line and better links to virus propagation are needed to prove the adaptation process.<br><br>Response:<br>This is a valid point and we agree. We have changed the title to remove the reference to adaptation: Analysis of the Aedes albopictus C6/36 genome provides insight into cell line utility for viral propagation.

Reviewer 1, Point 2. A limitation of the PacBio long read sequencing is the high sequencing error rate, and potential readers would be interested in the accuracy of the assembly. The current manuscript presents many results on the contig length and the coverage, but there is little discussion on the accuracy. The authors should discuss this aspect. The authors may consider moving one or more of the 17 supplementary tables into the main text to highlight their key findings. Likewise, Figure S3 may be considered moving into the main text.

Response:
It was an oversight to omit consensus accuracy analysis. In our initial submission, the lone indicator of consensus quality was present in Supplemental Table S4 which showed mismatch rate = 0.70 in mapped Illumina reads. We have now included new analysis in the main text by adding a paragraph to the end the Sequencing and Assembly section of Results. Unfortunately, we lack references with which to derive the average QV per assembled base. Instead, we present statistics showing a high level of agreement with the short read sequencing which was not used during assembly: The mapped reads covered 98.98% of assembled bases and confirmed 99.30% of aligned bases; 74.66% of mapped reads aligned end-to-end with zero mismatches and indels. We agree that some readers may be surprised to see high consensus accuracy in an assembly of high-error reads. To address this, we added a reference to our prior study of consensus quality in Canu assemblies in which consensus quality surpassed QV=30 (99.9% accuracy) on several data sets: These results are consistent with prior analyses, e.g. 99.98% identity to the Drosophila melanogaster reference achieved by a Canu+Quiver assembly of 90X P5C3 PacBio [19].

The Reviewer suggested including some of our supplemental tables or figures in the main text. We have acted on the Reviewer's specific suggestion to move Supplemental Figure S3, to the main text. The text now includes a new Figure 3 formed from a montage of two figures from Figure S3, which remains in the supplement unchanged. Figure 3, which is about the Dicer locus, covers one of our important gene findings and simultaneously illustrates the haplotype separated nature of our assembly.

Reviewer 1, Point 3. Table 2 shows weak results on the enrichment of the two broad categories: cytoskeletal functions and cell signaling. The suggestion that these two categories can be cell line specialisations for growth in the laboratory culture should be raised with caution. DAVID analysis based on 1,310 highly expressed transcripts (with selected thresholds >=2x the RPKM means and enrichment score >1.3) and manual categorisation of 22 functional clusters are not rigorous statistical analysis. Selection of highly expressed genes may lead to a biased analysis of abundant structural genes (cytoskeleton genes), metabolic genes, and house-keeping genes such as those for ribosomal genes (the top most enriched). More stringent gene-ontology enrichment or network analysis (with appropriate background genes, more specific sub/child ontologies, and possible comparisons with other cell lines as base lines) are needed to support the conclusion on the enrichment of the two broad categories (cytoskeletal function and cell signaling).

Response:
We thank the reviewer for this comment. We agree that a more comprehensive analysis is desirable. We plan to pursue future studies focused on the transcriptomics of this cell line. Given the limited nature of our transcriptomics data, our preference is to maintain our current analysis, which allows to compare and contrast with a previously published study by our group on Ae. albopictus Malpighian tubules. Nevertheless, we have softened our language when describing and interpreting the DAVID analysis to emphasize the preliminary nature of these results and need to confirm in future studies, and we added the conclusion: Further studies are required to test this hypothesis.

Reviewer 1, Point 4. The analysis and evidence to support the conclusion that the cell line is deficient in aquaporins and inward rectifier K+ channel need to be described with more details. Was the analysis based on the expression values (RPKM) measured from a single RNA sequencing sample? Were multiple mapped reads to AQP and Kir genes removed by the mapping and transcript counting pipelines (This may lead to low RPKM for these genes)? More biological/technical replicates may confirm whether the

genes were not expressed or lowly expressed. Comparing with data from other studies on the expression of AQP and Kir genes relative to other more abundant genes may help to support the conclusion.

Response:
The reviewer requests additional details on the experiment that was done and suggests additional comparisons that should be done.
We agree that additional data would be helpful but we regret that we are not able to provide it due to funding constraints. Instead, we have softened the statement on the deficiency of AQPs and Kir channels, which we agree had too strong of a conclusion. The transcriptomics data certainly suggest weak expression of these mRNAs, but we now emphasize that additional functional studies are required to confirm weak physiological activity of these channels.
The requested details were possibly already present in the manuscript. The text at the start of the transcriptomics results section says transcripts were tested for presence or absence based on a single run of RNAseq and that RPKM was computed per transcript without consolidation per gene. We have revised our manuscript so that readers will be referred to these methods descriptions. Specifically, we added this sentence at the end of the transcriptomics results section: Our findings, derived from analysis of a single RNAseq run as described above, warrant further study. Also, we added the detail that the mapping retained at most one mapping per read, which was previously mentioned only in the methods section.

Reviewer 1, Point 5. The implications of a subtraction database for viral detection is interesting. The authors may add discussion on how the multiplex sequencing method with the use of the subtraction database outperforms the quantitative PCR method in cost, sensitivity, and time.

Response:
If the question is whether a specific virus is present, the qPCR method would be preferable by cost, sensitivity, and time. The subtraction method is preferable if the question is what viruses, if any, are present. The subtraction method could be considered a filter for a metagenomics analysis. To clarify this point for readers, we have added text to the section on subtraction results. The new text emphasizes that qPCR was included as a control here but it would not be part of an actual subtraction experiment: After this control measure, the remainder of the experiment emulated a search for any virus in cells exposed to an uncharacterized sample.

Reviewer 1, Point 6. Figure 2 axis labels should be included in the figure, not in the legend.

Response:
Figure 2 has been revised. Thank you for this suggestion, which we also applied to the new Figure 3.

Reviewer 2, Summary. Cell lines are critical platforms for understanding biology of an organism; however, over the time the cell lines drift from the genome. In order to achieve meaningful results, it is important to know the cell and tissue types the cell lines were originally derived from. The manuscript sheds light on two important considerations in the use of a cell line for understanding mosquito-virus interaction:
1. It has drifted from the Ae. albopictus genome and shows lack of aquaporin transcripts which is important to know if a researcher is interested in these genes.
2. The cell line is potentially derived from male larvae and therefore might not be suitable for testing female specific gene pathways. As the authors' suggested this finding can potentially be used for testing sex-specific agents developed for mosquito sterility.

Reviewer 2, Point 1. This is important work and worthy of publication. However, I am not fully convinced that the library prep and sequencing at two different institutions (DNA from lot # 59479117 was sequenced at NBACC (Fort Detrick, MD) using 128 SMRT cells and multiple libraries. DNA from lot #62871143 was sequenced at Icahn School of Medicine at Mt. Sinai (NY) using 80 SMRT cells) will provide data for direct comparison. The differences in sample handling and library prep will result in

	variations; however, I am of the opinion that it will not change the authors' conclusion. The authors still need to explain why the sequencing was carried out at two different sites.<br><br>Response:<br>The C6/36 assembly is derived from two lots of cells, both labeled C6/36 and provided by ATCC. Each was sequenced at a different lab but both used the PacBio RS II instrument with P6C4 chemistry and the manufacturer's recommended prep. The reason for the separate origins of cells is that our experiment represents a merger of two formerly independent projects. Both projects had similar designs and both were underway when the project leaders discovered the redundancy during discussions at a conference. Our groups decided to pool resources in order to generate a high-quality assembly that would exploit roughly twice the sequencing depth as either project could provide separately. We hope the community benefits from our collaboration.<br>We added the following historical and parenthetical remark to the description of sequencing in the Methods section: (Two formerly independent sequencing projects combined resources to generate one high-coverage assembly.)<br>We also added a sentence to assuage concerns that the two lots may have assembled separately. We added this sentence to the Methods section near the text that contained the description of the separate lots: With the exception of 30 contigs representing 1% of assembled bases, all contigs contained reads from both samples.<br><br>Other changes:<br>The original draft included the claim that C6/36 was the second cell line to have its genome de novo assembled. One of us since found three publications on the CHO cell line. We revised the Discussion section to cite the publications of CHO and HeLa genomes. The revised text makes no claim to rank order: C6/36 joins CHO [83,84,85] and HeLa [55] as another cell line to have its genome de novo assembled. The new references were added to the end of the current references to make clear which references are new. We will renumber all references prior to publication.<br>We revised the supplemental material to reflect an update to RefSeq that occurred after our prior manuscript submission. The RefSeq annotation of the C6/36 Dicer locus formerly described a putatively transcribed gene with the note that the contig consensus sequence was missing one base. The revised RefSeq annotation accepts the consensus and describes a pseudogene. Therefore, we revised the caption to Supplemental Figure S3c-d to include the sentence: We have updated the RefSeq annotation to note a "polymorphic pseudogene" at LOC109403945.<br>We inserted the word "RNAseq" where it could add clarity to descriptions of our mapped RNA sequencing data.<br>We added accessions where it could help users find the data at NCBI. We inserted RefSeq accessions with prefixes "LOC", "XP", and "NW" at various places in the main text. We added Supplemental Table S18 that lists the GenBank/MNAF and RefSeq/NW accessions for each contig.<br>We revised the caption to Figure 1, which is about karyotypes, to improve the clarity of the text.<br>We updated the affiliations for several authors.
**Additional Information:**	
**Question**	**Response**
Are you submitting this manuscript to a special series or article collection?	No
**Experimental design and statistics**<br><br><br>Full details of the experimental design and statistical methods used should be given in the Methods section, as detailed in our Minimum Standards Reporting Checklist. Information essential to interpreting the data presented should be made available in the figure legends.	Yes

Powered by Editorial Manager® and ProduXion Manager® from Aries Systems Corporation

Have you included all the information requested in your manuscript?	
**Resources**<br><br>A description of all resources used, including antibodies, cell lines, animals and software tools, with enough information to allow them to be uniquely identified, should be included in the Methods section. Authors are strongly encouraged to cite Research Resource Identifiers (RRIDs) for antibodies, model organisms and tools, where possible.<br><br>Have you included the information requested as detailed in our Minimum Standards Reporting Checklist?	Yes
**Availability of data and materials**<br><br>All datasets and code on which the conclusions of the paper rely must be either included in your submission or deposited in publicly available repositories (where available and ethically appropriate), referencing such data using a unique identifier in the references and in the “Availability of Data and Materials” section of your manuscript.<br><br>Have you have met the above requirement as detailed in our Minimum Standards Reporting Checklist?	Yes

Manuscript Click here to download Manuscript C636.Manuscript.docx

TITLE PAGE

Title: Analysis of the *Aedes albopictus* C6/36 genome provides insight into cell line utility for viral propagation

Corresponding Author: Jason Miller.
Authors (first / last / email / affiliation):

Jason R	Miller	jmill02@shepherd.edu	1,2*
Sergey	Koren	sergey.koren@nih.gov	3
Kari A	Dilley	karidilley@gmail.com	1
Vinita	Puri	vpuri@jcvi.org	1
David M	Brown	dbrown@jcvi.org	1
Derek M	Harkins	dharkins@jcvi.org	1
Françoise	Thibaud-Nissen	thibaudf@ncbi.nlm.nih.gov	4
Benjamin	Rosen	Ben.Rosen@ars.usda.gov	5
Xiao-Guang	Chen	xgchen2001@hotmail.com	6
Zhijian	Tu	jaketu@vt.edu	7
Igor V	Sharakhov	igor@vt.edu	8,9
Maria V	Sharakhova	msharakh@vt.edu	8,9
Robert	Sebra	robert.sebra@mssm.edu	10
Timothy B	Stockwell	Timothy.Stockwell@nbacc.dhs.gov	11
Nicholas H	Bergman	Nicholas.Bergman@nbacc.dhs.gov	11
Granger G	Sutton	GSutton@jcvi.org	1
Adam M	Phillippy	adam.phillippy@nih.gov	3
Peter M	Piermarini	piermarini.1@osu.edu	12
Reed S	Shabman	rshabman@atcc.org	13

Affiliations: ***** Corresponding author, ORCID: 0000-0002-6912-2925. **1** J. Craig Venter Institute, 9714 Medical Center Drive, Rockville MD 20850. **2** College of Natural Sciences and Mathematics, Shepherd University, Shepherdstown WV 25443. **3** Genome Informatics Section, Computational and Statistical Genomics Branch, National Human Genome Research Institute, Bethesda, MD 20892.**3** NCBI/NLM/NIH, 45 Center Drive, Bethesda, MD USA 20894. **5** USDA 10300 Baltimore Ave., Bldg 306 Barc-East, Beltsville MD 20705-2350. **6** Department of Pathogen Biology, School of Public Health and Tropical Medicine, Southern Medical University, Guangzhou 510515, China. **7** Department of Biochemistry and the Fralin Life Science Institute,Virginia Tech, Blacksburg, VA. **8** Department of Entomology and the Fralin Life Science Institute, Virginia Tech, Blacksburg, VA. **9** Laboratory of Ecology, Genetics and Environmental Protection, Tomsk State University, Tomsk, Russia. **10** Icahn

School of Medicine at Mount Sinai, New York NY 10029. **11** NBACC, Fort Detrick, MD 21702. **12** Department of Entomology, The Ohio State University, Ohio Agricultural Research and Development Center, Wooster, OH 44691. **13** ATCC, 217 Perry Parkway, Gaithersburg Maryland 20877.

ABSTRACT

Background: The 50-year old *Aedes albopictus* C6/36 cell line is a resource for the detection, amplification, and analysis of mosquito-borne viruses including Zika, dengue, and chikungunya. The cell line is derived from an unknown number of larvae from an unspecified strain of *Aedes albopictus* mosquitoes. Toward improved utility of the cell line for research in virus transmission, we present an annotated assembly of the C6/36 genome.

Results: The C6/36 genome assembly has the largest contig N50 (3.3 Mbp) of any mosquito assembly, presents the sequences of both haplotypes for most of the diploid genome, reveals independent null mutations in both alleles of the Dicer locus, and indicates a male-specific genome. Gene annotation was computed with publicly available mosquito transcript sequences. Gene expression data from cell line RNA sequence identified enrichment of growth-related pathways and conspicuous deficiency in aquaporins and inward rectifier K^+ channels. As a test of utility, RNA sequence data from Zika-infected cells was mapped to the C6/36 genome and transcriptome assemblies. Host subtraction reduced the data set by 89%, enabling faster characterization of non-host reads.

Conclusions: The C6/36 genome sequence and annotation should enable additional uses of the cell line to study arbovirus vector interactions and interventions aimed at restricting the spread of human disease.

KEYWORDS

Aedes albopictus C6/36 cell line genome assembly

BACKGROUND

Insect cell lines such as Aag2 and C6/36 are critical platforms for insect biology and virology. The *Aedes albopictus* clone C6/36 (ATCC CRL-1660) cell line is commonly used for detection, propagation, and analysis of arboviruses, including antibody-based detection of viruses in saliva [reviewed in [1]]. C6/36 cells have a short population doubling time and are permissive to infection by mosquito-transmitted viruses across members of the *Togaviridae*, *Flaviviridae*, and *Bunyaviridae* families. In particular, C6/36 cells are used to study viruses that pose significant threats to human health, including Zika, dengue, chikungunya. Virus propagation in C6/36 cells guides the rational development of vaccines and therapeutics. PubMed [2] lists 671 publications with C6/36 in the title or abstract.

The progenitor of the C6/36 cell line was established in 1967 from freshly hatched *Aedes albopictus* larvae of unspecified ancestry [3]. The C6/36 subclone was selected for its uniformly high virus yield and was shown to retain a diploid karyotype with 2n=6 chromosomes in a majority of cells [4]. The similar or equivalent ATC-15 cells [1] were shown to be diploid [5] and to have more chromosomal abnormalities after 110 passages than after 17 [6]. The C6/36 cell line, available through the American Type Culture Collection (ATCC, Manassas VA) is described as maintaining a diploid chromosome number and being non-anchorage dependent and non-tumorigenic [7]. Despite the widespread use of this cell line to both propagate arboviruses and to use them as a tool to study virus-mosquito interactions, little has been published about features that differentiate the cell line genome from that of *Aedes albopictus* mosquitoes.

Two strains of *A. albopictus* have published genomes, both of which were sequenced on Illumina platforms and assembled with the SOAPdenovo assembler [8]. Sequencing of the

Italian Fellini aka Rimini strain yielded small contigs with N50 < 1 Kbp [9]. The assembly of a Foshan female from China [10] as provided in VectorBase [11] version AaloF1, has a 1.92 Gbp scaffold span, 1.78 Gbp contig span, and 18.4 Kbp contig N50. A third strain was analyzed for its genomic repeats using a pipeline called dnaPipeTE that runs on Illumina reads [12]. The *A. aegypti* Liverpool genome was assembled to draft status from Sanger reads [13] and later de-duplicated (removing putative redundant contigs) and extended to chromosome-length scaffolds with Hi-C technology [14]. The 2014 update in VectorBase has an 82 Kbp contig N50. Using these assemblies, the within-genus divergence between *A. albopictus* and *A. aegypti* was estimated at 71.4 mya [10]. High population heterozygosity has been recognized in mosquitoes for over 35 years [15], indicating the C6/36 cells could harbor a heterozygous genome.

Recent advances in DNA sequencing technology have enabled the generation of megabase-scale contigs. The Pacific BioSciences (PacBio, Menlo Park CA) and Oxford Nanopore (Oxford UK) single-molecule sequencing platforms can generate reads in excess of 10 Kbp. Due to its randomness, the high base call error in PacBio reads can be overcome by using sequencing depths in the 50X range [16]. New assembly algorithms targeting deep-coverage PacBio data have separated the haplotypes from heterozygous regions of diploid genomes [17].

Here we describe findings for the *Aedes albopictus* C6/36 cell line including its karyotype, the assembly of its genome from PacBio sequence, an analysis of the haplotype separation in contigs, the gene annotation based on public mosquito RNA sequence, and analysis of gene expression based on cell line RNA. We also demonstrate use of the genome and transcriptome for the purpose of subtracting host sequence during an RNA sequencing

assay for viruses. The sequence data were previously deposited in public databases to facilitate research on Zika and other viruses commonly transmitted by *Aedes albopictus* and studied in C6/36 cell lines.

DATA DESCRIPTION

The assembly of the genome sequence is available at NCBI [18] under accession GCA_001876365.2. The contig accessions start at MNAF02000000; equivalent RefSeq accessions are listed in **Table S18**. The annotation describing the transcripts and genes is available at NCBI under *Aedes albopictus* Annotation Release 101. The sequencing reads used to generate, validate, and analyze the assembly are available at NCBI SRA; see **Table S1 and S2** for accessions. The assembly and annotation are also available at VectorBase [11] under the name canu_80X_arrow2.2.

ANALYSIS

C6/36 cells, obtained from ATCC (CRL-1660) and cultured at JCVI, were subjected to visual analysis of stained metaphase chromosomes to ascertain the karyotype. All cells examined displayed the three metacentric chromosomes expected of mosquito cells. A majority of cells also displayed additional chromosomes, as shown in **Figure 1**, and the specific composition varied per cell. This analysis suggested variable and cell-specific partial duplications of chromosomes. Notably, a whole-genome duplication was not indicated.

Figure 1. Three karyotypes of the C6/36 *Ae. albopictus* cell line. Chromosomes are labeled chr1for the shortest, chr2 for the longest, and chr3 for the intermediate size chromosomes within each image. 1a: Cell has three normal paired chromosomes. An additional acrocentric chromosome pair has a short arm indicating deletion or translocation to elsewhere. An additional

short haploid chromosome is unpaired. 1b: Cell has chromosomes with pairs slightly separated. Chr1 appears normal. The other chromosomes are abnormal, possibly due to translocation of most of one arm of chr2 to one arm of chr3. 1c: Cell with chromosome pairs separated. Chr1, chr2, and chr3 appear normal. The chr1 homologous pairs overlap. There are several additional short metacentric and acrocentric chromosomes shown by arrows. The 5 μm scale bar applies to all three images.

Sequencing and assembly generate large contigs

Genomic long-read sequencing for assembly generated 161 Gbp in 17.9M total reads providing 147 Gbp in 12.4 M reads 5 Kbp or longer and 107 Gbp in 7.10 M reads 10 Kbp or longer. See **Table S1** for accessions. Genomic short-read sequencing for analysis generated 45.8 Gbp in 152 M pairs of 2x150bp reads. Transcript sequencing yielded 16.6 Gbp in 27.7 M pairs of 2x300bp partially overlapping reads. See **Table S2** for accessions.

Ten candidate assemblies were generated by combinations of four software packages: either the Falcon or the Canu assembler [17, 19], followed by zero to two iterations of either the Quiver or Arrow consensus polisher [20]. As shown in **Table S3**, the ten resulting assemblies had similar size profiles. The sum of bases was 2.25 Gbp in all assemblies. The average long-read coverage was 72X. Each assembly mapped 93% of the 20X paired short reads, which had not been used during assembly; see **Table S4**. Local alignments aligned the assemblies' entire spans in large segments. For example, the contigs of Falcon and Canu after 2 rounds of Arrow each, had 2.24 Gbp in alignments of at least 99% sequence identity, and these aligned spans had 630 Kbp N50.

The assembly chosen for downstream analysis was the one from Canu plus two rounds of Arrow because its 93.2% short-read map rate was highest by a small margin. This assembly had a total span of 2.247 Gbp in 2,434 contigs and a contig N50 of 3.304 Mbp; see **Table S5**. This assembly is available in GenBank with accession GCA_001876365.2. To our knowledge, the

C6/36 contig N50 exceeds that of any other assembly of any mosquito or mosquito cell line genome, though some other assemblies offer scaffolds and chromosome mappings in addition to contigs; see **Table S6**.

Accuracy of the C6/36 consensus was inferred from the mapping of short reads, which were not used to generate the assembly. The mapped reads covered 98.98% of assembled bases and confirmed 99.30% of aligned bases; 74.66% of mapped reads aligned end-to-end with zero mismatches and indels. These results are consistent with prior analyses, *e.g.* 99.98% identity to the *Drosophila melanogaster* reference achieved by a Canu+Quiver assembly of 90X P5C3 PacBio [19].

Dissimilarity with other mosquito assemblies

The C6/36 assembly was compared to *Aedes albopictus* Foshan [10]. The C6/36 contig span is 28% larger. Global alignments spanned 816 Mbp (stringent) or 1.44 Gbp (permissive) of both assemblies; see **Table S7**. Local alignments covered 692 Mbp of Foshan contigs and 1,028 Mbp of C6/36 contigs; see **Table S8**. Thus, both alignment methods left large portions of both assemblies unaligned. Sequence identity within alignments was low. The local alignments with at least 95% sequence identity covered only 373 Mbp of Foshan and 596 Mbp of C6/36. Local alignments covered more of C6/36 than Foshan, indicating that some sequences are present at higher multiplicity in the C6/36 assembly. To explore the Foshan vs. C6/36 genome difference free of the C6/36 assembly, the C6/36 short read pairs were mapped to Foshan contigs. As shown in **Table S9**, 93% of pairs had mapped to C6/36 contigs but only 49% mapped to Foshan contigs. Of pairs not mapped to C6/36, less than 1% mapped to Foshan. These results combine to indicate dissimilarity of the Foshan and C6/36 genomes. It is possible that the cell line is derived from an

A. albopictus strain that was itself diverged from Foshan. Inter-strain genome size differences had been noted in this species prior to the sequencing era [21].

Inter-species nucleotide alignment was unproductive. Permissive global alignments to *Aedes aegypti* [13, 14] covered only 15% (290 Mbp) of Foshan and 14% (304 Mbp) of C6/36.

C6/36 repeats were detected, characterized, and mapped back to the assembly with the process used for Foshan [10]. As shown in **Table S10**, results were similar to those reported for Foshan. Repeats cover 74% of C6/36 (and 76% of Foshan) and the three most abundant repeat types accounted for 59% of assembled bases (and 60% of Foshan bases). The most abundant repeat types were Unknown, LINE retrotransposon, and DNA transposon in C6/36 (and LINE, LTR retrotransposon, and Other in Foshan).

Redundancy indicates haplotype separation

The Canu assembler can separate haplotype regions having over 2% divergence [19]. Therefore, we evaluated the C6/36 assembly for haplotype separation. The first evaluation used the genomic short reads, which were not used by the assembler. K-mer analysis of the short reads, independent of the assembly, estimated 5.7% heterozygosity within the C6/36 genome and a genome size less than half the assembled contig span; see **Figure S1a**. Mapping the reads to contigs yielded an overall coverage mode of 18X; see **Figure S1b**. The coverage mode for most individual contigs was also about 18X; see **Figure 2** and **Table S11**. The bimodal coverage is similar to that seen in the *Quercus lobata* (oak) genome assembly, Figure 2B in [22], for which the assembler putatively separated the haplotypes at divergent loci. In C6/36, the 1832 contigs whose mode coverage was in the 18X ± 6X range collectively span 95.82% of the assembled bases. There is a smaller group of large contigs with mode coverage in the 36X ± 6X range.

These results support the hypothesis that most of the C6/36 diploid genome is represented twice in the assembly, possibly due to haplotype separation at heterozygous loci, such that collapsed sequences from less heterozygous loci attracted twice as much short-read coverage.

Figure 2. Contig size vs coverage. Each bar in the scatter plot represents one contig to which short reads were mapped. The 1832 contigs with mode coverage in the 18X ± 6X range collectively span 95.82% of the assembled bases, but there are a few large contigs with 36X ± 6X coverage. The apparent bi-modal distribution suggests that the ~18X contigs could contain separate representations of heterozygous loci, while the ~36X contigs could contain the consensus of both alleles from less heterozygous loci.

Second, the C6/36 assembly was tested for the presence of sequence-similar contigs. After alignment of the nucleotide sequence to itself was inconclusive, short reads were mapped allowing up to 4 maps per pair, and the 15% of read pairs that mapped exactly twice were used to identify paired contigs (PCs), defined here as two contigs sharing at least 10,000 read pairs that mapped twice. This identified the 529 PCs described in **Table S12**. There were 708 contigs in PCs (474 contigs in exactly one PC and maximum 8 PCs for one contig). There were 689 PC contigs with short-read coverage modes in the 18X ± 6X range (maximum coverage was 51X). The PCs incorporated 1.97 Gbp or 88% of the 2.25 Gbp assembly. Sequence similarity within PCs was low; average 93.5% identity in aligned bases, with 28% of the sequence aligned (274 Mbp in alignments covering 548 Mbp on contigs). Where PC alignments left one contig extending past the other, it was possible to “walk” from PC to PC as illustrated in **Figure S2**. One walk, involving 9 PCs and 9 contigs, all with 18X ± 6X coverage, spanned about 18 Mbp total; see **Figure S2d.** These walks point to duplicated sequences, longer than individual contigs, that are present twice with low similarity, with each copy represented within a different group of

adjoining contigs. Since PCs incorporate most of the assembly, the duplication most likely represents spans from homologous chromosomes that were assembled to into separate contigs.

As a third evaluation, the C6/36 contigs were subjected to BUSCO analysis [23] using genes thought to have single-copy orthologs across arthropods. Of 2624 genes found in contigs, 64% appeared as two instances. As shown in **Table 1**, 99.6% of two-instance genes involved 18X ± 6X contigs. There was a significant association of genes having 2 instances in the assembly and having all instances on 18X ± 6X contigs (Fisher's exact test, $p<0.01$, **Table S14**). Thus, the gene duplication mirrors the redundancy observed in PCs.

Number of Instances	Genes Found	Genes on contigs with 18X	Genes on contigs with 36X	Genes only on contigs with 18X	Genes only on contigs with 36X	Instances on contigs with 18X	Instances on contigs with 36X	Instances on other contigs
1	825	704	113	704	113	704	113	8
2	1,668	1,662	57	1,600	2	3,262	59	15
3	98	98	9	80	0	275	10	9
4	24	24	1	23	0	94	2	0
5 to 8	9	9	0	9	0	57	0	0
Total	2,624	2,497	180	2,416	115	4,392	184	32

Table 1. BUSCO gene analysis. BUSCO genes are presumed single-copy in eukaryotic genomes but most occur twice in the C6/36 assembly. BUSCO arthropod genes were searched against C6/36 contigs. Two instances were found for 1668 genes. The genes were further evaluated for whether any of their instances occurred contigs with short-read coverage in the 18X ± 6X or 36X ± 6X range. These coverage values suggest haplotype separation and collapse, respectively, within the contig sequences. Of genes with exactly two instances in the assembly, 1662 (99.6%) had at least one instance on a 18X-range contig, while only 57 had at least one instance on a 36X-range contig. This supports the characterization of 18X-range contigs as containing sequences specific to a haplotype. **Table S13** gives the coordinates and short-read coverage of every instance.

The situation appears nuanced for the 825 one-copy genes. Almost 14% of one-copy genes mapped to contigs with 36X ± 6X coverage, suggesting these genes are on diploid contigs represented by a consensus of two alleles. Another 1% (7 genes) hit contigs with very high (over

40X) coverage, suggesting these genes may be replicated in the genome but underrepresented in the assembly. Most single-copy genes, 85%, mapped to contigs with 18X ± 6X coverage, similar to the portion that mapped to PC contigs. Genes mapping to only one contig of a PC, suggesting a "missing gene", were inspected further. The mapped loci did not show elevated short-read coverage, as would be expected if the gene were higher copy in the genome than the assembly. The alignments did not involve contig ends, as would be expected if the cognate gene belonged in an assembly gap, or unusual levels of discontinuity. Some "missing genes" actually did have fragmentary alignments, suggesting gene loss. As illustrated in **Table S15**, PC#1, the PC with the most shared short reads, spans 17 BUSCO genes. Its two-copy genes are ordered consistently across 6 Mbp but these are interspersed by 4 genes found on only one or the other of the two contigs, plus one gene with additional copies elsewhere in the assembly. Thus, this PC presents syntenic sequences spanning structural variants and indicates that some single-copy BUSCO genes on 18X ± 6X contigs are attributable to allele-specific gene loss.

By the three methods of read mapping, contig alignment, and gene finding, we consistently found duplication within the assembly. The results are consistent with a model of a heterozygous genome for which spans from both haplotypes are represented in the assembly. This model predicts the genome size is 52% of the assembly size, or 1.172 Gbp. Alternate models have less support. A whole-genome duplication (WGD) model predicts contig pairs. Not seen previously in mosquitoes, the WGD would have to be specific to the cell line or its ancestral strain. However, the WGD is not apparent in the C6/36 karyotypes and would not by itself predict the high intra-PC heterozygosity. Another model postulates genomic differences between the two lots of cells that were grown and sequenced separately but combined in the assembly. This model predicts the short reads would map preferentially to one contig of each PC since the

short reads were derived from one lot exclusively. However, this was not the case. This model also predicts that raw long reads, mapped to contigs for consensus polish, would segregate by lot, but this was not the case (not shown). We conclude that the assembly presents both alleles at most loci. The alleles may or may not be phased, that is, contigs may not consistently derive from the same haplotype along their full lengths.

Annotation reveals male-specific Nix, two null forms of Dicer, endogenous virus

The NCBI RefSeq annotation of contigs yielded 143,606 exons in 38,706 genes of which 28,625 are protein coding. 6,833 genes had variants and there were 42,899 mRNA transcripts. Results are public [24]. The RefSeq protein-coding gene set is 63% larger than the 17,539 protein-coding models described with the *A. albopictus* Foshan assembly [10] and likely includes allelic forms of many genes.

To assess conservation of genes and gene order between *A. albopictus* Foshan and the cell line, LiftOver [25] analysis was applied to these genomes in both directions. Of 27,093 non-overlapping genes tested in C6/36, only 2,190 (8%) were lifted while 17,121 (63%) were split. Of 17,146 non-overlapping genes tested in Foshan, only 3,364 (20%) were lifted while 8,009 (47%) were split; **Table S16**. This analysis did not reveal high levels of gene-to-gene correspondence between the Foshan and C6/36 assemblies. This analysis may have been limited by the high dissimilarity observed between Foshan and C6/36 contigs, which would occlude context-dependent gene matching, and by consecutive alignments that hopped between homologous contigs, which would confound the recognition of conserved gene order.

The *Nix* gene in *A. aegypti* was previously shown to be located at the male-determining locus and to be necessary and sufficient to determine maleness [26]. The *A. albopictus* gene

KP765684 (protein AKI28880), predicted from a partial CDS generated by transcript assembly, was established as a homolog of *Nix* based on its sequence similarity, male-specificity and transcription profile [26]. In the C6/36 assembly, KP765684 showed 100% nucleotide identity to a fragment in contig MNAF02001502 (NW_017857498), a 970,929 bp contig that had 17X short-read coverage indicative of allelic separation. An additional exon was identified in the C6/36 contig and it is separated by a 107 bp intron from the previously known exon that encodes KP765684. The newly predicted gene sequence (LOC109397226) encodes a 282 aa NIX protein (XP_019525102.1) that showed 70% similarity to the 288 aa *A. aegypti* NIX over the entire protein span. The position of the predicted intron is conserved with *A. aegypti*. Therefore, the discovery of the second exon in the C6/36 assembly extends the partial *A. albopictus Nix* gene KP765684 and further supports the homology of the *Nix* gene in the two species. The predicted protein is named "polyadenylate-binding protein 4-like protein" in the RefSeq annotation. There was no evidence of expression of this gene in our RNA sequence data from C6/36 cells at rest.

To assess the male-specificity of contig MNAF02001502, DNA sequence reads from male and female mosquitoes were mapped to the C6/36 assembly. The method of chromosomal quotient analysis [26-29] was applied to 1 Kbp spans of repeat-masked sequence. Using CQ = [(female alignments) / (male alignments)] [28] and a threshold of CQ<0.01 to indicate male specificity, fewer than 0.7% of 1 Kbp spans across the entire genome met the threshold while 14% of the 1 Kbp spans from contig MNAF02001502 did. It should be noted that the majority of the 1 Kbp spans were fully masked by repeats and did not report a CQ value. This result is again consistent with *Nix* and its contig MNAF02001502 being within the M-locus. It thus appears that the C6/36 cells are derived from one or more male mosquitoes and that they retain a full-length

ortholog of NIX. These results suggest the cell line could be used to study the molecular and biochemical pathways of sex determination, including the mechanism of NIX function.

C6/36 has been observed to have a functional *dcr-1* pathway (see [30]) and a functional apoptosis pathway [31] but a dysfunctional antiviral RNA-interference response [32]. Previous genotyping of Dicer-like amplicons indicated a homozygous 1 bp deletion causing a frameshift and a premature stop codon in the C6/36 *dcr-2* gene [33]. Since the Dicer-mediated RNAi pathway has been implicated in host defense against virus in *Aedes aegypti* mosquitoes [34], the *dcr-2* null mutation suggests a mechanism for virus permissiveness in C6/36. Previously reported *dcr-2* transcript sequences align to both contigs of a PC but all the transcripts have full-length alignments to one contig (MNAF02000192 *i.e.* NW_017856188.1) and a small, fragmentary alignment to the other (MNAF02001238.1 *i.e.* scaffold NW_017857234.1); see **Figure S3a-b**. At the same position as the full-length alignments, C6/36 contig MNAF02000192 was annotated as endonuclease Dicer, LOC 109403945, with a 1 bp deletion relative to other strains; see **Figure S3c-d**. Both contigs, and both regions, have ~19X short-read coverage. Both regions have ample support by aligned long reads; see **Figure S3e-f**. Aligned to each other, the contigs show agreement on either side of the *dcr-2* locus but not within it; see **Figure 3** and **Figure S3g-h**. These results confirm the previously reported frameshift mutation in C6/36 *dcr-2* but indicate a deletion of most of the gene in the cognate allele. The heterozygosity at this locus may have escaped notice due to a lack of matching primer sequences within the cognate allele.

Figure 3. The C6/36 Dicer locus harbors a pseudogene allelic to a gene deletion. The dot plot illustrates 30 Kbp regions of contigs 192 and 1238. Red and blue dots indicate forward and reverse strand local alignments respectively. These contigs were identified as Paired Contigs based on sharing of mapped short reads, indicating putative capture of alternate haplotypes. Five previously published *A. albopictus* Dicer 2 transcripts (inset) have full-length alignments to contig 192. These alignments span a single-base deletion, apparent in the contig sequence,

corresponding to a previously reported null mutation in C6/36 cells. The same transcripts have only short alignments at their 5' termini to contig 1238, indicating a previously unrecognized gene loss in the cognate allele. Additional images of the region are offered in **Fig. S3**.

Endogenous flavivirus sequences have been previously reported in C6/36 DNA [35]. Our 20X genomic short reads were mapped to the 3290 bp "Aedes albopictus containing putative integrated non-retroviral sequence" from GenBank (accession AY223844.1). The resulting coverage depth ranged from 20X to 8171X (**Figure S4**) indicating that portions of the sequence are present in the genome at high copy. The full-length viral sequence was mapped to the C6/36 assembly and found within the 5.5 Mbp contig MNAF02001791 (NW_017857787), which has 18X short read coverage. Partial matches were found at 36,476 assembly locations in 1541 contigs. A small number (7663) of RNAseq reads from C6/36 cells at rest aligned to the virus sequence, indicating low-level transcription. Used as a control, sequence searches of the C6/36 genome assembly did not find the densovirus C6/36 DNV, which was discovered in chronically infected C6/36 cells and appears distinct from the host genome [36].

Transcriptomics indicates low levels of aquaporins and Kir channels

The C6/36 RefSeq transcripts were predicted using public *A. albopictus* RNAseq excluding our cell line RNAseq. The RefSeq transcripts were tested for presence or absence of expression in C6/36 cells using a single RNAseq run of cells at rest. Reads were mapped to transcript sequences, retaining at most one mapping per read, and RPKM was computed per transcript isoform without consolidation by gene. There were 14,483 transcripts with detectable expression (RPKM $\geq$ 1); the mean RPKM value of these transcripts was 32 (range = 1 to 5,822). There were 1,310 highly-expressed transcripts based on a threshold of RPKM $\geq$ 64, i.e. having 2-fold or higher RPKM than the mean.

The highly expressed transcripts (HETs) were manually examined for the presence of genes belonging to two broad functional groups previously examined in the analysis of the *Aedes albopictus* Foshan genome [10]: detoxification proteins and odorant-binding proteins/receptors. Related to detoxification, 26 HETs were identified. Of these, 10 were cytochrome P450 oxidases (CYP450s), 2 were glutathione S-transferases (GSTs), 12 were ABC transporters, and 2 were carboxyl/cholinesterases (CCEs). Related to odorant binding proteins/receptors, 2 HETs were identified. These were orthologs of OBP9 in *An. gambiae* (AGAP000278) and OBP21 in *Ae. aegypti* (AAEL005770). See **Supplemental File "Detox_OBP"**.

HETs were subjected to a DAVID analysis (v6.7) [37, 38] to identify putative functional pathways enriched among the HETs. Among the HETs, DAVID identified 22 functional clusters that were significantly enriched (enrichment score > 1.3). By a process of manual categorization applied to mosquitoes previously [39], the enriched functional clusters were grouped into 7 broad themes: transcription and translation (8 clusters); protein sorting and trafficking(1 cluster); proteolysis (3 clusters); ATP metabolism (3 clusters); cytoskeletal functions (2 clusters); cell signaling (2 clusters); and generic (3 clusters). See **Table 2** for summary and **Supplemental File "DAVID"** for details.

This analysis suggests the C6/36 cell line is enriched with the molecular pathways for the 1) proper expression of mRNAs and proteins, 2) post-translational processing and trafficking of synthesized proteins, 3) protein turnover, and 4) synthesis of ATP. These would be expected of most cells. Similar results had been found in the transcriptome of the Malpighian tubules of non-blood fed *A. albopictus* [39]. Intriguingly, the molecular pathways for 5) cytoskeletal function (e.g., cell division) and 6) cell signaling (e.g., responding to environmental cues) were enriched in the C6/36 cell line, but not in the Malpighian tubules of *A. albopictus*. This observation leads

us to hypothesize that these enriched pathways may be cell line specializations for growth in laboratory cultures. Further studies are required to test this hypothesis.

Aquaporins (AQPs) are a family of transmembrane proteins that mediate the transport of H_2O, small solutes (e.g., urea, glycerol), and gasses (e.g., CO_2) across plasma membranes. Inward rectifier K^+ (Kir) channels are a subfamily of K^+ channels that mediate movements of K^+ across plasma membranes. Recent work in mosquitoes has demonstrated that AQPs play key roles in water balance, heat tolerance, and vector competence, [40-45] while Kir channels play key roles in renal transepithelial K^+ and fluid secretion and fecundity [46-50]. Kir channels are also emerging targets for mosquitocide development [46-48, 51]. Typically, the genomes of mosquitoes possess 6 genes encoding aquaporins: Drip (AQP1), Prip (AQP2), Bib (AQP3), Eglp1 (AQP4), Eglp2 (AQP5), and Aqp12L (AQP6); and at least 4 discrete genes encoding Kir channels: Kir1, Kir2A, Kir2B, and Kir3. There is some gene duplication in the C6/36 assembly, for which the 25 annotated AQP protein isoforms can be assigned to 15 haploid alleles from 9 (possibly 8) diploid loci, based contig coordinates and PC relationships. Likewise, the 15 annotated Kir protein isoforms indicate some gene duplication of the 4 genes expected. Despite the robust number of AQP and Kir genes in C6/36 cells, AQP mRNA expression was not detected via RNA sequencing (*i.e.* RPKM < 1), and only two isoforms of Kir1 were nominally expressed (*i.e.* RPKM = 1), suggesting that the cell line may be limited in AQP-mediated water, solute, and gas transport, and Kir-mediated K^+ transport. See **Supplemental File "AQP_Kir".** Our findings, derived from analysis of a single RNAseq run as described above, warrant further study.

Category	Functional cluster	Score
Transcription and Translation		
	Ribosome	28.65

	Translation factor activity	9.98
	Protein folding	6.35
	rRNA binding	2.26
	Elongation factor	2.18
	Regulation of translation	1.63
	Heat shock protein 70	1.58
	RNA recognition motif	1.47
Protein Sorting and Trafficking		
	Protein transport	2.39
Proteolysis		
	Proteasome	1.62
	Ubiquitin	1.47
	Ubiquitin mediated proteolysis	1.31
ATP Metabolism		
	Oxidative phosphorylation	2.69
	Glycolysis	2.15
	Cytochrome-c oxidase activity	1.70
Cytoskeleton		
	Regulation of cytoskeleton organization	2.09
	Actin-binding	1.39
Cell Signaling		
	GTP-binding	3.94
	Rho	1.73
Generic		
	Cellular homeostasis	2.52
	Nucleotide binding	2.15
	Proteasome component region	1.46

Table 2. Enrichment scores for functional clusters among the highest-expressed transcripts in C6/36 cells at rest. Functional clusters were generated and scored by DAVID and categorized manually. The 'generic' functional clusters contains transcripts with no specific or consistent functional theme.

Demonstration of subtraction database for viral assays

Host subtraction is the bioinformatics process of filtering reads derived from host DNA and RNA, thereby enriching non-host reads [52]. Host subtraction assists the discovery and characterization of viral sequences present at low titer in voluminous short-read datasets [52]. To evaluate utility for host subtraction in viral assays, the C6/36 transcript and genome sequences

were used to filter RNA sequence reads from 26 samples of Zika-infected and 6 samples of mock-infected C6/36 cells. Quantitative PCR confirmed Zika RNA was abundant in the Zika-infected samples vs. the mock-infected samples. After this control measure, the remainder of the experiment emulated a search for any virus in cells exposed to an uncharacterized sample. A single multiplex sequencing library was prepared by the low-cost SISPA method [53]. Unpaired Illumina RNA sequencing reads were filtered by mapping to C6/36; see **Table S17**. Transcript mapping removed 13.2%, and genome mapping further removed 76.1%, of total reads. The remaining 10.7% of reads were given a taxon assignment by blastn best hit to the NCBI non-redundant nucleotide database. Of total reads, 1.10% received a taxon assignment including 0.011% assigned to Zika, and zero to any other viral taxon, indicating that Zika was detected accurately by this assay which was not Zika-specific. Critically, subtraction reduced cpu time to 15% of what would have been required to blast all reads. Subtraction using instead the *Ae. albopictus* Foshan sequences was slightly less effective leaving 13.2% of reads instead of 10.7% to be characterized by blast.

DISCUSSION

The C6/36 genome assembly offers large contigs attributable to deep coverage by long-read sequencing. Longer than the contigs of any previously assembled mosquito genome, the large contigs offer not only complete gene sequences but also gene context including repetitive DNA. The contigs are not joined by scaffolds and they are not mapped to chromosomes, though physical mapping technologies including Hi-C, Dovetail, and BioNano technologies (reviewed in [54]), would be able to make use of our contigs. The contigs are publically available with gene annotation provided by the NCBI Eukaryotic Genome Annotation Pipeline.

C6/36 joins CHO [83,84,85] and HeLa [55] as another cell line to have its genome *de novo* assembled. The accuracy of the assembly is supported by several observations. Short-read data that had not been used during assembly aligned to the assembly at a high rate with high identity. Alternate assemblies of the long reads, including those generated using different software, were very similar as shown by local alignment and short-read mapping.

Most of the assembly contains duplicated sequence though our karyotype analysis did not indicate a whole-genome duplication. We demonstrated that the duplication captures haplotype variants of the diploid genome. The duplication complicates analysis by gene count but it makes the assembly a valuable reference for read mapping and for detection of allelism. Some recent genome projects intentionally separated homologous sequences during assembly [17, 56] but the separation within the C6/36 assembly was a byproduct of heterozygosity within the genome. With additional resources, it might be possible to identify haplotype-phased blocks within contigs or to organize contigs into haplotype-phased scaffolds, *e.g.* [57].

If the C6/36 assembly were complete and fully haplotype-separated, the total span would be twice the genome size. Compared to the *Aedes albopictus* Foshan mosquito assembly [10], the C6/36 contig span is only 28% larger. Some degree of haplotype separation may be present in the Foshan assembly and the two assemblies may represent different strains or genomes of different size. Alignments with at least 95% identity covered small portions of both assemblies.

In the C6/36 assembly, we discovered a two-exon sequence for *Nix*, the male-specific gene in *Aedes albopictus*. We confirmed maleness of the cell line through differential mapping of reads from male and female mosquitoes as well as by the identification of the M factor *Nix*. This finding could be helpful for testing sex-specific agents developed for mosquito sterility

programs. We also discovered that the cell line's *dcr-2* locus, source of the Dicer homolog in mosquitoes, contains a second null mutation allelic to the previously described truncated form.

Using RNA sequencing of cells at rest, and the RefSeq annotation of the C6/36 assembly, we noted the conspicuous absence of mRNAs encoding AQPs and Kir channels. Our analysis of AQPs and Kir channels suggested the cell line is limited in AQP-mediated water, solute, and gas transport, as well as Kir-mediated K^+ transport. Further physiological studies will be required to confirm that the cell line indeed possesses weak functional activity of these channels. However, it is intriguing to speculate that the nominal AQP and Kir mRNA expression is an adaptation of the cell line to stable cell culture conditions wherein the extracellular environment, *i.e.* culture media, is not subject to fluctuations in osmolality, K^+, or temperature as would be experienced on a regular basis in the mosquito. Given that Kir channels in *Drosophila melanogaster* have been implicated in the RNA interference antiviral immune pathway [58], it is also possible that the nominal Kir mRNA expression contributes to the susceptibility of C6/36 cells to arboviral infection. It is unlikely that the original mosquito cells that generated the cell line would be deficient in AQP and Kir mRNA expression given the near ubiquitous expression of at least one AQP and Kir mRNA in various mosquito tissues that have been previously examined [39-45, 50, 59-62]. The lack of endogenous AQP and Kir mRNA expression may be serendipitous as it suggests that C6/36 cells have potential to offer a mosquito-based cell line for functionally-characterizing mosquito AQPs and Kir channels, if the cells can be transfected with and induced to express exogenous cDNAs. The genome sequence should enable more extensive transcriptomics including of cells at various stages of viral infection.

POTENTIAL IMPLICATIONS

Our results should enable further use of the C6/36 cell line for virus detection, virus surveillance, vaccine and antiviral drug development, and promoting a basic understanding of the virus-host interplay for medically important mosquito-transmitted viruses. The genome sequence can be used as a bioinformatics filter to remove host sequence and thereby enrich non-host reads among DNA or RNA sequence data from exposed cells. Using the assembly as a filter could avoid uncertainty that would be caused by the endogenous viral sequences whose presence we confirmed in the genome and the transcriptome. Filtering with a largely-complete genome sequence will ease detection of novel and low-titer viruses. The genome sequence may enable discovery of microRNAs expressed by the cells in response to specific conditions. The genome sequence and annotation will enable reference-guided expression studies that characterize and quantify viral progression and host response. Applications for the cell line could expand if transcriptome studies reveal active pathways that could be targeted by insecticides or inactive pathways that could be studied by ectopic expression of insect genes.

METHODS

Sequencing. Cells were obtained from two independent shipments of *Aedes albopictus* clone C6/36, ATCC CRL-1660 (ATCC Cat# CRL-1660, RRID:CVCL_Z230) from ATCC (Manassas VA). Cells were maintained in Minimal Essential Media supplemented with 10% fetal bovine serum and nonessential amino acids. Cells were maintained at 28°C and 5% CO_2, and confluent monolayers were harvested by cell scraping. Following thaw, cells were passaged a single time and subjected to genomic DNA isolation (Qiagen, Germantown MD). DNA for PacBio sequencing was subjected to library construction following manufacturer instructions [63]. DNA from lot # 59479117 was sequenced at NBACC (Fort Detrick, MD) using 128 SMRT

cells and multiple libraries. DNA from lot #62871143 was sequenced at Icahn School of Medicine at Mt. Sinai (NY) using 80 SMRT cells. (Two formerly independent sequencing projects combined resources to generate one high-coverage assembly.) All sequencing used PacBio RS II instruments with P6C4 chemistry. Raw reads were extracted as subread FASTQ files from instrument h5 files using SMRTlink software. Lot #62871143 was also used for Illumina sequencing. Genomic DNA was Blue Pippin (Sage Science, Beverly MA) sheared to generate 270 bp fragments. These were end-repaired, A-tailed, and ligated to Illumina adaptors following standard protocols (NEB, Ipswich MA). Libraries were subjected to Illumina NextSeq 2x150 bp paired end (PE) sequencing. Total RNA from C6/36 cells at rest was isolated with RNAeasy (Qiagen). Total RNA from cells at rest was enriched for messenger RNA with oligo dT dynabeads (Invitrogen, Carlsbad CA). Total RNA from mock-infected and Zika-infected samples were subjected to SISPA multiplex library construction [53] and sequenced by Illumina NextSeq 1x150 bp sequencing.

Genome assembly. All Falcon [17] assemblies were generated on the DNAnexus (San Francisco CA) platform using the DNAnexus Falcon 0.0.1 application which combined Falcon 0.4.2 with REPMask and TANMask from DAMASKER [64]. Raw reads of 10,731 bases or longer were subject to error correction. Corrected reads of 10,000 bases or more were subject to overlap and contig computation. The Falcon p_contig and a_contig sets were combined for analysis. All Canu (Canu, RRID:SCR_015880) [19] assemblies were generated with Canu [65] with the command 'canu errorRate=0.013 -p asm -d C636_canu genomeSize=2g'. Falcon and Canu contigs were subject to consensus polishing using all the raw PacBio reads and either one or two iterations of Quiver or Arrow from PacBio SMRTlink. Falcon polishes used SMRTlink 3.1 on the DNAnexus platform. Canu polishes used SMRTlink 3.1.1 on an SGE grid; see [66].

The N50 statistic shows the length of the shortest contig such that contigs of equal or greater length span at least half of some total, usually the assembly size. The NG50 uses the putative genome size, **G**, and for our NG50 calculations, **G** was set to the total span of the contigs from Canu and 2 rounds of Arrow (2,247,306,400 bp). Two contigs, each composed of a homopolymer repeat, were removed from the final assembly. With the exception of 30 contigs representing 1% of assembled bases, all contigs contained reads from both samples.

Repeats, maps, alignments. Repeats were detected with the Repeat Modeler package (RepeatModeler, RRID:SCR_015027)[67-71] version 1.0.8. Short-read K-mer analysis was generated with GenomeScope [72] version 1.0 with k=21. Short reads were mapped to assemblies with bowtie2 version 2.2.5 [73]. Mappings were restricted to concordantly mapped pairs under default parameters corresponding to end-to-end alignment and sensitive settings for one best mapping per pair with ties broken randomly, with the following exceptions. C6/36 genomic short-reads were mapped to the Foshan assembly in "very sensitive" mode. For detection of Paired Contigs (PCs), short reads were mapped to C6/36 contigs with the "best 4" parameter. The mapping of RNA reads to virus sequence was filtered for MapQ≥5. Repeats were mapped to C6/36 contigs in local sensitive mode retaining all alignments. Outputs were analyzed in bam format with samtools (SAMTOOLS , RRID:SCR_002105) and bedtools (BEDTools , RRID:SCR_006646)[74, 75]. Mode contig coverage is computed as the center value of a 3 wide window starting at 3 fold coverage (3X) having the most bases in that coverage window; for example, the minimum coverage window, 3-5X, is reported as 4X. Contig local alignments were generated with nucmer 3.1, part of the MUMmer package [76] compiled for 64-bit processors and filtered with delta-filter -1, minimum 1000 bp. Local alignment dot plots were visualized with mummerplot. Global alignments were generated with ATAC [77] which computes maximal

chains from 1-to-1 K-mers. Aligned spans were accumulated over stringent and permissive chains, denoted ‘M r’ and ‘M c’, respectively, in the outputs.

Gene annotation and analysis. Single-copy genes were downloaded from BUSCO v1.22 arthropoda-odp9 (BUSCO , RRID:SCR_015008)[23]. *Nix* was mapped with BLAST (NCBI BLAST , RRID:SCR_004870)[78]. The contig sequence was annotated by the NCBI Eukaryotic Genome Annotation Pipeline 7.2 [79]. Evidence included alignments of 5.6 G public *Aedes albopictus* RNA sequences (excluding C6/36 RNA sequence generated by this project) and 137 K public insect protein sequences. The annotation pipeline was adjusted to accommodate long introns containing TE-associated ORFs as in *Aedes aegypti* [13], after such introns were detected in an initial run on C6/36. Mapping of gene annotations between assemblies was performed with LiftOver [25] which uses chained BLAT (BLAT, RRID:SCR_011919)[80] alignments to transfer coordinates. Chromosomal quotient analysis to identify male-specific contigs [26, 28] used C6/36 contigs repeat masked [81] and split into 1 Kbp spans; reads were mapped by bowtie (Bowtie, RRID:SCR_005476)[82] with parameters –a –v 0. Pathway enrichment analysis was performed using DAVID v6.7 (DAVID, RRID:SCR_001881)[37, 38] and best blastp hits in *An. gambiae* or *Ae. aegypti*.

Transcriptome analysis. Mosquito proteins were taken from VectorBase release VB-2016-12. Best blast hits were found with NCBI blastall 2.2.26 [78] using default parameters and tabular outputs. Genes related to diapause etc. were taken from [10] supplemental documents SD9, SD13, SD15., and translated from *Aedes aegypti* Liverpool (AAEL) accession to the best blastp hit in C6/36.

Growing cells for karyotype. Cells were grown in DMEM (Gibco, 11995065) supplemented with non-essential amino acids (Gibco, 11140050) and L-glutamine (Gibco,

25030081) at 28°C supplemented with 5% CO_2. Once cells were 80% confluent, approximately 10^5 cells were passaged into a 6 well plate by scraping. Once cells had settled and reattached to the plate, fresh C6/36 media was supplemented with Colcemid (ThermoFisher, 15212012) so that the final concentration was (1 μg/ml). After a 2 h incubation at 28°C with 5% CO_2 media was aspirated and cells rinsed with PBS (Gibco, 14040133). Cells were scraped and resuspended in PBS.

Fixing cells for karyotype. The cells suspended in PBS were centrifuged at 1000 rpm for 4 minutes, aspirated supernatant, leaving about 200 μl in the tube. The bottom of the tube was tapped to break any clumps and added 5 ml of ice cold 0.56% KCl solution, inverted once. The cells were incubated at room temperature for 6 minutes and centrifuged at 1000 rpm for 4 minutes. The supernatant was aspirated, leaving 50 μl in the tube. The pellet was resuspended by gently tapping the bottom of tube. The cells were fixed by adding 5 ml of methanol: glacial acetic acid (3:1) fixative solution, the fixative was added one or two drops at a time for the first 4 ml and tapping the bottom of the tube to mix cells continuously. The fixed cells were centrifuged at 1000 rpm for 4 minutes, aspirated the supernatant and suspended the cells pellet in 200 μl of fixative.

Visualizing chromosomes for karyotype. The fixed cells suspension (approx. 10-50 μl) was put on an alcohol cleaned slide and air dried for 1 hour. Then 10 μl of 30 nM DAPI (Invitrogen, D1306) solution in PBS was added to each slide. The slide was coverslipped and incubated in dark at room temperature for greater than 20 minutes. The coverslip was removed and slide was rinsed thoroughly with PBS. The slides were visualized on a Axioskop 2 plus (Zeiss, Oberkochen, Germany) fluorescence microscope using DAPI filter, under oil immersion at 1000X magnification. Images were taken with AxioCam MRc5 (Zeiss) camera using

AxioVision software. The color images were converted to grayscale, inverted, cropped, and adjusted for brightness and contrast in Photoshop (Adobe Systems, San Jose CA).

Supporting Data

The assembly of the genome sequence is available at NCBI under accession GCA_001876365.2. The contig accessions start at MNAF02000000; equivalent RefSeq accessions are listed in **Table S18**. The sequencing reads used to generate, validate, and analyze the assembly are available at NCBI SRA; see **Table S1 and S2** for accessions. The annotation describing the transcripts and genes is available at NCBI under *Aedes albopictus* Annotation Release 101, and the assembly and annotation are also available at VectorBase under the name canu_80X_arrow2.2. Additional supporting data is also available from the *GigaScience* GigaDB repository[83].

DECLARATIONS

List of abbreviations

AQP: aquaporin gene. **ATCC**: American Type Culture Collection, provider of C6/36 cells. **BUSCO**: Benchmarking Universal Single-Copy Orthologs, analysis of genes thought to be unique in Eukaryotic genomes. **C6/36**: A cell line derived from *Aedes albopictus*. **Canu**: A genome assembler derived from Celera Assembler. **CQ**: the Chromosomal Quotient used to measure male-specificity. **HET**: a Highly Expressed Transcript. **K-mer**: here, a string of consecutive nucleotides with a specific length, K. **PacBio**: Pacific Biosystems, its sequencing platform, or the read type it generates. **N50**: the length of the shortest member in the smallest set of contigs required to span 50% of the assembly size. **PC**: as defined here, Paired Contigs that contain similar sequences. **RPKM**: RNA read count normalized by Reads Per Kilobase of transcript per Million reads.

Consent for publication

Not applicable.

Competing interests

The author(s) declare that they have no competing interests

Funding

JCVI staff was supported by DHS contract HSHQDC-15-C-B0059. XGC was supported by the National Nature Science Foundation of China (81420108024) and the Natural ScienceFoundation of Guangdong Province (2014A030312016). ZT was supported by NIAID grant AI123338. PMP was supported by state and federal funds appropriated to the OARDC of the Ohio State University. SK and AMP were supported by the Intramural Research Program of the National Human Genome Research Institute, National Institutes of Health. FTN was supported by the Intramural Research Program of the NIH, National Library of Medicine. NHB and TS were supported under Contract No. HSHQDC-07-C-00020 awarded by the Department of Homeland Security (DHS) Science and Technology Directorate (S&T) for the management and operation of the National Biodefense Analysis and Countermeasures Center (NBACC), a Federally Funded Research and Development Center. The views and conclusions contained in this document are those of the authors and should not be interpreted as necessarily representing the official policies, either expressed or implied, of the DHS or S&T. In no event shall the DHS, NBACC, S&T or Battelle National Biodefense Institute (BNBI) have any responsibility or liability for any use, misuse, inability to use, or reliance upon the information contained herein. DHS does not endorse any products or commercial services mentioned in this publication.

Authors' contributions

Manuscript: JRM, PMP, RSS, ZT, IVS, MVS. Sample prep: KD. Karyotype: IVS, MVS, DMB, VP. Sequencing: RS. Assembly & sequence analysis: SK, AMP, JRM, GS. Repeats: DH. Liftover: BR. Annotation: FTN. Nix: ZT, XGC. Dicer: JRM. Flavivirus: RSS. Expression: PMP. Subtraction: JRM. Project conception: RS, TS, NHB, GGS, AMP, RSS.

Acknowledgements

We are grateful for contributions from Chai Fungtammasan and Brett Hannigan at DNAnexus; Karen Beeri formerly at JCVI; Carlos J. Esquivel at OSU; Yang Wu at VT; Tracy Ferguson, Kisha Parker, and Wai Kwan Chung at NBACC.

REFERENCES

1. Walker, T., et al., *Mosquito cell lines: history, isolation, availability and application to assess the threat of arboviral transmission in the United Kingdom.* Parasit Vectors, 2014. **7**: p. 382.
2. Lu, Z., *PubMed and beyond: a survey of web tools for searching biomedical literature.* Database, 2011. **2011**: p. baq036.
3. Singh, K.R.P., *Cell cultures derived from larvae of Aedes albopictus (Skuse) and Aedes aegypti (L.).* Current Science, 1967. **36**(19): p. 506-508.
4. Igarashi, A., *Isolation of a Singh's Aedes albopictus cell clone sensitive to Dengue and Chikungunya viruses.* J Gen Virol, 1978. **40**(3): p. 531-44.
5. Chiplonkar, J.M., et al., *Karyological studies on established mosquito cell lines.* Experientia, 1983. **39**(6): p. 608-10.
6. Wagh, U.V., et al., *Studies on characterization of mosquito cell lines.* Cytometry, 1984. **5**(4): p. 403-7.
7. ATCC, *Product Sheet: Aedes albopictus clone C6/36 (ATCC® CRL-1660™)*. 2017.

8. Luo, R., et al., *SOAPdenovo2: an empirically improved memory-efficient short-read de novo assembler.* GigaScience, 2012. **1**(1): p. 18.
9. Dritsou, V., et al., *A draft genome sequence of an invasive mosquito: an Italian Aedes albopictus.* Pathog Glob Health, 2015. **109**(5): p. 207-20.
10. Chen, X.G., et al., *Genome sequence of the Asian Tiger mosquito, Aedes albopictus, reveals insights into its biology, genetics, and evolution.* Proc Natl Acad Sci U S A, 2015. **112**(44): p. E5907-15.
11. Giraldo-Calderon, G.I., et al., *VectorBase: an updated bioinformatics resource for invertebrate vectors and other organisms related with human diseases.* Nucleic Acids Res, 2015. **43**(Database issue): p. D707-13.
12. Goubert, C., et al., *De novo assembly and annotation of the Asian tiger mosquito (Aedes albopictus) repeatome with dnaPipeTE from raw genomic reads and comparative analysis with the yellow fever mosquito (Aedes aegypti).* Genome Biol Evol, 2015. **7**(4): p. 1192-205.
13. Nene, V., et al., *Genome sequence of Aedes aegypti, a major arbovirus vector.* Science, 2007. **316**(5832): p. 1718-23.
14. Dudchenko, O., et al., *De novo assembly of the Aedes aegypti genome using Hi-C yields chromosome-length scaffolds.* Science, 2017.
15. Matthews, T.C. and G.B. Craig, *Genetic heterozygosity in natural populations of the tree-hole mosquito Aedes triseriatus.* Annals of the Entomological Society of America, 1980. **73**(6): p. 739-743.
16. Alexander, D. *Quiver FAQ*. 2016 [cited 2017 June 27]; Available from: https://github.com/PacificBiosciences/GenomicConsensus/blob/master/doc/FAQ.rst.
17. Chin, C.S., et al., *Phased diploid genome assembly with single-molecule real-time sequencing.* Nat Methods, 2016. **13**(12): p. 1050-1054.
18. Coordinators, N.R., *Database Resources of the National Center for Biotechnology Information.* Nucleic Acids Res, 2017. **45**(D1): p. D12-D17.
19. Koren, S., et al., *Canu: scalable and accurate long-read assembly via adaptive k-mer weighting and repeat separation.* Genome Res, 2017. **27**(5): p. 722-736.
20. Maeder, H., et al. *PacBio® variant and consensus caller*. 2017 [cited 2017 June 27]; Available from: https://github.com/PacificBiosciences/GenomicConsensus.
21. Ferrari, J.A. and K.S. Rai, *Phenotypic correlates of genome size variation in Aedes albopictus.* Evolution, 1989. **43**(4): p. 895-899.
22. Sork, V.L., et al., *First Draft Assembly and Annotation of the Genome of a California Endemic Oak Quercus lobata Nee (Fagaceae).* G3 (Bethesda), 2016.
23. Simao, F.A., et al., *BUSCO: assessing genome assembly and annotation completeness with single-copy orthologs.* Bioinformatics, 2015. **31**(19): p. 3210-2.
24. NCBI. *NCBI Aedes albopictus Annotation Release 101*. 2017 [cited 2017 June 27]; Available from: https://www.ncbi.nlm.nih.gov/genome/annotation_euk/Aedes_albopictus/101/.
25. Kent, W.J. *Minimal Steps For LiftOver*. 2014 [cited 2017 June 27]; Available from: http://genomewiki.ucsc.edu/index.php/Minimal_Steps_For_LiftOver.
26. Hall, A.B., et al., *SEX DETERMINATION. A male-determining factor in the mosquito Aedes aegypti.* Science, 2015. **348**(6240): p. 1268-70.
27. Hall, A.B., et al., *Radical remodeling of the Y chromosome in a recent radiation of malaria mosquitoes.* Proc Natl Acad Sci U S A, 2016. **113**(15): p. E2114-23.
28. Hall, A.B., et al., *Six novel Y chromosome genes in Anopheles mosquitoes discovered by independently sequencing males and females.* BMC Genomics, 2013. **14**: p. 273.

29. Hall, A.B., et al., *Insights into the preservation of the homomorphic sex-determining chromosome of Aedes aegypti from the discovery of a male-biased gene tightly linked to the M-locus.* Genome Biol Evol, 2014. **6**(1): p. 179-91.
30. Avila-Bonilla, R.G., et al., *Analysis of the miRNA profile in C6/36 cells persistently infected with dengue virus type 2.* Virus Res, 2017. **232**: p. 139-151.
31. Paterson, A., et al., *Mosquito densonucleosis viruses cause dramatically different infection phenotypes in the C6/36 Aedes albopictus cell line.* Virology, 2005. **337**(2): p. 253-61.
32. Brackney, D.E., et al., *C6/36 Aedes albopictus cells have a dysfunctional antiviral RNA interference response.* PLoS Negl Trop Dis, 2010. **4**(10): p. e856.
33. Morazzani, E.M., et al., *Production of virus-derived ping-pong-dependent piRNA-like small RNAs in the mosquito soma.* PLoS Pathog, 2012. **8**(1): p. e1002470.
34. Campbell, C.L., et al., *Aedes aegypti uses RNA interference in defense against Sindbis virus infection.* BMC Microbiol, 2008. **8**: p. 47.
35. Crochu, S., et al., *Sequences of flavivirus-related RNA viruses persist in DNA form integrated in the genome of Aedes spp. mosquitoes.* J Gen Virol, 2004. **85**(Pt 7): p. 1971-80.
36. Chen, S., et al., *Genetic, biochemical, and structural characterization of a new densovirus isolated from a chronically infected Aedes albopictus C6/36 cell line.* Virology, 2004. **318**(1): p. 123-33.
37. Huang da, W., B.T. Sherman, and R.A. Lempicki, *Systematic and integrative analysis of large gene lists using DAVID bioinformatics resources.* Nat Protoc, 2009. **4**(1): p. 44-57.
38. Huang da, W., B.T. Sherman, and R.A. Lempicki, *Bioinformatics enrichment tools: paths toward the comprehensive functional analysis of large gene lists.* Nucleic Acids Res, 2009. **37**(1): p. 1-13.
39. Esquivel, C.J., B.J. Cassone, and P.M. Piermarini, *A de novo transcriptome of the Malpighian tubules in non-blood-fed and blood-fed Asian tiger mosquitoes Aedes albopictus: insights into diuresis, detoxification, and blood meal processing.* PeerJ, 2016. **4**: p. e1784.
40. Yang, Z., et al., *Dynamic expression of genes encoding subunits of inward rectifier potassium (Kir) channels in the yellow fever mosquito Aedes aegypti.* Comp Biochem Physiol B Biochem Mol Biol, 2017. **204**: p. 35-44.
41. Yang, L., D.L. Denlinger, and P.M. Piermarini, *The diapause program impacts renal excretion and molecular expression of aquaporins in the northern house mosquito, Culex pipiens.* J Insect Physiol, 2017. **98**: p. 141-148.
42. Drake, L.L., et al., *The Aquaporin gene family of the yellow fever mosquito, Aedes aegypti.* PLoS One, 2010. **5**(12): p. e15578.
43. Drake, L.L., S.D. Rodriguez, and I.A. Hansen, *Functional characterization of aquaporins and aquaglyceroporins of the yellow fever mosquito, Aedes aegypti.* Sci Rep, 2015. **5**: p. 7795.
44. Liu, K., et al., *Aquaglyceroporin function in the malaria mosquito Anopheles gambiae.* Biol Cell, 2016. **108**(10): p. 294-305.
45. Liu, K., et al., *Aquaporin water channel AgAQP1 in the malaria vector mosquito Anopheles gambiae during blood feeding and humidity adaptation.* Proc Natl Acad Sci U S A, 2011. **108**(15): p. 6062-6.
46. Raphemot, R., et al., *Discovery and characterization of a potent and selective inhibitor of Aedes aegypti inward rectifier potassium channels.* PLoS One, 2014. **9**(11): p. e110772.
47. Raphemot, R., et al., *Eliciting renal failure in mosquitoes with a small-molecule inhibitor of inward-rectifying potassium channels.* PLoS One, 2013. **8**(5): p. e64905.

48. Swale, D.R., et al., *An insecticide resistance-breaking mosquitocide targeting inward rectifier potassium channels in vectors of Zika virus and malaria.* Sci Rep, 2016. **6**: p. 36954.
49. Piermarini, P.M., et al., *Localization and role of inward rectifier K(+) channels in Malpighian tubules of the yellow fever mosquito Aedes aegypti.* Insect Biochem Mol Biol, 2015. **67**: p. 59-73.
50. Raphemot, R., et al., *Molecular and functional characterization of Anopheles gambiae inward rectifier potassium (Kir1) channels: a novel role in egg production.* Insect Biochem Mol Biol, 2014. **51**: p. 10-9.
51. Rouhier, M.F., et al., *Pharmacological validation of an inward-rectifier potassium (Kir) channel as an insecticide target in the yellow fever mosquito Aedes aegypti.* PLoS One, 2014. **9**(6): p. e100700.
52. Daly, G.M., et al., *Host Subtraction, Filtering and Assembly Validations for Novel Viral Discovery Using Next Generation Sequencing Data.* PLoS One, 2015. **10**(6): p. e0129059.
53. Moser, L.A., et al., *A Universal Next-Generation Sequencing Protocol To Generate Noninfectious Barcoded cDNA Libraries from High-Containment RNA Viruses.* mSystems, 2016. **1**(3).
54. Jiao, W.B. and K. Schneeberger, *The impact of third generation genomic technologies on plant genome assembly.* Curr Opin Plant Biol, 2017. **36**: p. 64-70.
55. Adey, A., et al., *The haplotype-resolved genome and epigenome of the aneuploid HeLa cancer cell line.* Nature, 2013. **500**(7461): p. 207-11.
56. Weisenfeld, N.I., et al., *Direct determination of diploid genome sequences.* Genome Res, 2017. **27**(5): p. 757-767.
57. Seo, J.S., et al., *De novo assembly and phasing of a Korean human genome.* Nature, 2016. **538**(7624): p. 243-247.
58. Eleftherianos, I., et al., *ATP-sensitive potassium channel (K(ATP))-dependent regulation of cardiotropic viral infections.* Proc Natl Acad Sci U S A, 2011. **108**(29): p. 12024-9.
59. Rouhier, M.F. and P.M. Piermarini, *Identification of life-stage and tissue-specific splice variants of an inward rectifying potassium (Kir) channel in the yellow fever mosquito Aedes aegypti.* Insect Biochem Mol Biol, 2014. **48**: p. 91-9.
60. Piermarini, P.M., et al., *Cloning and functional characterization of inward-rectifying potassium (Kir) channels from Malpighian tubules of the mosquito Aedes aegypti.* Insect Biochem Mol Biol, 2013. **43**(1): p. 75-90.
61. Overend, G., et al., *A comprehensive transcriptomic view of renal function in the malaria vector, Anopheles gambiae.* Insect Biochem Mol Biol, 2015. **67**: p. 47-58.
62. Tsujimoto, H., et al., *Organ-specific splice variants of aquaporin water channel AgAQP1 in the malaria vector Anopheles gambiae.* PLoS One, 2013. **8**(9): p. e75888.
63. Pendleton, M., et al., *Assembly and diploid architecture of an individual human genome via single-molecule technologies.* Nat Methods, 2015. **12**(8): p. 780-6.
64. Meyers, E. *Damasker: The Dazzler Repeat Masking Suite*. 2016 [cited 2016 hash ebc0de1a]; Available from: https://github.com/thegenemyers/DAMASKER.
65. Walenz, B. *Canu Version 1.3*+ 2016 [cited 2016 hash d3dce1f449f403a250393d02868a1c6c14af62e6]; Available from: https://github.com/marbl/canu/releases.
66. Koren, S. *ArrowGrid*. 2017 [cited 2017 June 27]; Available from: https://github.com/skoren/ArrowGrid.
67. Smit, A.F.A. and R. Hubley. *RepeatModeler Open-1.0.* 2015 [cited 2016 Aug 29]; Available from: http://www.repeatmasker.org/.

68. Price, A.L., N.C. Jones, and P.A. Pevzner, *De novo identification of repeat families in large genomes.* Bioinformatics, 2005. **21**(suppl 1): p. i351-i358.
69. Bao, Z. and S.R. Eddy, *Automated de novo identification of repeat sequence families in sequenced genomes.* Genome research, 2002. **12**(8): p. 1269-1276.
70. Benson, G., *Tandem repeats finder: a program to analyze DNA sequences.* Nucleic acids research, 1999. **27**(2): p. 573-580.
71. Wootton, J.C. and S. Federhen, *Statistics of local complexity in amino acid sequences and sequence databases.* Computers & Chemistry, 1993. **17**(2): p. 149-163.
72. Vurture, G.W., et al., *GenomeScope: Fast reference-free genome profiling from short reads.* Bioinformatics, 2017.
73. Langmead, B. and S.L. Salzberg, *Fast gapped-read alignment with Bowtie 2.* Nat Methods, 2012. **9**(4): p. 357-9.
74. Li, H., et al., *The Sequence Alignment/Map format and SAMtools.* Bioinformatics, 2009. **25**(16): p. 2078-9.
75. Quinlan, A.R. and I.M. Hall, *BEDTools: a flexible suite of utilities for comparing genomic features.* Bioinformatics, 2010. **26**(6): p. 841-2.
76. Kurtz, S., et al., *Versatile and open software for comparing large genomes.* Genome Biol, 2004. **5**(2): p. R12.
77. Istrail, S., et al., *Whole-genome shotgun assembly and comparison of human genome assemblies.* Proc Natl Acad Sci U S A, 2004. **101**(7): p. 1916-21.
78. Altschul, S.F., et al., *Basic local alignment search tool.* J Mol Biol, 1990. **215**(3): p. 403-10.
79. NCBI. *The NCBI Eukaryotic Genome Annotation Pipeline*. 2016 [cited 2017 June 27]; Available from: https://www.ncbi.nlm.nih.gov/genome/annotation_euk/process/.
80. Kent, W.J., *BLAT—the BLAST-like alignment tool.* Genome research, 2002. **12**(4): p. 656-664.
81. Smit, A.F.A., R. Hubley, and P. Green. *RepeatMasker Open-4.0*. 2015 [cited 2016 Aug 29]; Available from: http://www.repeatmasker.org/.
82. Langmead, B., et al., *Ultrafast and memory-efficient alignment of short DNA sequences to the human genome.* Genome Biol, 2009. **10**(3): p. R25.
83. Miller, JR et al. (2017) Supporting data from "Analysis of the *Aedes albopictus* C6/36 genome provides insight into cell line utility for viral propagation". GigaScience Database. http://dx.doi.org/10.5524/100401

84. Hu, X., et al., The genomic sequence of the Chinese hamster ovary (CHO)-K1 cell line. Nature Biotech, 2011. **29**: p. 735-741.
85. Lewis, N., et al. Genomic landscapes of Chinese hamster ovary cell lines as revealed by the Cricetulus griseus draft genome. Nature Biotech, 2013. **31**: p. 759-765.
86. Hammon, S., et al. Genomic sequencing and analysis of a Chinese hamster ovary cell line using Illumina sequencing technology. BMC Genomics, 2011. **12**:67.

Figure 1

Click here to download Figure C636.Figure1.docx

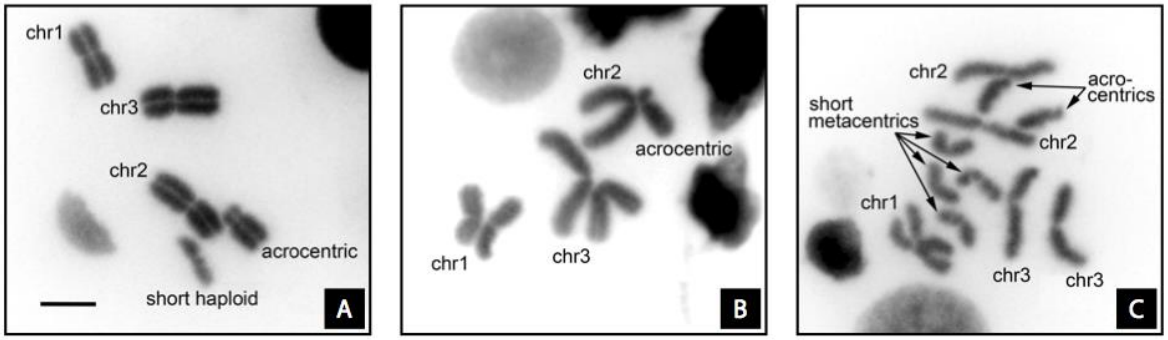

Figure 2 Click here to download Figure C636.Figure2.docx

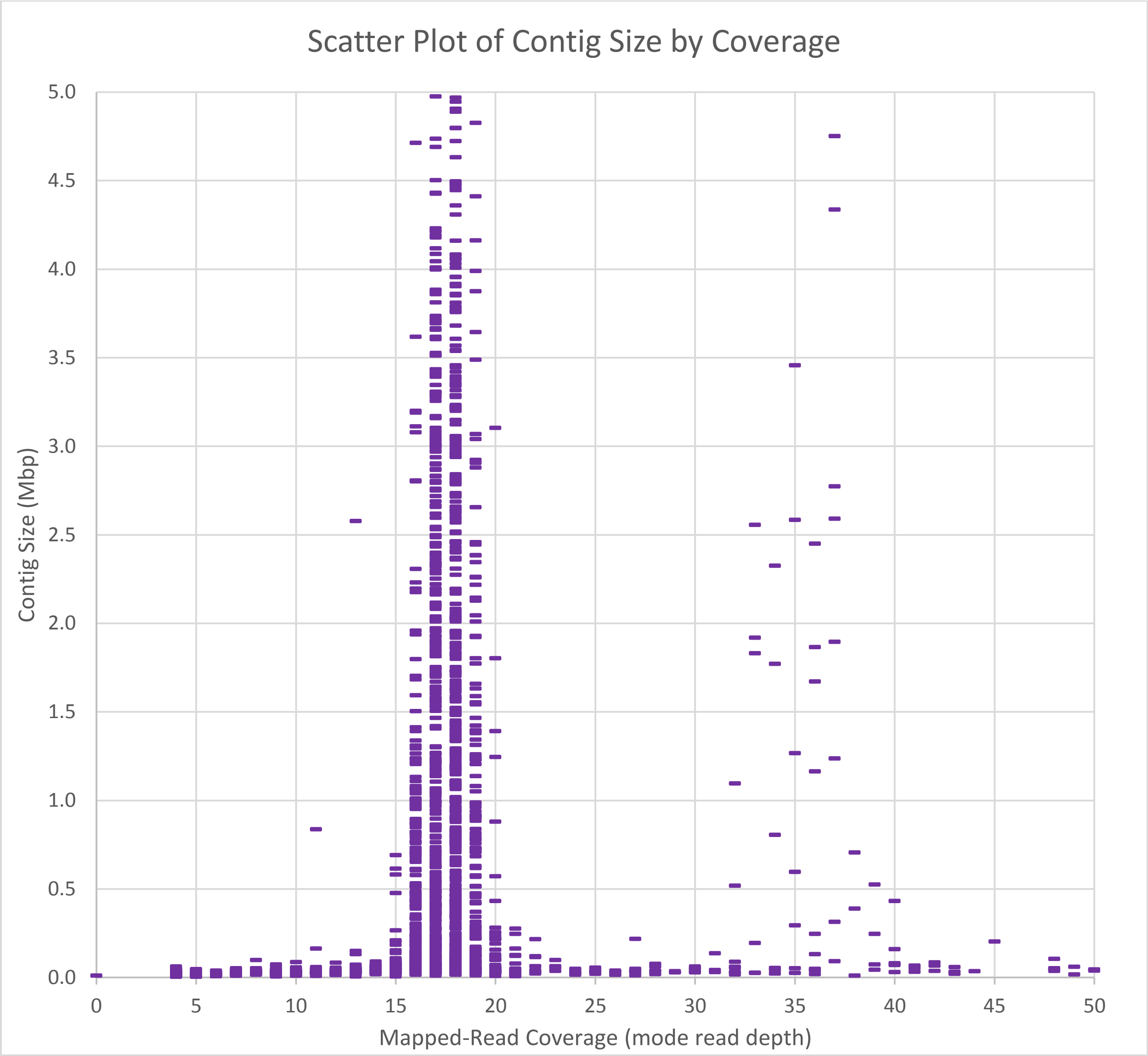

Click here to download Figure C636.Figure3.docx

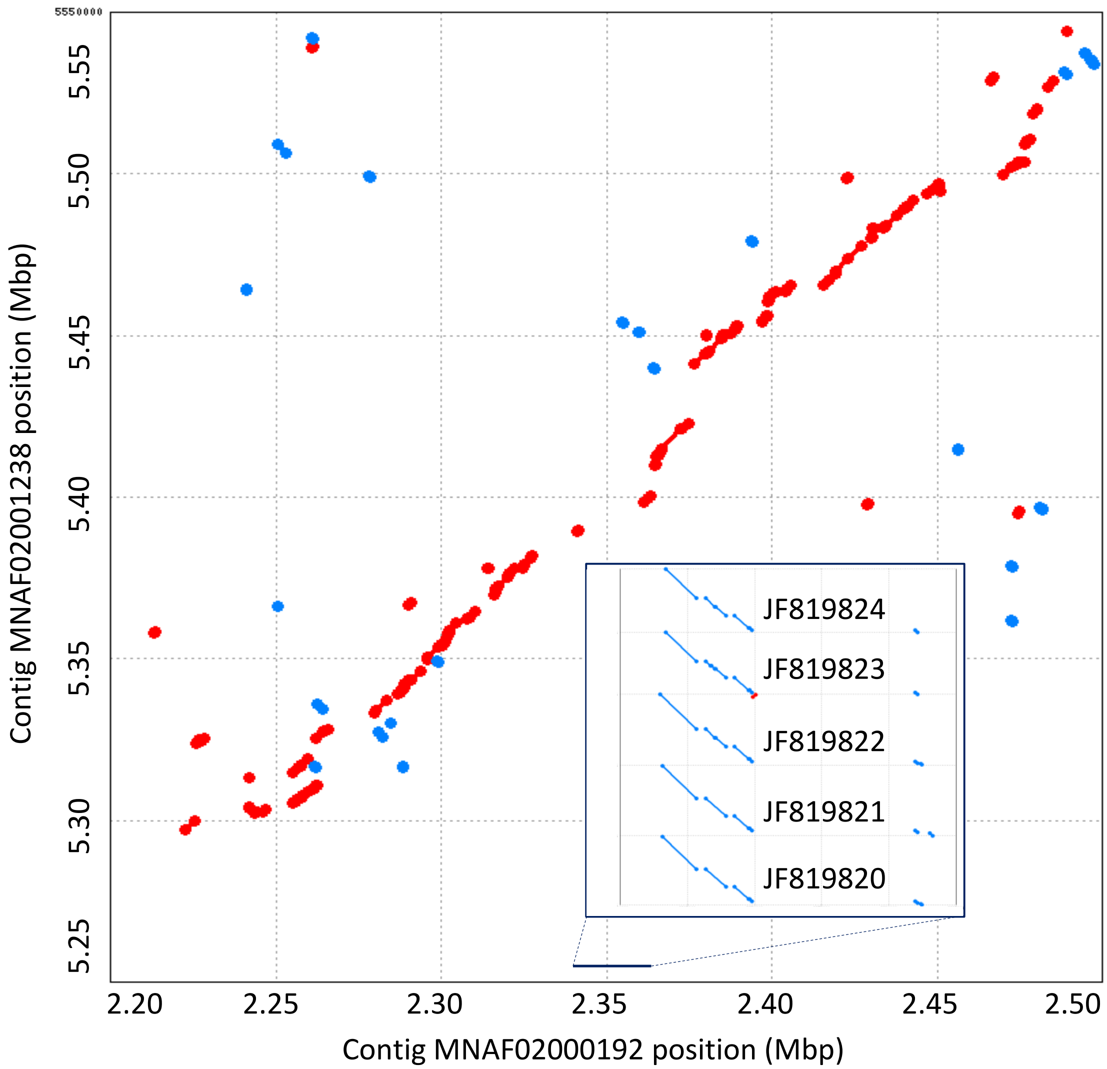

Subtable Detox_OBP

Click here to access/download

Supplementary Material

C636.SubTable.Detox_OBP.xlsx

Subtable AQP_Kir

Click here to access/download
Supplementary Material
C636.SubTable.AQP_Kir.xlsx

Subtable DAVID

Click here to access/download
Supplementary Material
C636.SubTable.DAVID.xlsx

Supplemental Figures

Click here to access/download
Supplementary Material
C636.SupplementalFigures.docx

Supplemental Tables

Click here to access/download
Supplementary Material
C636.SupplementalTables.xlsx

Authors' response to the Reviewers: We are grateful to the reviewers for their thoughtful feedback. We have implemented all of the Reviewer's suggestions. The revised manuscript is improved in very important ways. Our guide to changes is included here. Thank you.

Reviewer 1, Summary. In the manuscript, Miller and colleagues report their results on the genome assembly of the Aedes albopictus C6/36 cell line, an important laboratory cell line resource for studying mosquito-borne viruses. The author applied long-read PacBio DNA sequencing and Illumina NextSeq RNA sequencing to assemble and annotate the genome and transcriptome of the cell line. The analysis is appropriate and uses a range of computational programs for de novo assembly, repeat detection, genome annotation, RNA mapping, and cross-comparisons to a previous A. albopictus Foshan assembly. The results are valuable resources for future molecular studies of virus propagation and virus-mosquito vector interactions of several significant human-disease causing viruses, including Zika, Dengue, and Chikungunya viruses. I have several comments and suggestions below:

Reviewer 1, Point 1. The title "...insight into cell line adaptation..." needs to be revised, as the results presented are not sufficient to prove the adaptation of the current cell line compared to the original parental cell line for virus propagation. Direct comparisons to the parental cell line and better links to virus propagation are needed to prove the adaptation process.

Response:

This is a valid point and we agree. We have changed the title to remove the reference to adaptation: Analysis of the *Aedes albopictus* C6/36 genome provides insight into cell line utility for viral propagation.

Reviewer 1, Point 2. A limitation of the PacBio long read sequencing is the high sequencing error rate, and potential readers would be interested in the accuracy of the assembly. The current manuscript presents many results on the contig length and the coverage, but there is little discussion on the accuracy. The authors should discuss this aspect. The authors may consider moving one or more of the 17 supplementary tables into the main text to highlight their key findings. Likewise, Figure S3 may be considered moving into the main text.

Response:

It was an oversight to omit consensus accuracy analysis. In our initial submission, the lone indicator of consensus quality was present in Supplemental Table S4 which showed mismatch rate = 0.70 in mapped Illumina reads. We have now included new analysis in the main text by adding a paragraph to the end the Sequencing and Assembly section of Results. Unfortunately, we lack references with which to derive the average QV per assembled base. Instead, we present statistics showing a high level of agreement with the short read sequencing which was not used during assembly: The mapped reads covered 98.98% of assembled bases and confirmed 99.30% of aligned bases; 74.66% of mapped reads aligned end-to-end with zero mismatches and indels.

We agree that some readers may be surprised to see high consensus accuracy in an assembly of high-error reads. To address this, we added a reference to our prior study of consensus quality in Canu assemblies in which consensus quality surpassed QV=30 (99.9% accuracy) on several data sets: These results are consistent with prior analyses, *e.g.* 99.98% identity to the *Drosophila melanogaster* reference achieved by a Canu+Quiver assembly of 90X P5C3 PacBio [19].

The Reviewer suggested including some of our supplemental tables or figures in the main text. We have acted on the Reviewer's specific suggestion to move Supplemental Figure S3, to the main text. The text now includes a new Figure 3 formed from a montage of two figures from Figure S3, which remains in the supplement unchanged. Figure 3, which is about the Dicer locus, covers one of our important gene findings and simultaneously illustrates the haplotype separated nature of our assembly.

Reviewer 1, Point 3. Table 2 shows weak results on the enrichment of the two broad categories: cytoskeletal functions and cell signaling. The suggestion that these two categories can be cell line specialisations for growth in the laboratory culture should be raised with caution. DAVID analysis based on 1,310 highly expressed transcripts (with selected thresholds >=2x the RPKM means and enrichment score >1.3) and manual categorisation of 22 functional clusters are not rigorous statistical analysis. Selection of highly expressed genes may lead to a biased analysis of abundant structural genes (cytoskeleton genes), metabolic genes, and house-keeping genes such as those for ribosomal genes (the top most enriched). More stringent gene-ontology enrichment or network analysis (with appropriate background genes, more specific sub/child ontologies,

and possible comparisons with other cell lines as base lines) are needed to support the conclusion on the enrichment of the two broad categories
(cytoskeletal function and cell signaling).

Response:

We thank the reviewer for this comment. We agree that a more comprehensive analysis is desirable. We plan to pursue future studies focused on the transcriptomics of this cell line. Given the limited nature of our transcriptomics data, our preference is to maintain our current analysis, which allows to compare and contrast with a previously published study by our group on *Ae. albopictus* Malpighian tubules. Nevertheless, we have softened our language when describing and interpreting the DAVID analysis to emphasize the preliminary nature of these results and need to confirm in future studies, and we added the conclusion: Further studies are required to test this hypothesis.

Reviewer 1, Point 4. The analysis and evidence to support the conclusion that the cell line is deficient in aquaporins and inward rectifier K+ channel need to be described with more details. Was the analysis based on the expression values (RPKM) measured from a single RNA sequencing sample? Were multiple mapped reads to AQP and Kir genes removed by the mapping and transcript counting pipelines (This may lead to low RPKM for these genes)? More biological/technical replicates may confirm whether the genes were not expressed or lowly expressed. Comparing with data from other studies on the expression of AQP and Kir genes relative to other more abundant genes may help to support the conclusion.

Response:

The reviewer requests additional details on the experiment that was done and suggests additional comparisons that should be done.

We agree that additional data would be helpful but we regret that we are not able to provide it due to funding constraints. Instead, we have softened the statement on the deficiency of AQPs and Kir channels, which we agree had too strong of a conclusion. The transcriptomics data certainly suggest weak expression of these mRNAs, but we now emphasize that additional functional studies are required to confirm weak physiological activity of these channels.

The requested details were possibly already present in the manuscript. The text at the start of the transcriptomics results section says transcripts were tested for presence or absence based on a single run of RNAseq and that RPKM was computed per transcript without consolidation per gene. We have revised our manuscript so that readers will be referred to these methods descriptions. Specifically, we added this sentence at the end of the transcriptomics results section: Our findings, derived from analysis of a single RNAseq run as described above, warrant further study. Also, we added the detail that the mapping retained at most one mapping per read, which was previously mentioned only in the methods section.

Reviewer 1, Point 5. The implications of a subtraction database for viral detection is interesting. The authors may add discussion on how the multiplex sequencing method with the use of the subtraction database outperforms the quantitative PCR method in cost, sensitivity, and time.

Response:

If the question is whether a specific virus is present, the qPCR method would be preferable by cost, sensitivity, and time. The subtraction method is preferable if the question is what viruses, if any, are present. The subtraction method could be considered a filter for a metagenomics analysis. To clarify this point for readers, we have added text to the section on subtraction results. The new text emphasizes that qPCR was included as a control here but it would not be part of an actual subtraction experiment: After this control measure, the remainder of the experiment emulated a search for any virus in cells exposed to an uncharacterized sample.

Reviewer 1, Point 6. Figure 2 axis labels should be included in the figure, not in the legend.

Response:

Figure 2 has been revised. Thank you for this suggestion, which we also applied to the new Figure 3.

Reviewer 2, Summary. Cell lines are critical platforms for understanding biology of an organism; however, over the time the cell lines drift from the genome. In order to achieve

meaningful results, it is important to know the cell and tissue types the cell lines were originally derived from. The manuscript sheds light on two important considerations in the use of a cell line for understanding mosquito-virus interaction:

1. It has drifted from the Ae. albopictus genome and shows lack of aquaporin transcripts which is important to know if a researcher is interested in these genes.
2. The cell line is potentially derived from male larvae and therefore might not be suitable for testing female specific gene pathways. As the authors' suggested this finding can potentially be used for testing sex-specific agents developed for mosquito sterility.

Reviewer 2, Point 1. This is important work and worthy of publication. However, I am not fully convinced that the library prep and sequencing at two different institutions (DNA from lot # 59479117 was sequenced at NBACC (Fort Detrick, MD) using 128 SMRT cells and multiple libraries. DNA from lot #62871143 was sequenced at Icahn School of Medicine at Mt. Sinai (NY) using 80 SMRT cells) will provide data for direct comparison. The differences in sample handling and library prep will result in variations; however, I am of the opinion that it will not change the authors' conclusion. The authors still need to explain why the sequencing was carried out at two different sites.

Response:

The C6/36 assembly is derived from two lots of cells, both labeled C6/36 and provided by ATCC. Each was sequenced at a different lab but both used the PacBio RS II instrument with P6C4 chemistry and the manufacturer's recommended prep. The reason for the separate origins of cells is that our experiment represents a merger of two formerly independent projects. Both projects had similar designs and both were underway when the project leaders discovered the redundancy during discussions at a conference. Our groups decided to pool resources in order to generate a high-quality assembly that would exploit roughly twice the sequencing depth as either project could provide separately. We hope the community benefits from our collaboration.

We added the following historical and parenthetical remark to the description of sequencing in the Methods section: (Two formerly independent sequencing projects combined resources to generate one high-coverage assembly.)

We also added a sentence to assuage concerns that the two lots may have assembled separately. We added this sentence to the Methods section near the text that

contained the description of the separate lots: With the exception of 30 contigs representing 1% of assembled bases, all contigs contained reads from both samples.

Other changes:

The original draft included the claim that C6/36 was the second cell line to have its genome *de novo* assembled. One of us since found three publications on the CHO cell line. We revised the Discussion section to cite the publications of CHO and HeLa genomes. The revised text makes no claim to rank order: C6/36 joins CHO [83,84,85] and HeLa [55] as another cell line to have its genome *de novo* assembled. The new references were added to the end of the current references to make clear which references are new. We will renumber all references prior to publication.

We revised the supplemental material to reflect an update to RefSeq that occurred after our prior manuscript submission. The RefSeq annotation of the C6/36 Dicer locus formerly described a putatively transcribed gene with the note that the contig consensus sequence was missing one base. The revised RefSeq annotation accepts the consensus and describes a pseudogene. Therefore, we revised the caption to Supplemental Figure S3c-d to include the sentence: We have updated the RefSeq annotation to note a "polymorphic pseudogene" at LOC109403945.

We inserted the word "RNAseq" where it could add clarity to descriptions of our mapped RNA sequencing data.

We added accessions where it could help users find the data at NCBI. We inserted RefSeq accessions with prefixes "LOC", "XP", and "NW" at various places in the main text. We added Supplemental Table S18 that lists the GenBank/MNAF and RefSeq/NW accessions for each contig.

We revised the caption to Figure 1, which is about karyotypes, to improve the clarity of the text.

We updated the affiliations for several authors.
