# Supplementary material for: Analysis of the Aedes albopictus C6/36 genome provides insight into cell line utility for viral propagation
Source: Gigascience. 2018 Jan 10;7(3):gix135. doi: 10.1093/gigascience/gix135 (PMC5869287; doi:10.1093/gigascience/gix135)
Supplement: Supplemental material [file gix135_supp.zip › C636.SupplementalFigures.docx]

*Aedes albopictus* C6/36 genome assembly. Supplemental Figures.


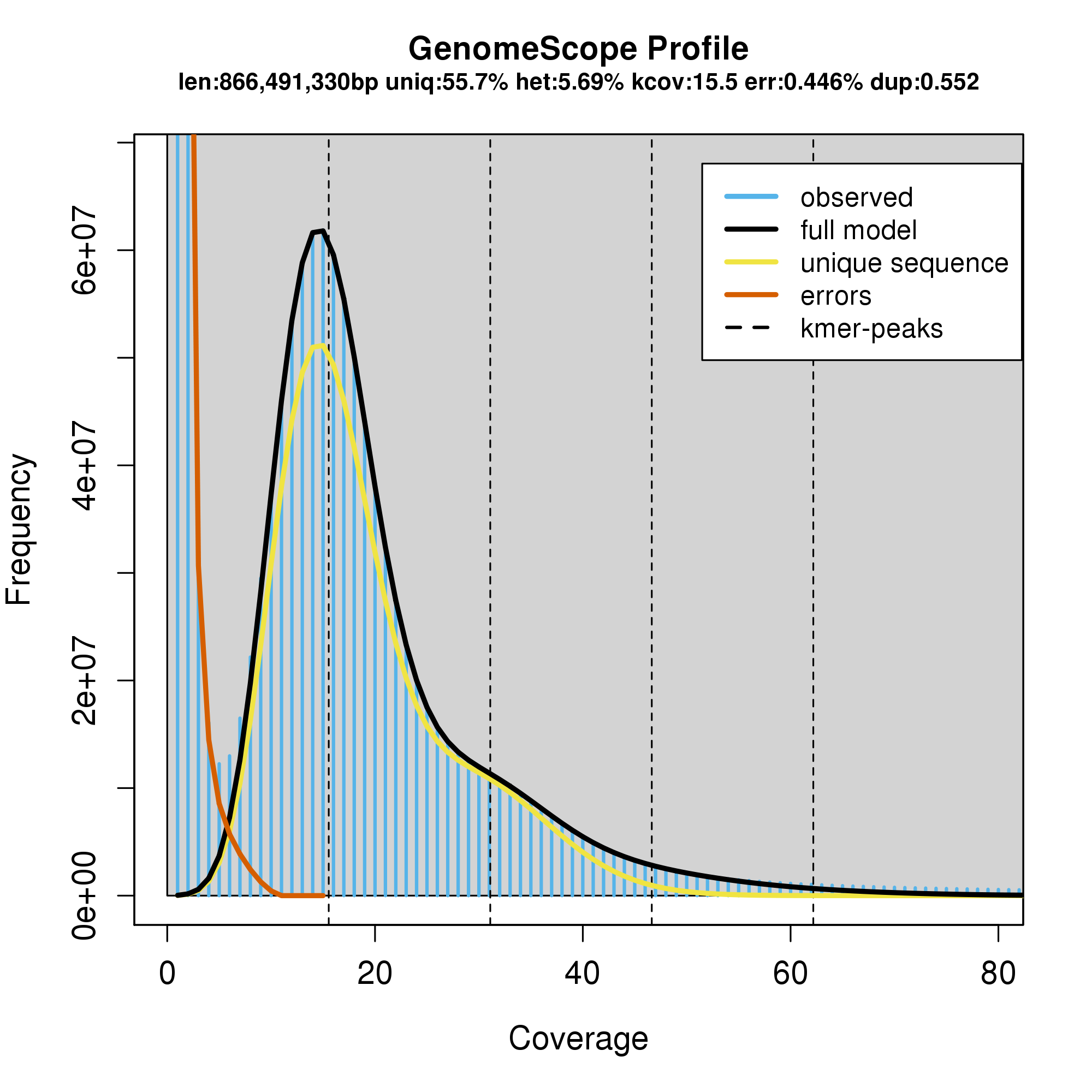


**Figure S1a**. K-mer frequency histogram analysis. The Illumina short reads were analyzed independent of the long-read assembly by counting subsequences in reads requiring exact match of 21 bp. The figure was generated by GenomeScope (1) version 1.0 with parameter k=21. The analysis detected a coverage peak at 15.5X and a slight shoulder at about 31X. It detected 0.45% error in the reads and predicted 5.7% heterozygosity in the genome. Its extrapolated genome size was 866,491,330 bases (haploid length). This predicts that an assembly that represents both haplotypes at every locus would span 1.7 Gbp. However, the C6/36 assembly has a 2.25 Gbp contig span.

**Figure S1b**. Overall coverage histogram. The Illumina genomic short-read sequence provides 20X overall coverage of contig span. Reads were mapped to contigs with bowtie2 (2). The depth of coverage in mapped reads was computed at every base using samtools (3) plus inference of zero-coverage bases. The distribution of number of bases (Y-axis) at each integer coverage bin (X-axis) has a mode at 18X.


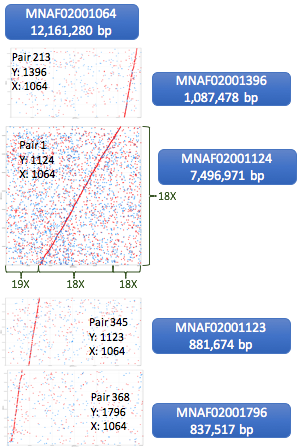


**Figure S2a**. A duplication captured in PC #1 (contig pairs ranked by number of mapped short-read pairs in common). The duplication extends to three other contig pairs: #213, #345, #368. Short read pairs were mapped to contigs allowing multiple placements. Using reads having exactly two maps, contigs sharing at least 10,000 maps were ranked by map count, aligned, and plotted. Coverage values (green) are shown for the duplicated and non-duplicated regions of both contigs in PC #1. The values are based on the single best alignment per read pair are shown for the aligned and unaligned spans of this pair. The coverage values are consistent with the assembly-wide coverage average, indicating this duplication is not an isolated assembly artifact. In this and subsequent figures, the X and Y axis are roughly scaled to the contig lengths. Individual contigs on the X and Y axis are identified by the last four digits of their public accession *e.g.* 1064 corresponds to MNAF02001064.1 in NCBI GenBank.


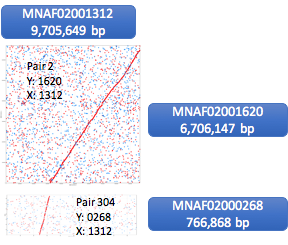


**Figure S2b**. A duplication captured in PC #2 extends to another contig pair, PC #304. The contig coverage is 17X-18X for these contigs.


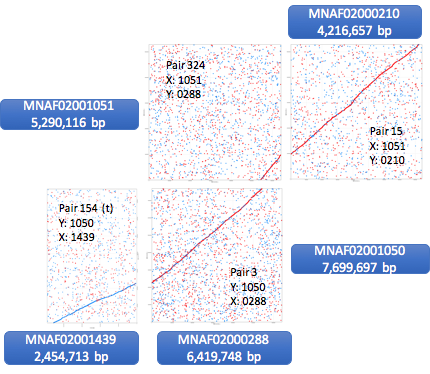


**Figure S2c**. A duplication captured in PC #3 extends to other pairs #15, #154, and #324. The visible diagonal, a conglomeration of line segments representing small alignments, is mostly red indicating the forward strand of both contigs was used in the alignment. The mostly blue diagonal for PC #154 merely indicates that the duplicated sequence was captured in reverse complement on contig 1439. Contig coverage is 17X in all contigs.


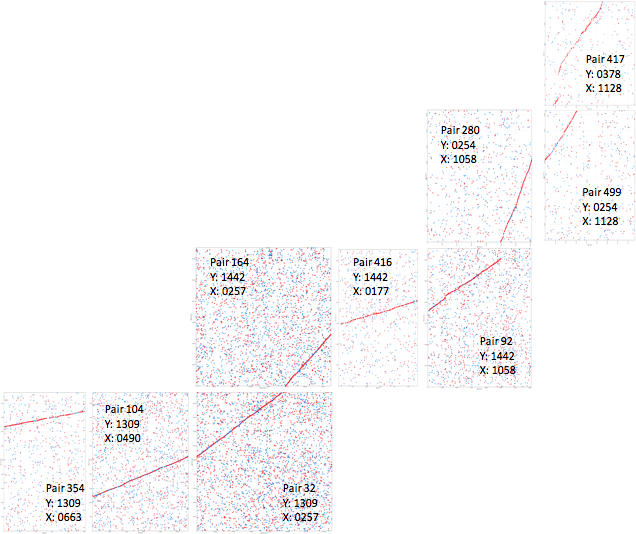


**Figure S2d**. An example of a duplication traversing many contigs. The putative duplication spans about 18 Mbp. Pair #354 (left) provides evidence of further genome duplication, as both contigs contain sequence similar to sequence in both contigs of PC #32. Pairs #499 and #417 (righ) suggest transposition events in the genome. Contig coverage range is 16X to 19X for all contigs shown. Each image shows entire contigs on the X and Y axiis. Each image is roughly scaled to the contig sizes but the variable slope of the diagonals is an artifact.


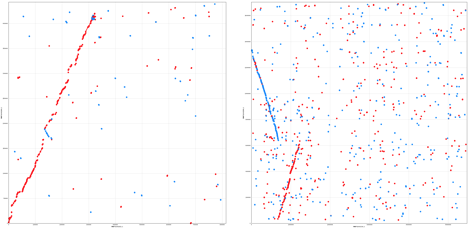


**Figure S2e.** Examples of rearrangements within duplicated sequence. Found by visual inspection, the inversion is highlighted by a red diagonal of dots indicating forward strand alignments near a blue diagonal indicating negative strand alignment. There is a ~25 Kbp inversion in pair 497 (left) and a ~600 Kbp inversion in pair 477 (right). Pair 497 aligns MNAF02002268 to MNAF02002023 on Y and X axis respectively. Pair 477 aligns MNAF02001588 to MNAF02001314.


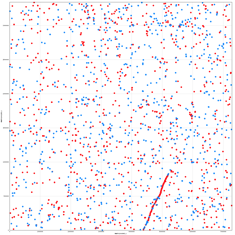


**Figure S2f**. An alignment that ends mid-contig. Found by visual inspection, pair 504 aligns and MNAF02001255 to MNAF02000945 on Y and X axis respectively. The diagonal spans about 1 Mbp.


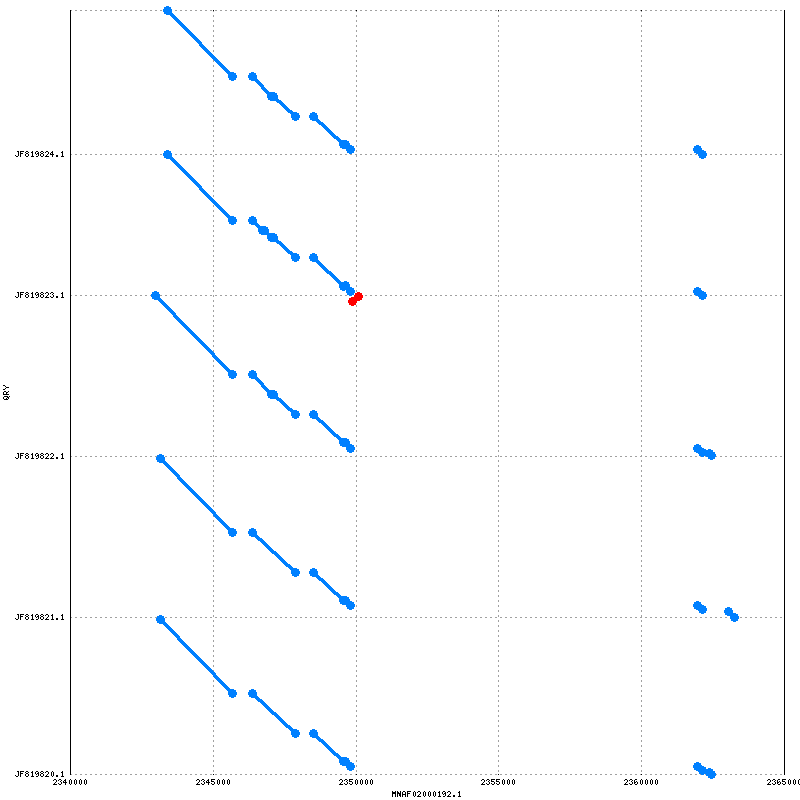

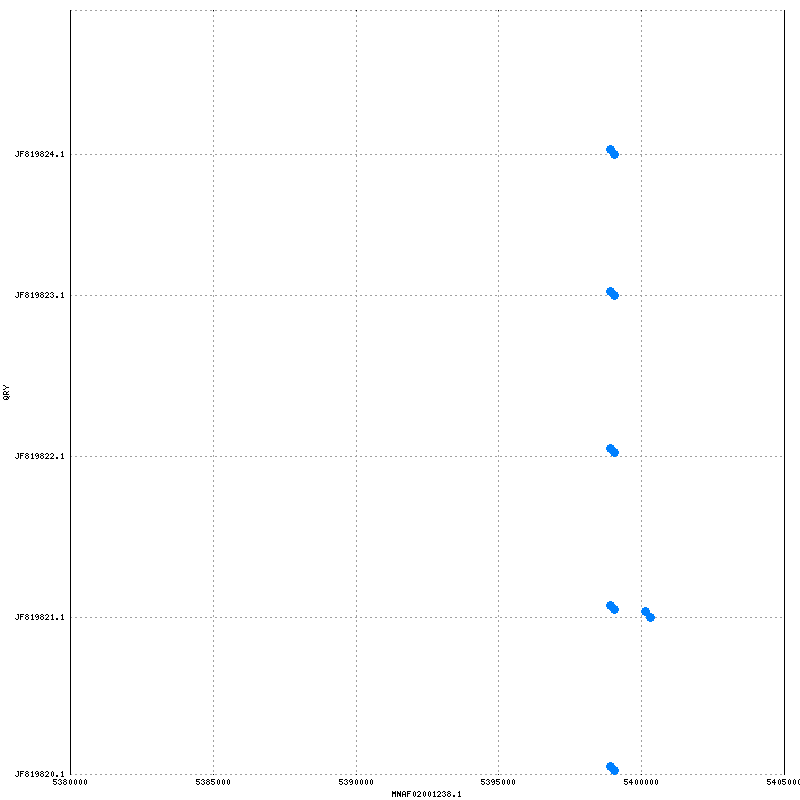


**Figure S3a-b**. Alignments of Dicer transcripts to C6/36 contigs. Five previously published *Aedes albopictus* transcripts have a nearly full-length alignment to contig MNAF02000192.1 *i.e.* scaffold NW_017856188.1 (**a**, left) and partial alignments to contig MNAF02001238.1 *i.e.* scaffold NW_017857234.1 (**b**, right) in the C6/36 assembly. In these plots, five transcripts are in 5 tiers (Y-axis) with blue diagonals representing alignments from transcript 3’ toward upper left to 5’ toward lower right. The transcript 5’ ends map to both contigs but these regions map 13 Kbp from the remaining transcript sequence when present. A portion of one transcript appears repetitive, having a redundant alignment to the reverse strand (red) and to other contigs (not shown). The alignments were computed with nucmer (--nosimplify) and plotted with mummerplot (default parameters) (4). The X-axis, zoomed to the 25 Kbp region of interest on both contigs. Short-read coverage is at haploid level across these regions; average coverage of the ten 5 Kbp regions shown is 18.7X ± 0.7. The Y-axis represents five full-length *A. albopictus* Dicer transcripts reported previously (5).

In the order displayed, the transcripts are:

- JF819824.1 Aedes albopictus cell-line U4.4 Dicer 2 (Dcr-2) mRNA, complete cds
- JF819823.1 Aedes albopictus cell-line C7-10 Dicer 2 (Dcr-2) mRNA, complete cds
- JF819822.1 Aedes albopictus cell-line C6/36 Dicer 2 (Dcr-2) mRNA, complete cds
- JF819821.1 Aedes albopictus Dicer 2 isoform B (Dcr-2) mRNA, complete cds
- JF819820.1 Aedes albopictus Dicer 2 isoform A (Dcr-2) mRNA, complete cds

**Figure S3c-d**. Annotation at the *dcr-2* frameshift mutation. According to a previous report, “Genotyping a single nucleotide deletion identified in the C6/36 *dcr-2* ORF revealed a homozygous frameshift mutation (FS 21) resulting in a premature termination codon” (5). The transcript maps to C6/36 contig MNAF02000192 (NW_017856188). The mapped location had been annotated as endonuclease Dicer, LOC109403945 by RefSeq. The original automatic annotation assigned the transcript XM_019676844 and protein XP_019532389 accessions, which have since been replaced. The automatic annotation noted a predicted transcript, supported by 99% coverage by *A. albopictus* RNAseq, whose “sequence of the model RefSeq protein was modified relative to its source genomic sequence to represent the inferred CDS: inserted 1 base in 1 codon”. The predicted transcript sequencing containing the ‘N’ has a long open reading frame (ORF 10 in **c**, top) that is truncated if the ‘N’ is removed (ORF 7 in **d**, bottom). We have updated the RefSeq annotation to note a “polymorphic pseudogene” at LOC109403945. Transcript XM_019676844 was replaced by NM_001352974 and protein XP_019532389 was replaced by NP_001339903. The images were generated with the NCBI Open Reading Frame Viewer (<https://www.ncbi.nlm.nih.gov/orffinder/)>.


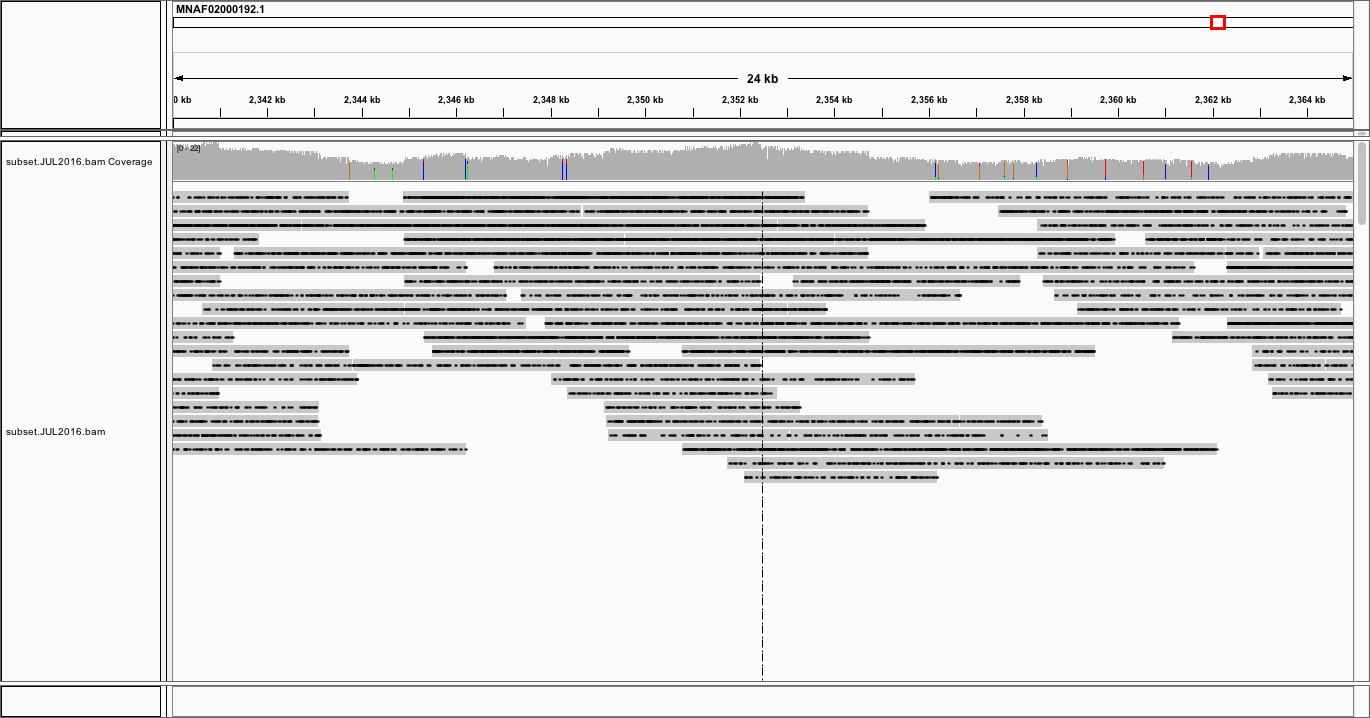


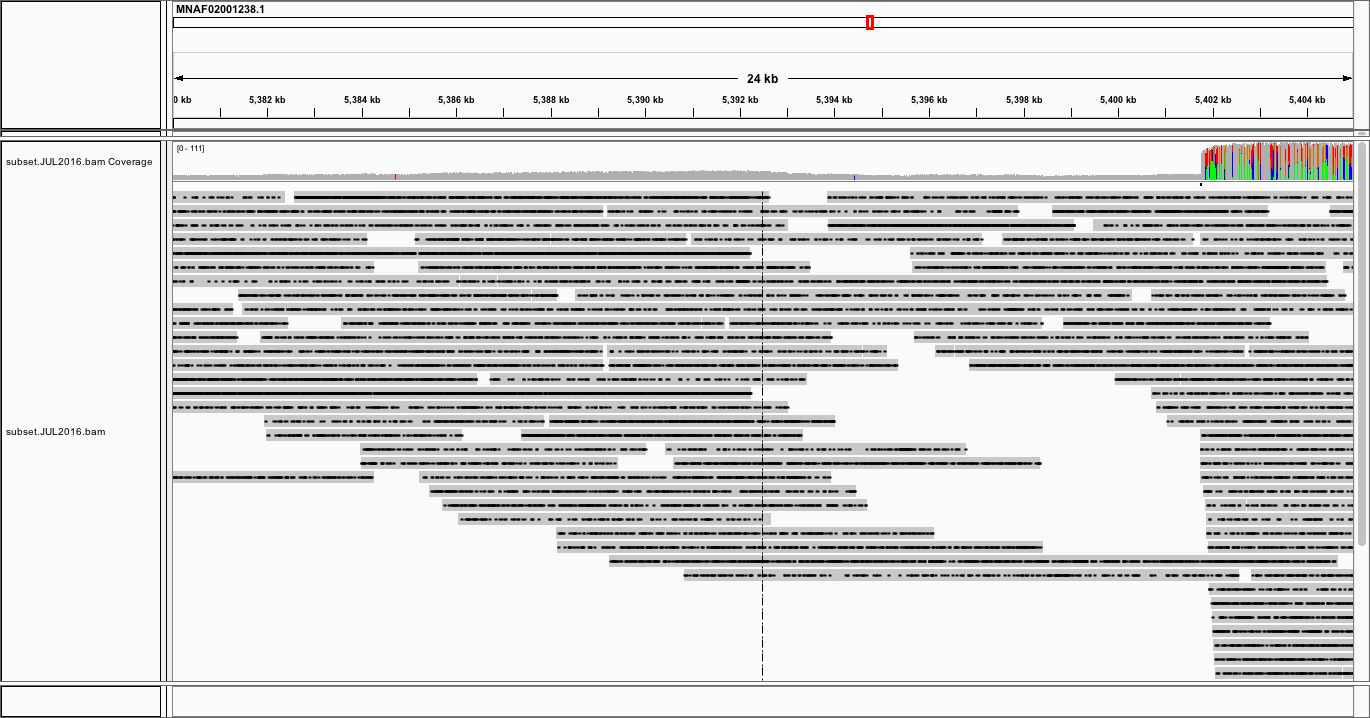


**Figure S3e-f**. Both Dicer regions have long-read support. Contig MNAF02000192 region 2340-2365 Kbp (**c**, top) corresponds to **Figure S3a**. Contig MNAF02001238 region 5380-5405 Kbp (**d**, bottom) corresponds to **Figure S3b**. Contigs are represented on the horizontal axis. Aligned reads are represented by grey rectangles at an arbitrary vertical position chosen for visual clarity. Dark regions within grey boxes indicate insertions (the predominant error type in uncorrected PacBio reads). A coverage histogram is depicted below each axis with inferred SNPs in color; the region of intense color appears to be a genomic repeat that has attracted reads derived from other repeat instances. To generate the images, a subset of the raw PacBio subreads were mapped to both contigs with blasr 1.3.1.140182 ‘-minPctIdentity 80 -minReadLength 1000 -minMatch 14 -bestn 1 -clipping soft –noSplitSubreads’ (6), filtered for alignments 4 Kbp or longer, and displayed with IGV 2.3.94 (7).


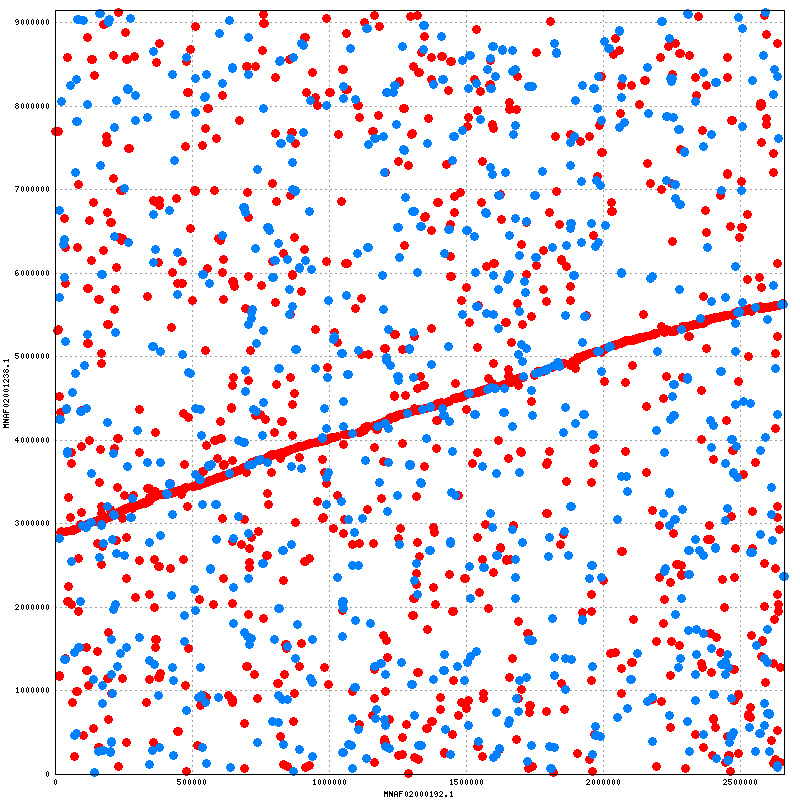

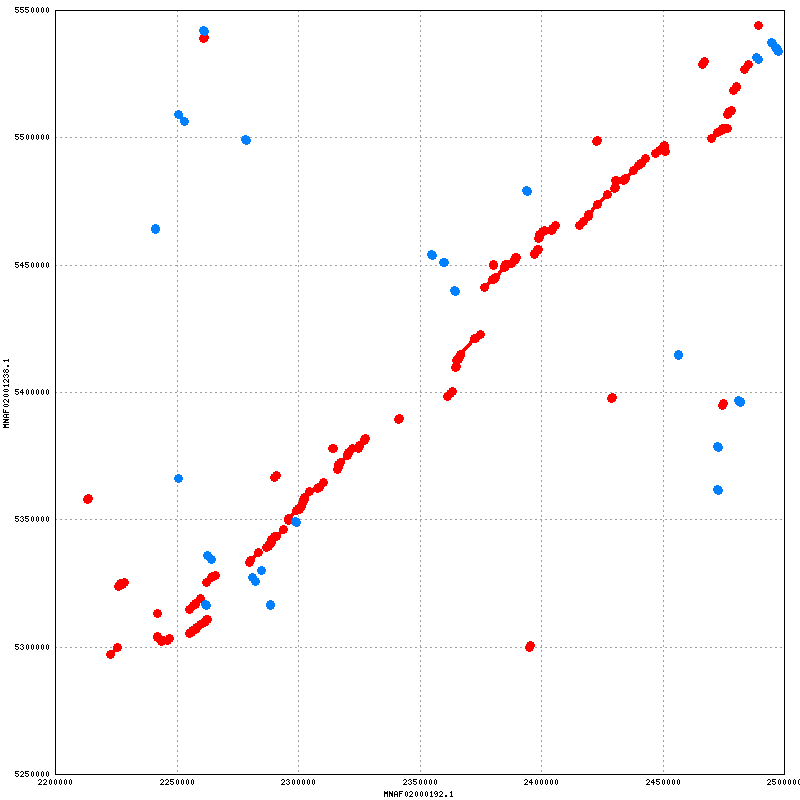


**Figure S3g-h**. Alignment of the Dicer contigs. The two contigs with Dicer sequence were aligned to each other. The alignment spans all of the shorter contig (**e**, left) but leaves a gap at the Dicer locus, which is at the center of the X and Y axis of the zoomed in view (**f**, right). Image on left includes full lengths of both contigs and unequal scaling takes the alignment off the diagonal; contig MNAF02000192 (X-axis) is 2.5 Mbp while contig MNAF02001238 (Y-axis) is 9 Mbp long. Zoomed image on the right uses equal scaling and is centered on the Dicer locus. The sequences were aligned with nucmer (--nosimplify) and plotted with mummerplot (--filter).


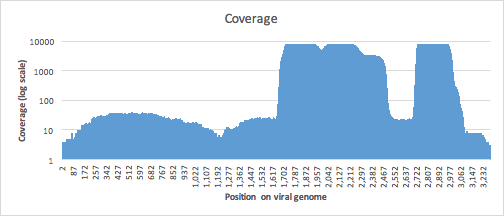


**Figure S4**. Read coverage of a virus integrant. Illumina reads from C6/36 genomic DNA, providing 20X assembly coverage, were mapped to GenBank AY223844.1, a virus-like sequence previously isolated from C6/36 genomic DNA. Read pairs joined by FLASH (8) were mapped with bowtie2 (2) parameterized for fast end-to-end alignment. Coverage per base was computed with ‘samtools depth’ (3). It appears that the 3’ half of the sequence is present at higher-copy than the 5’ half. Note use of the log scale on the Y-axis.

**REFERENCES FOR SUPPLEMENTAL FIGURES**

1. Vurture GW, Sedlazeck FJ, Nattestad M, Underwood CJ, Fang H, Gurtowski J, et al. GenomeScope: Fast reference-free genome profiling from short reads. Bioinformatics. 2017.

2. Langmead B, Salzberg SL. Fast gapped-read alignment with Bowtie 2. Nature methods. 2012;9(4):357-9.

3. Li H, Handsaker B, Wysoker A, Fennell T, Ruan J, Homer N, et al. The Sequence Alignment/Map format and SAMtools. Bioinformatics. 2009;25(16):2078-9.

4. Kurtz S, Phillippy A, Delcher AL, Smoot M, Shumway M, Antonescu C, et al. Versatile and open software for comparing large genomes. Genome biology. 2004;5(2):R12.

5. Morazzani EM, Wiley MR, Murreddu MG, Adelman ZN, Myles KM. Production of virus-derived ping-pong-dependent piRNA-like small RNAs in the mosquito soma. PLoS Pathog. 2012;8(1):e1002470.

6. Chaisson MJ, Tesler G. Mapping single molecule sequencing reads using basic local alignment with successive refinement (BLASR): application and theory. BMC bioinformatics. 2012;13(1):238.

7. Robinson JT, Thorvaldsdottir H, Winckler W, Guttman M, Lander ES, Getz G, et al. Integrative genomics viewer. Nat Biotechnol. 2011;29(1):24-6.

8. Magoč T, Salzberg SL. FLASH: fast length adjustment of short reads to improve genome assemblies. Bioinformatics. 2011;27(21):2957-63.
