# Supplementary material for: Analysis of the Aedes albopictus C6/36 genome provides insight into cell line utility for viral propagation
Source: Gigascience. 2018 Jan 10;7(3):gix135. doi: 10.1093/gigascience/gix135 (PMC5869287; doi:10.1093/gigascience/gix135)
Supplement: Response_to_Reviewer_Comments_Original_Submission.doc [file gix135_response_to_reviewer_comments_original_submission.doc]

### Author's Response To Reviewer Comments

Top of Form

Bottom of Form

Authors’ response to the Reviewers: We are grateful to the reviewers for their thoughtful feedback. We have implemented all of the Reviewer’s suggestions. The revised manuscript is improved in very important ways. Our guide to changes is included here. Thank you.


Reviewer 1, Summary. In the manuscript, Miller and colleagues report their results on the genome assembly of the Aedes albopictus C6/36 cell line, an important laboratory cell line resource for studying mosquito-borne viruses. The author applied long-read PacBio DNA sequencing and Illumina NextSeq RNA sequencing to assemble and annotate the genome and transcriptome of the cell line. The analysis is appropriate and uses a range of computational programs for de novo assembly, repeat detection, genome annotation, RNA mapping, and cross-comparisons to a previous A. albopictus Foshan assembly. The results are valuable resources for future molecular studies of virus propagation and virus-mosquito vector interactions of several significant human-disease causing viruses, including Zika, Dengue, and Chikungunya viruses. I have several comments and suggestions below:

Reviewer 1, Point 1. The title "...insight into cell line adaptation..." needs to be revised, as the results presented are not sufficient to prove the adaptation of the current cell line compared to the original parental cell line for virus propagation. Direct comparisons to the parental cell line and better links to virus propagation are needed to prove the adaptation process.

Response:
This is a valid point and we agree. We have changed the title to remove the reference to adaptation: Analysis of the Aedes albopictus C6/36 genome provides insight into cell line utility for viral propagation.

Reviewer 1, Point 2. A limitation of the PacBio long read sequencing is the high sequencing error rate, and potential readers would be interested in the accuracy of the assembly. The current manuscript presents many results on the contig length and the coverage, but there is little discussion on the accuracy. The authors should discuss this aspect. The authors may consider moving one or more of the 17 supplementary tables into the main text to highlight their key findings. Likewise, Figure S3 may be considered moving into the main text.

Response:
It was an oversight to omit consensus accuracy analysis. In our initial submission, the lone indicator of consensus quality was present in Supplemental Table S4 which showed mismatch rate = 0.70 in mapped Illumina reads. We have now included new analysis in the main text by adding a paragraph to the end the Sequencing and Assembly section of Results. Unfortunately, we lack references with which to derive the average QV per assembled base. Instead, we present statistics showing a high level of agreement with the short read sequencing which was not used during assembly: The mapped reads covered 98.98% of assembled bases and confirmed 99.30% of aligned bases; 74.66% of mapped reads aligned end-to-end with zero mismatches and indels.
We agree that some readers may be surprised to see high consensus accuracy in an assembly of high-error reads. To address this, we added a reference to our prior study of consensus quality in Canu assemblies in which consensus quality surpassed QV=30 (99.9% accuracy) on several data sets: These results are consistent with prior analyses, e.g. 99.98% identity to the Drosophila melanogaster reference achieved by a Canu+Quiver assembly of 90X P5C3 PacBio [19].

The Reviewer suggested including some of our supplemental tables or figures in the main text. We have acted on the Reviewer’s specific suggestion to move Supplemental Figure S3, to the main text. The text now includes a new Figure 3 formed from a montage of two figures from Figure S3, which remains in the supplement unchanged. Figure 3, which is about the Dicer locus, covers one of our important gene findings and simultaneously illustrates the haplotype separated nature of our assembly.

Reviewer 1, Point 3. Table 2 shows weak results on the enrichment of the two broad categories: cytoskeletal functions and cell signaling. The suggestion that these two categories can be cell line specialisations for growth in the laboratory culture should be raised with caution. DAVID analysis based on 1,310 highly expressed transcripts (with selected thresholds >=2x the RPKM means and enrichment score >1.3) and manual categorisation of 22 functional clusters are not rigorous statistical analysis. Selection of highly expressed genes may lead to a biased analysis of abundant structural genes (cytoskeleton genes), metabolic genes, and house-keeping genes such as those for ribosomal genes (the top most enriched). More stringent gene-ontology enrichment or network analysis (with appropriate background genes, more specific sub/child ontologies, and possible comparisons with other cell lines as base lines) are needed to support the conclusion on the enrichment of the two broad categories
(cytoskeletal function and cell signaling).

Response:
We thank the reviewer for this comment. We agree that a more comprehensive analysis is desirable. We plan to pursue future studies focused on the transcriptomics of this cell line. Given the limited nature of our transcriptomics data, our preference is to maintain our current analysis, which allows to compare and contrast with a previously published study by our group on Ae. albopictus Malpighian tubules. Nevertheless, we have softened our language when describing and interpreting the DAVID analysis to emphasize the preliminary nature of these results and need to confirm in future studies, and we added the conclusion: Further studies are required to test this hypothesis.

Reviewer 1, Point 4. The analysis and evidence to support the conclusion that the cell line is deficient in aquaporins and inward rectifier K+ channel need to be described with more details. Was the analysis based on the expression values (RPKM) measured from a single RNA sequencing sample? Were multiple mapped reads to AQP and Kir genes removed by the mapping and transcript counting pipelines (This may lead to low RPKM for these genes)? More biological/technical replicates may confirm whether the genes were not expressed or lowly expressed. Comparing with data from other studies on the expression of AQP and Kir genes relative to other more abundant genes may help to support the conclusion.

Response:
The reviewer requests additional details on the experiment that was done and suggests additional comparisons that should be done.
We agree that additional data would be helpful but we regret that we are not able to provide it due to funding constraints. Instead, we have softened the statement on the deficiency of AQPs and Kir channels, which we agree had too strong of a conclusion. The transcriptomics data certainly suggest weak expression of these mRNAs, but we now emphasize that additional functional studies are required to confirm weak physiological activity of these channels.
The requested details were possibly already present in the manuscript. The text at the start of the transcriptomics results section says transcripts were tested for presence or absence based on a single run of RNAseq and that RPKM was computed per transcript without consolidation per gene. We have revised our manuscript so that readers will be referred to these methods descriptions. Specifically, we added this sentence at the end of the transcriptomics results section: Our findings, derived from analysis of a single RNAseq run as described above, warrant further study. Also, we added the detail that the mapping retained at most one mapping per read, which was previously mentioned only in the methods section.

Reviewer 1, Point 5. The implications of a subtraction database for viral detection is interesting. The authors may add discussion on how the multiplex sequencing method with the use of the subtraction database outperforms the quantitative PCR method in cost, sensitivity, and time.

Response:
If the question is whether a specific virus is present, the qPCR method would be preferable by cost, sensitivity, and time. The subtraction method is preferable if the question is what viruses, if any, are present. The subtraction method could be considered a filter for a metagenomics analysis. To clarify this point for readers, we have added text to the section on subtraction results. The new text emphasizes that qPCR was included as a control here but it would not be part of an actual subtraction experiment: After this control measure, the remainder of the experiment emulated a search for any virus in cells exposed to an uncharacterized sample.

Reviewer 1, Point 6. Figure 2 axis labels should be included in the figure, not in the legend.

Response:
Figure 2 has been revised. Thank you for this suggestion, which we also applied to the new Figure 3.


Reviewer 2, Summary. Cell lines are critical platforms for understanding biology of an organism; however, over the time the cell lines drift from the genome. In order to achieve meaningful results, it is important to know the cell and tissue types the cell lines were originally derived from. The manuscript sheds light on two important considerations in the use of a cell line for understanding mosquito-virus interaction:
1. It has drifted from the Ae. albopictus genome and shows lack of aquaporin transcripts which is important to know if a researcher is interested in these genes.
2. The cell line is potentially derived from male larvae and therefore might not be suitable for testing female specific gene pathways. As the authors' suggested this finding can potentially be used for testing sex-specific agents developed for mosquito sterility.

Reviewer 2, Point 1. This is important work and worthy of publication. However, I am not fully convinced that the library prep and sequencing at two different institutions (DNA from lot # 59479117 was sequenced at NBACC (Fort Detrick, MD) using 128 SMRT cells and multiple libraries. DNA from lot #62871143 was sequenced at Icahn School of Medicine at Mt. Sinai (NY) using 80 SMRT cells) will provide data for direct comparison. The differences in sample handling and library prep will result in variations; however, I am of the opinion that it will not change the authors' conclusion. The authors still need to explain why the sequencing was carried out at two different sites.

Response:
The C6/36 assembly is derived from two lots of cells, both labeled C6/36 and provided by ATCC. Each was sequenced at a different lab but both used the PacBio RS II instrument with P6C4 chemistry and the manufacturer’s recommended prep. The reason for the separate origins of cells is that our experiment represents a merger of two formerly independent projects. Both projects had similar designs and both were underway when the project leaders discovered the redundancy during discussions at a conference. Our groups decided to pool resources in order to generate a high-quality assembly that would exploit roughly twice the sequencing depth as either project could provide separately. We hope the community benefits from our collaboration.
We added the following historical and parenthetical remark to the description of sequencing in the Methods section: (Two formerly independent sequencing projects combined resources to generate one high-coverage assembly.)
We also added a sentence to assuage concerns that the two lots may have assembled separately. We added this sentence to the Methods section near the text that contained the description of the separate lots: With the exception of 30 contigs representing 1% of assembled bases, all contigs contained reads from both samples.

Other changes:
The original draft included the claim that C6/36 was the second cell line to have its genome de novo assembled. One of us since found three publications on the CHO cell line. We revised the Discussion section to cite the publications of CHO and HeLa genomes. The revised text makes no claim to rank order: C6/36 joins CHO [83,84,85] and HeLa [55] as another cell line to have its genome de novo assembled. The new references were added to the end of the current references to make clear which references are new. We will renumber all references prior to publication.
We revised the supplemental material to reflect an update to RefSeq that occurred after our prior manuscript submission. The RefSeq annotation of the C6/36 Dicer locus formerly described a putatively transcribed gene with the note that the contig consensus sequence was missing one base. The revised RefSeq annotation accepts the consensus and describes a pseudogene. Therefore, we revised the caption to Supplemental Figure S3c-d to include the sentence: We have updated the RefSeq annotation to note a “polymorphic pseudogene” at LOC109403945.
We inserted the word “RNAseq” where it could add clarity to descriptions of our mapped RNA sequencing data.
We added accessions where it could help users find the data at NCBI. We inserted RefSeq accessions with prefixes “LOC”, “XP”, and “NW” at various places in the main text. We added Supplemental Table S18 that lists the GenBank/MNAF and RefSeq/NW accessions for each contig.
We revised the caption to Figure 1, which is about karyotypes, to improve the clarity of the text.
We updated the affiliations for several authors.
