# Supplementary material for: Analysis of the Aedes albopictus C6/36 genome provides insight into cell line utility for viral propagation
Source: Gigascience. 2018 Jan 10;7(3):gix135. doi: 10.1093/gigascience/gix135 (PMC5869287; doi:10.1093/gigascience/gix135)
Supplement: Reviewer_1_Report_(Original_Submission) -- Quan Nguyen — 20 Aug 2017 Reviewed [file gix135_reviewer_1_report_(original_submission).docx]

**Reviewer Report**

**Title:** Analysis of the Aedes albopictus C6/36 genome provides insight into cell line utility for viral propagation

**Version:** Original Submission	**Date:** 20 Aug 2017

**Reviewer name:** Quan Nguyen

**Reviewer Comments to Author:**

In the manuscript, Miller and colleagues report their results on the genome assembly of the Ades albopictus C6/36 cell line, an important laboratory cell line resource for studying mosquito-borne viruses. The author applied long-read PacBio DNA sequencing and Illumina NextSeq RNA sequencing to assemble and annotate the genome and transcriptome of the cell line. The analysis is appropriate and uses a range of computational programs for de novo assembly, repeat detection, genome annotation, RNA mapping, and cross-comparisons to a previous A. albopictus Foshan assembly. The results are valuable resources for future molecular studies of virus propagation and virus-mosquito vector interactions of several significant human-disease causing viruses, including Zika, Dengue, and Chikungunya viruses. I have several comments and suggestions below:

1. The title "...insight into cell line adaptation..." needs to be revised, as the results presented are not sufficient to prove the adaptation of the current cell line compared to the original parental cell line for virus propagation. Direct comparisons to the parental cell line and better links to virus propagation are needed to prove the adaptation process.

2. A limitation of the PacBio long read sequencing is the high sequencing error rate, and potential readers would be interested in the accuracy of the assembly. The current manuscript presents many results on the contig length and the coverage, but there is little discussion on the accuracy. The authors should discuss this aspect. The authors may consider moving one or more of the 17 supplementary tables into the main text to highlight their key findings. Likewise, Figure S3 may be considered moving into the main text.

3. Table 2 shows weak results on the enrichment of the two broad categories: cytoskeletal functions and cell signalling. The suggestion that these two categories can be cell line specialisations for growth in the laboratory culture should be raised with caution. DAVID analysis based on 1,310 highly expressed transcripts (with selected thresholds >=2x the RPKM means and enrichment score >1.3) and manual categorisation of 22 functional clusters are not rigorous statistical analysis. Selection of highly expressed genes may lead to a biased analysis of abundant structural genes (cytoskeleton genes), metabolic genes, and house-keeping genes such as the those for ribosomal genes (the top most enriched). More stringent gene-ontology enrichment or network analysis (with appropriate background genes, more specific sub/child ontologies, and possible comparisons with other cell lines as base lines) are needed to support the conclusion on the enrichment of the two broad categories (cytoskeletal function and cell signalling).

4. The analysis and evidence to support the conclusion that the cell line is deficient in aquaporins and inward rectifier K+ channel need to be described with more details. Was the analysis based on the expression values (RPKM) measured from a single RNA sequencing sample? Were multiple mapped reads to AQP and Kir genes removed by the mapping and transcript counting pipelines (This may lead to low RPKM for these genes)? More biological/technical replicates may confirm whether the genes were not expressed or lowly expressed. Comparing with data from other studies on the expression of AQP and Kir genes relative to other more abundant genes may help to support the conclusion.

5. The implications of a subtraction database for viral detection is interesting. The authors may add discussion on how the multiplex sequencing method with the use of the subtraction database outperforms the quantitative PCR method in cost, sensitivity, and time.

6. Figure 2 axis labels should be included in the figure, not in the legend.

**Methods**

Are the methods appropriate to the aims of the study, are they well described, and are necessary controls included? Yes

**Conclusions**

Are the conclusions adequately supported by the data shown? Yes

**Reporting Standards**

Does the manuscript adhere to the journal’s guidelines on [minimum standards of reporting?](https://academic.oup.com/gigascience/pages/Minimum_Standards_of_Reporting_Checklist) Yes

**Statistics**

Are you able to assess all statistics in the manuscript, including the appropriateness of statistical tests used? Yes, and I have assessed the statistics in my report.

**Quality of Written English**

Please indicate the quality of language in the manuscript: Acceptable

**Declaration of Competing Interests**

Please complete a declaration of competing interests, considering the following questions:

- Have you in the past five years received reimbursements, fees, funding, or salary from an organisation that may in any way gain or lose financially from the publication of this manuscript, either now or in the future?
- Do you hold any stocks or shares in an organisation that may in any way gain or lose financially from the publication of this manuscript, either now or in the future?
- Do you hold or are you currently applying for any patents relating to the content of the manuscript?
- Have you received reimbursements, fees, funding, or salary from an organization that holds or has applied for patents relating to the content of the manuscript?
- Do you have any other financial competing interests?
- Do you have any non-financial competing interests in relation to this paper?

If you can answer no to all of the above, write 'I declare that I have no competing interests' below. If your reply is yes to any, please give details below.

I declare that I have no competing interests

I agree to the open peer review policy of the journal. I understand that my name will be included on my report to the authors and, if the manuscript is accepted for publication, my named report including any attachments I upload will be posted on the website along with the authors' responses. I agree for my report to be made available under an Open Access Creative Commons CC-BY license (http://creativecommons.org/licenses/by/4.0/). I understand that any comments which I do not wish to be included in my named report can be included as confidential comments to the editors, which will not be published.

I agree to the open peer review policy of the journal

To further support our reviewers, we have joined with Publons, where you can gain additional credit to further highlight your hard work (see: https://publons.com/journal/530/gigascience). On publication of this paper, your review will be automatically added to Publons, you can then choose whether or not to claim your Publons credit. I understand this statement.

Yes
