# Supplementary material for: Analysis of the Aedes albopictus C6/36 genome provides insight into cell line utility for viral propagation
Source: Gigascience. 2018 Jan 10;7(3):gix135. doi: 10.1093/gigascience/gix135 (PMC5869287; doi:10.1093/gigascience/gix135)
Supplement: Reviewer_2_Report_(Original_Submission) -- Monika Gulia-Nuss — 12 Sep 2017 Reviewed [file gix135_reviewer_2_report_(original_submission).docx]

**Reviewer Report**

**Title:** Analysis of the Aedes albopictus C6/36 genome provides insight into cell line utility for viral propagation

**Version:** Original Submission	**Date:** 12 Sep 2017

**Reviewer name:** Monika Gulia-Nuss

**Reviewer Comments to Author:**

Cell lines are critical platforms for understanding biology of an organism; however, over the time the cell lines drift from the genome. In order to achieve meaningful results, it is important to know the cell and tissue types the cell lines were originally derived from. The manuscript sheds light on two important considerations in the use of a cell line for understanding mosquito-virus interaction:
1. It has drifted from the Ae, albopictus genome and shows lack of aquaporin transcripts which is important to know if a researcher is interested in these genes.
2. The cell line is potentially derived from male larvae and therefore might not be suitable for testing female specific gene pathways. As the authors' suggested this finding can potentially be used for testing sex-specific agents developed for mosquito sterility.

This is important work and worthy of publication.
However, I am not fully convinced that the library prep and sequencing at two different institutions (DNA from lot # 59479117 was sequenced at NBACC (Fort Detrick, MD) using 128 SMRT cells and multiple libraries. DNA from lot #62871143 was sequenced at Icahn School of Medicine at Mt. Sinai (NY) using 80 SMRT cells) will provide data for direct comparison. The differences in sample handling and library prep will result in variations; however, I am of the opinion that it will not change the authors' conclusion. The authors still need to explain why the sequencing was carried out at two different sites.

**Methods**

Are the methods appropriate to the aims of the study, are they well described, and are necessary controls included? Yes

**Conclusions**

Are the conclusions adequately supported by the data shown? Yes

**Reporting Standards**

Does the manuscript adhere to the journal’s guidelines on [minimum standards of reporting?](https://academic.oup.com/gigascience/pages/Minimum_Standards_of_Reporting_Checklist) Yes

**Statistics**

Are you able to assess all statistics in the manuscript, including the appropriateness of statistical tests used? No, I do not feel adequately qualified to assess the statistics.

**Quality of Written English**

Please indicate the quality of language in the manuscript: Acceptable

**Declaration of Competing Interests**

Please complete a declaration of competing interests, considering the following questions:

- Have you in the past five years received reimbursements, fees, funding, or salary from an organisation that may in any way gain or lose financially from the publication of this manuscript, either now or in the future?
- Do you hold any stocks or shares in an organisation that may in any way gain or lose financially from the publication of this manuscript, either now or in the future?
- Do you hold or are you currently applying for any patents relating to the content of the manuscript?
- Have you received reimbursements, fees, funding, or salary from an organization that holds or has applied for patents relating to the content of the manuscript?
- Do you have any other financial competing interests?
- Do you have any non-financial competing interests in relation to this paper?

If you can answer no to all of the above, write 'I declare that I have no competing interests' below. If your reply is yes to any, please give details below.

None

I agree to the open peer review policy of the journal. I understand that my name will be included on my report to the authors and, if the manuscript is accepted for publication, my named report including any attachments I upload will be posted on the website along with the authors' responses. I agree for my report to be made available under an Open Access Creative Commons CC-BY license (http://creativecommons.org/licenses/by/4.0/). I understand that any comments which I do not wish to be included in my named report can be included as confidential comments to the editors, which will not be published.

I agree to the open peer review policy of the journal

To further support our reviewers, we have joined with Publons, where you can gain additional credit to further highlight your hard work (see: https://publons.com/journal/530/gigascience). On publication of this paper, your review will be automatically added to Publons, you can then choose whether or not to claim your Publons credit. I understand this statement.

Yes
